# Safety of pharmacologic interventions for neuropsychiatric symptoms in dementia: a systematic review and network meta-analysis

**DOI:** 10.1186/s12877-020-01607-7

**Published:** 2020-06-16

**Authors:** Jennifer A. Watt, Zahra Goodarzi, Areti Angeliki Veroniki, Vera Nincic, Paul A. Khan, Marco Ghassemi, Yuan Thompson, Yonda Lai, Victoria Treister, Andrea C. Tricco, Sharon E. Straus

**Affiliations:** 1 Knowledge Translation Program, Li Ka Shing Knowledge Institute, St. Michael’s Hospital, 209 Victoria Street, East Building, Toronto, Ontario M5B 1W8 Canada; 2 Division of Geriatric Medicine, Department of Medicine, University of Toronto, 190 Elizabeth Street, R. Fraser Elliott Building, 3-805, Toronto, Ontario M5G 2C4 Canada; 3 Division of Geriatric Medicine, Department of Medicine, University of Toronto, 6 Queen’s Park Cres W, Toronto, Ontario M5S 3H2 Canada; 4 Hotchkiss Brain Institute, University of Calgary, 3330 Hospital Dr NW, Calgary, Alberta T2N 4N1 Canada; 5 O’Brien Institute of Public Health, University of Calgary, 3280 Hospital Dr NW, Calgary, Alberta T2N 4Z6 Canada; 6 Department of Primary Education, School of Education, University of Ioannina, 45110 Ioannina, Greece; 7 Institute of Reproductive and Developmental Biology, Department of Surgery & Cancer, Faculty of Medicine, Imperial College, W12 0NN, London, UK; 8 Institute for Health Policy, Management and Evaluation, University of Toronto, 4th floor, 155 College St, Toronto, Ontario M5T 3M6 Canada

**Keywords:** Systematic review, Meta-analysis, Network meta-analysis, Dementia, Harm, Neuropsychiatric symptoms

## Abstract

**Background:**

Prescribing trends suggest that pharmacologic alternatives to antipsychotics are gaining in popularity, but randomized trial (RCT) data of their comparative safety is scarce. Our objective was to describe the comparative safety of pharmacologic interventions for treating neuropsychiatric symptoms in dementia.

**Methods:**

We searched MEDLINE, EMBASE, CENTRAL, CINAHL, and PsycINFO, from inception to May 28, 2019, for studies of pharmacologic interventions used to treat neuropsychiatric symptoms in dementia. Dementia care partners selected fracture risk as our primary outcome. Pairs of reviewers, working independently, conducted all study screening, data abstraction, and risk of bias appraisal. We conducted Bayesian random-effects network meta-analyses (NMAs) using data from RCTs to derive odds ratios (ORs). In secondary analyses, we conducted frequentist random-effects NMAs using data from RCTs and Bayesian three-level hierarchical random-effects NMAs incorporating data from RCTs and non-randomized studies.

**Results:**

Our systematic review included 209 randomized and non-randomized studies (889,378 persons with dementia). In NMAs of data from randomized trials, there were no increased odds of fracture associated with any intervention in primary analyses; however, data were sparse. We found increased odds of cerebrovascular events associated with antipsychotics (odds ratio [OR] 2.12, 95% credible interval [CrI] 1.29 to 3.62; number needed to harm [NNH] = 99) and increased odds of falls associated with dextromethorphan-quinidine (OR 4.16, 95% CrI 1.47 to 14.22; NNH = 55) compared to placebo in persons with dementia. In a subgroup of persons with Alzheimer disease, antipsychotics were associated with increased odds of fracture compared to anticonvulsants (OR 54.1, 95% CrI 1.15 to 38,300; NNH = 18). In older persons (mean age ≥ 80 years) with dementia, anticonvulsants were associated with increased odds of death compared to placebo (OR 8.36, 95% CrI 1.17 to 203.4; NNH = 35) and antipsychotics were associated with increased odds of death compared to antidepressants (OR 5.28, 95% CrI 1.06 to 3.51; NNH = 47).

**Conclusion:**

Although antipsychotics were associated with greater harm than antidepressants and anticonvulsants in subgroups of persons with dementia, medications used *in lieu* of antipsychotics for treating neuropsychiatric symptoms in dementia, such as anticonvulsants and dextromethorphan-quinidine, were also associated with harm. Decision-making concerning treatments prescribed *in lieu* of antipsychotics should include potential harms.

**PROSPERO registration:**

CRD42017050130.

## Background

There are 50 million people worldwide living with dementia [1]. Pharmacologic (e.g. antidepressants and antipsychotics) and nonpharmacologic (e.g. recreation therapy) interventions are administered as treatment for cognitive and neuropsychiatric symptoms associated with dementia [2–4]. In persons with dementia, pharmacologic interventions, most notably antipsychotics, have been associated with significant risk including cerebrovascular accidents and death [5, 6]. Studies show that as clinicians adopt recommendations of clinical practice guidelines and de-prescribing initiatives to taper and discontinue antipsychotics in persons living with dementia, alternative pharmacologic interventions (e.g. antidepressants, anticonvulsants) are prescribed to treat neuropsychiatric symptoms rather than nonpharmacologic alternatives, despite their demonstrated efficacy [7–11]. Pharmacologic interventions continue to be prescribed by clinicians for many reasons, including perceived patient, staff, and system barriers to implementation of nonpharmacologic interventions in busy clinical settings [12].

Given the high prescription rates of pharmacologic interventions for cognitive and neuropsychiatric symptoms of dementia and the paucity of head-to-head randomized trials (RCTs) that knowledge users (e.g. patients, caregivers, and clinicians) can use to inform shared decision-making, we conducted a network meta-analysis (NMA) to fill this critical knowledge gap [13]. Our objectives were to: (1) determine the comparative risks of: fracture, mortality, cerebrovascular events, and falls associated with pharmacologic interventions dispensed for treating neuropsychiatric symptoms in dementia; and (2) establish the safest pharmacologic interventions to treat neuropsychiatric symptoms in dementia.

## Methods

Our protocol was registered (Prospero: CRD42017050130) and published [14]. We report our findings in accordance with the PRISMA extension statement for reporting systematic reviews incorporating NMA [8]. We discuss protocol deviations in Additional file 1 [14].

### Eligibility criteria

We included RCTs and non-randomized studies (NRSs) of pharmacologic interventions used to treat neuropsychiatric symptoms in persons with any type of dementia. Pharmacologic interventions were limited to those with final approval from the US Food and Drug Administration or Health Canada, as of our literature search date. Eligible comparator groups included placebo, usual care, or another approved pharmacologic intervention.

### Outcomes

We asked 12 dementia care partners (e.g. nurses, physicians, a caregiver) to rank commonly reported safety outcomes associated with the treatment of neuropsychiatric symptoms in dementia [14, 15]. Risk of fracture was selected as our primary outcome. The next most highly ranked outcomes were risk of fall, cerebrovascular event, and death.

### Data sources, study selection, data abstraction, and risk of bias appraisal

An experienced librarian searched MEDLINE, EMBASE, CENTRAL, CINAHL, and PsycINFO for citations published in any language from inception until May 28, 2019 [14]. Our search strategy was peer reviewed by a second librarian using the Peer Review of Electronic Search Strategies checklist [16]. We searched the grey literature, reference lists of included studies, and related systematic reviews (Additional file 1). After pilot-testing, pairs of reviewers (JAW, ZG, VN, PAK, MG, and YT) independently screened all citations and full-text articles to establish eligibility for inclusion, abstracted data from included full-text articles, and appraised each study for risk of bias. Where available, we abstracted study-level estimates (e.g. hazard ratios [HR]) from NRSs that adjusted for confounders. RCTs were appraised with the Cochrane Risk of Bias Tool [17]. Cohort and case-control studies were appraised with the Newcastle-Ottawa quality assessment scales [18]. Other NRSs were appraised with the Cochrane Effective Practice and Organisation of Care Tool [19].

### Data synthesis and analysis

We formed treatment nodes based on drug classes, irrespective of treatment dose: placebo/control, antidepressants, anticonvulsants, N-methyl-D-aspartate (NMDA) receptor antagonist (memantine), cholinesterase inhibitors, antipsychotics, anxiolytic/hypnotics, anddextromethorphan-quinidine. We assessed network transitivity by visual inspection of study and patient characteristics across treatment comparisons: study size, patient age, proportion of females, study setting (e.g. nursing home or community), study duration, type of dementia, severity of dementia, and two items from the risk of bias assessment (missing data and method of randomization). We prepared network diagrams in Stata, version15.1 (StataCorp) [20].

Our primary analyses for each outcome were Bayesian random-effects NMAs, which were conducted in OpenBUGS, version 3.2.3 [21]. We conducted a Bayesian random-effects pairwise meta-analysis where there was more than one study per treatment comparison. We used random-effects models because we anticipated between-study heterogeneity. We assumed a single within-network between-study variance in each NMA model because all treatments were used in a population of persons living with dementia. Informative prior distributions were implemented for all between-study heterogeneity parameters for the outcome of mortality (τ^2^ ~ log-normal (− 4.06,1.45^2^); and for the outcomes of fracture, fall, and cerebrovascular event (τ ^2^ ~ log-normal (− 3.02,1.85^2^)) [22]. We implemented vague prior distributions for all trial baselines and treatment differences (normal (0,1000)). Outcomes were reported as odds ratios (ORs) with 95% credible intervals (CrIs) and predictive intervals (PrIs). We ranked treatments using surface under the cumulative ranking curve (SUCRA) values and summarized treatment rankings across outcomes in a rank-heat plot [23]. The number needed to harm (NNH) and number needed to treat (NNT) were calculated as the inverse of the difference in absolute risk between two intervention groups. We assessed global consistency with a design-by-treatment interaction model and local consistency with a loop-specific approach [24, 25].

In secondary analyses, we conducted frequentist random-effects network and pairwise meta-analyses for each outcome in Stata, version 15.1 (StataCorp) [20]. Frequentist approaches are most commonly reported in the medical literature and we wanted to facilitate interpretation of our findings [26]. We did not conduct frequentist analyses for subgroup or sensitivity analyses. We estimated the common within-network between-study variance with the restricted maximum likelihood method. Outcomes were reported as ORs and 95% confidence intervals (CIs).

To understand the impact of NRSs, we conducted Bayesian three-level hierarchical random-effects, shared parameter NMAs incorporating data from RCTs and NRSs [27]. We implemented vague priors for between-study heterogeneity parameters for NRSs (τ ~ normal (0,100), τ > 0), informative priors for between-study heterogeneity parameters for RCTs, and weakly informative priors for the between-study type heterogeneity parameter (τ ~ normal (0,1), τ > 0) [22, 27, 28]. Outcomes were reported as relative risks (RRs). NRSs frequently reported HRs and ORs, which we approximated as RRs [29]. We assessed local consistency in these models with a loop-specific approach [24].

We conducted subgroup analyses based on the following effect modifiers: average age of study population ≥ vs. ≤80 years old, proportion of women ≥ vs. < 50%, Alzheimer type of dementia, severity of dementia (mild/moderate vs. moderate/severe), study setting (long-term care facility vs. community), and study duration (< 13 weeks vs. ≥13 weeks). We completed a meta-regression based on publication year. We conducted sensitivity analyses by removing studies where data were found in a secondary data source (e.g. systematic review or clinical trial registry). We also conducted sensitivity analyses by removing studies at high or unclear risk of bias based on the two components of the risk of bias assessment that were the greatest threat to the validity of study findings: incomplete outcome data and randomization procedure. We conducted a sensitivity analysis using a weakly informative prior for the between-study heterogeneity (τ ~ N(0,1), τ > 0) in primary analyses. We assessed for publication bias by visually inspecting comparison-adjusted funnel plots [20].

## Results

**Fig. 1:**
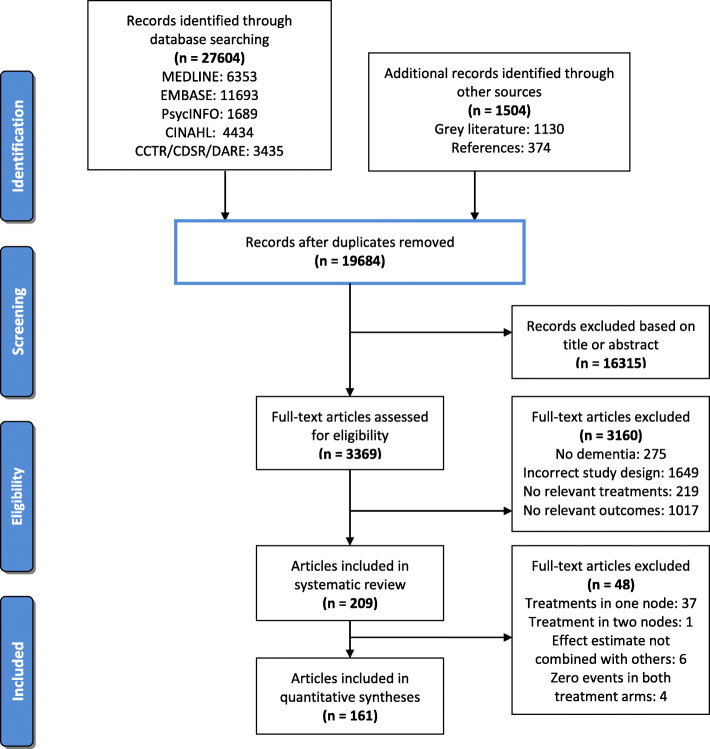
PRISMA Flow Diagram

We screened 19,684 article titles and abstracts and 3369 full-text articles, which resulted in 209 included studies (889,378 patients with dementia) (Fig. 1). Individual study and study-level patient characteristics are reported in Additional file 1 Tables 1 and 2. Arm-level data for RCTs included in our NMAs are reported in Additional file 1 Table 3. There was transitivity across treatment comparisons for each outcome (Additional file 1 Tables 4a-d). In most studies, the average age of patients was greater than 70 years and women represented greater than 50% of patients (Table 1). Only 42.6% of studies reported a measure of neuropsychiatric symptom severity. Data from 49 of 150 (32.7%) RCTs included in our systematic review were retrieved from secondary sources (Additional file 1 Table 3). Bias from missing data and unclear randomization procedures posed the greatest threats to study validity (Additional file 1 Tables 5–8 and Additional file 1 Figure 1). There was no evidence of publication bias based on visual assessment of comparison-adjusted funnel plots (Additional file 1 Figures 2a-h). There was no evidence of global inconsistency according to the design-by-treatment interaction model in any of our four NMAs (Additional file 1 Tables 9b, 9e, 9 h, 9 k). In meta-regression analyses, year of study publication was not associated with any of our outcomes.

**Table 1:** Summary of Patient and Study Characteristics of 209 Studies Included in our Systematic Review

Characteristic	Number of Studies (%)
*Age, mean (years)*	
< 70	7 (3.3)
70–79·9	116 (55.5)
≥ 80	68 (32.5)
Not Reported	18 (8.6)
*% Women*	
0–49	31 (14.8)
50–100	165 (78.9)
Not Reported	13 (6.2)
*Type of Dementia*	
Multiple (e.g. AD + VaD)	50 (23.9)
AD	110 (52.6)
VaD	10 (4.8)
Other^a^	9 (4.3)
Not Reported	30 (14.4)
*Severity of Dementia*	
Mild	2 (1)
Mild/Moderate	67 (32.1)
Mild/Moderate/Severe	36 (17.2)
Moderate	5 (2.4)
Moderate/Severe	24 (11.5)
Severe	6 (2.9)
Not Reported	69 (33)
*Study Design*	
RCT	148 (70.8)
Cohort Study	52 (24.9)
Case-Control Study	5 (2.4)
Pairwise Meta-Analysis of RCTs	2 (1)
Other Non-Randomized Study	2 (1)
*Setting*	
Clinic/Community	65 (31.1)
Hospital	7 (3.3)
Nursing home/Assisted Living	45 (21.5)
Multiple Settings	22 (10.5)
Not Reported	70 (33.5)
*Sample Size (No. of patients)*	
< 200	64 (30.6)
201–500	63 (30.1)
> 500	81 (38.8)
Not Reported	1 (0.5)
*Duration of Intervention, weeks*	
< 13	65 (31.1)
13–30	78 (37.3)
> 30	57 (27.2)
Not Reported	9 (4.3)

Abbreviations: *AD* Alzheimer’s dementia, *%* percentage, *RCT* randomized trial, *VaD* vascular dementia

^a^Other includes Lewy body dementia, Parkinson’s disease dementia, and frontotemporal dementia

### Fracture

**Fig. 2:**
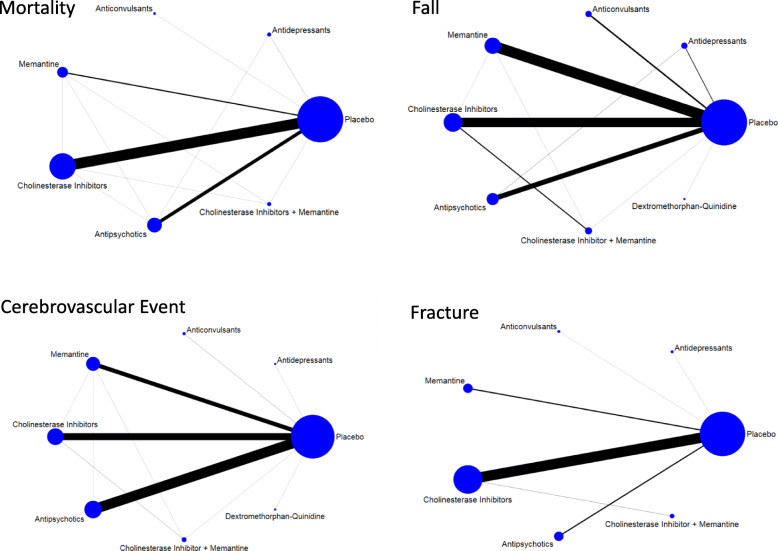
Network Diagrams Nodes represent individual interventions and nodes connected by lines indicate that these two interventions have been directly compared in a randomized trial. The nodes are weighted by number of randomized trials evaluating this treatment and lines are weighted by number of randomized trials evaluating this treatment comparison

For our primary outcome of fracture risk, 46 studies were included in our systematic review and 35 studies included in our NMAs (29 RCTs [13,410 persons with dementia] plus 6 NRSs [107,765 persons with dementia]). Fracture data from 13 RCTs were retrieved from a secondary source. In our primary analysis, the network diagram revealed a connected network with no closed loops, and between-study heterogeneity was low (Fig. 2 and Additional file 1 Figure 3a). Inclusion of NRSs resulted in one consistent, closed network loop (Additional file 1 Figure 3b).

No treatment was associated with increased odds of fractures compared with placebo in primary or sensitivity analyses (Table 2, Table 3, and Additional file 1 Tables 9a-c). In persons with moderate-severe dementia (OR 0.05, 95% CrI < 0.01 to 0.71; NNH = 14), with mild-moderate dementia (OR 0.01, 95% CrI < 0.01 to 0.74; NNT = 17), or enrolled in studies longer than 12 weeks (OR 0.01, 95% CrI < 0.01 to 0.8; NNT = 15), anticonvulsants were associated with lower odds of fracture compared to cholinesterase inhibitors+memantine. In persons with Alzheimer disease, antipsychotics (OR 54.1, 95% CrI 1.15 to 38,300; NNH = 18) and cholinesterase inhibitors+memantine (OR 72.49, 95% CrI 1.38 to 43,840; NNH = 17) were associated with increased odds of fracture compared to anticonvulsants. In a sensitivity analysis where data retrieved from secondary data sources were removed, antipsychotics were associated with lower odds of fracture compared to cholinesterase inhibitors+memantine (OR 0.06, 95% CrI < 0.01 to 0.72; NNH = 28) and cholinesterase inhibitors (OR 0.32, 95% CrI 0.11 to 0.97; NNH = 71).

**Table 2:** Treatment Effects Compared to Placebo in Primary Analyses of Randomized Trial Data: Fracture, Mortality, Cerebrovascular Event, and Fall

Intervention (vs. Placebo)	Bayesian NMA OR Estimate (95% CrI)	Frequentist NMA OR Estimate (95% CI)
*Fracture (n = 29 RCTs, 13,410 patients, 133 events)*		
Anticonvulsants	0.11 (< 0.01 to 2.10)	0.21 (0.01 to 4.34)
Antidepressants	0.16 (< 0.01 to 5.34)	0.28 (0.01 to 7.4)
Antipsychotics	0.72 (0.33 to 1.66)	0.64 (0.3 to 1.37)
Cholinesterase Inhibitors	1.34 (0.86 to 2.14)	1.29 (0.81 to 2.04)
Cholinesterase Inhibitors + Memantine	6.63 (0.75 to 209.7)	4.84 (0.51 to 46.35)
Memantine	0.58 (0.16 to 1.93)	0.64 (0.16 to 2.64)
*Mortality (n = 104 RCTs, 38,683 patients, 1037 events)*		
Anticonvulsants	1.29 (0.55 to 2.95)	1.13 (0.48 to 2.64)
Antidepressants	0.83 (0.33 to 2.25)	0.88 (0.36 to 2.16)
Antipsychotics	1.17 (0.87 to 1.6)	1.14 (0.84 to 1.55)
Cholinesterase Inhibitors	0.82 (0.68 to 1)	0.8 (0.65 to 0.98)
Cholinesterase Inhibitors + Memantine	0.85 (0.45 to 1.67)	0.86 (0.44 to 1.65)
Memantine	1.08 (0.78 to 1.45)	1.05 (0.77 to 1.44)
*Cerebrovascular Event (n = 47 RCTs, 19,070 patients, 314 events)*		
Anticonvulsants	1.04 (0.1 to 11.39)	1.03 (0.1 to 10.03)
Antidepressants	0.19 (< 0.01 to 5.85)	0.32 (0.01 to 8.23)
Antipsychotics	2.23 (1.36 to 3.79)	1.94 (1.15 to 3.27)
Cholinesterase Inhibitors	1.02 (0.72 to 1.48)	1.01 (0.72 to 1.39)
Cholinesterase Inhibitors + Memantine	1.11 (0.29 to 3.8)	0.99 (0.28 to 3.46)
Dextromethorphan-Quinidine	1.35 (0.03 to 55.23)	1.37 (0.08 to 22.18)
Memantine	0.76 (0.45 to 1.29)	0.74 (0.43 to 1.28)
*Fall (n = 58 RCTs, 21,776 patients, 1751 events)*		
Anticonvulsants	1.31 (0.89 to 1.95)	1.33 (0.94 to 1.87)
Antidepressants	1.06 (0.54 to 2.09)	1.13 (0.55 to 2.32)
Antipsychotics	0.99 (0.79 to 1.23)	0.98 (0.79 to 1.2)
Cholinesterase Inhibitors	0.93 (0.77 to 1.14)	0.94 (0.78 to 1.13)
Cholinesterase Inhibitors + Memantine	0.97 (0.63 to 1.48)	1.06 (0.68 to 1.66)
Dextromethorphan-Quinidine	4.24 (1.47 to 14.79)	3.97 (1.36 to 11.55)
Memantine	0.95 (0.77 to 1.17)	0.96 (0.79 to 1.18)

Abbreviations: *CrI* credible interval, *CI* confidence interval, *NMA* network meta-analysis, *n* number, *OR* odds ratio, *RCT* randomized trial

**Table 3:** Treatment Effects Compared to Placebo/Control in a Network Meta-Analysis Model Incorporating Randomized and Non-Randomized Studies: Fracture, Mortality, Cerebrovascular Event, and Fall

Intervention (vs. Placebo/Control)	Combined RCT + NRS RR (95% CrI)	RCT RR (95% CrI)	NRS RR (95% CrI)
*Fracture*			
Anticonvulsants	0.63 (0.05 to 6.54)	0.63 (0.08 to 4.33)	–
Antidepressants	0.82 (0.16 to 3.24)	0.74 (0.12 to 3.11)	0.94 (0.25 to 2.39)
Antipsychotics	0.89 (0.29 to 2.68)	0.77 (0.36 to 1.58)	1.05 (0.41 to 2.25)
Cholinesterase Inhibitor + Memantine	6.18 (0.5 to 325.1)	6.1 (0.7 to 294.8)	–
Cholinesterase Inhibitors	1.02 (0.36 to 2.76)	1.23 (0.81 to 1.91)	0.84 (0.48 to 1.52)
Memantine	0.59 (0.1 to 3.38)	0.59 (0.17 to 1.93)	–
*Mortality*			
Anticonvulsants	1.28 (0.82 to 1.99)	1.27 (0.81 to 2.0)	1.29 (0.85 to 1.9)
Antidepressants	0.86 (0.5 to 1.48)	0.86 (0.49 to 1.5)	0.87 (0.52 to 1.43)
Antipsychotics	1.33 (1.01 to 1.73)	1.28 (1 to 1.59)	1.38 (1.13 to 1.69)
Anxiolytic/hypnotics	0.84 (0.41 to 1.72)	–	0.84 (0.44 to 1.61)
Cholinesterase Inhibitor + Memantine	1.04 (0.68 to 1.58)	1.02 (0.66 to 1.52)	1.06 (0.72 to 1.57)
Cholinesterase Inhibitors	0.84 (0.65 to 1.1)	0.84 (0.71 to 0.99)	0.84 (0.68 to 1.04)
Memantine	1.15 (0.84 to 1.59)	1.12 (0.87 to 1.43)	1.17 (0.87 to 1.64)
*Cerebrovascular Event*			
Anticonvulsants	1.03 (0.1 to 11.56)	1.02 (0.15 to 8.05)	–
Antidepressants	0.27 (< 0.01 to 8.34)	0.28 (< 0.01 to 6.8)	–
Antipsychotics	1.76 (0.57 to 4.79)	2.02 (1.25 to 3.39)	1.51 (0.63 to 3.28)
Cholinesterase Inhibitor + Memantine	1.04 (0.16 to 5.73)	1.05 (0.27 to 3.29)	–
Cholinesterase Inhibitors	0.93 (0.3 to 2.5)	0.99 (0.72 to 1.42)	0.86 (0.33 to 1.89)
Dextromethorphan-Quinidine	1.11 (0.03 to 40.95)	1.11 (0.04 to 30.25)	–
Memantine	0.76 (0.18 to 3.11)	0.76 (0.45 to 1.28)	–
*Fall*			
Anticonvulsants	1.09 (0.26 to 4.6)	1.09 (0.64 to 1.89)	–
Antidepressants	1.32 (0.49 to 3.98)	1.16 (0.67 to 1.97)	1.59 (0.79 to 2.84)
Antipsychotics	1.21 (0.5 to 3.74)	1.05 (0.91 to 1.23)	1.54 (0.82 to 2.88)
Cholinesterase Inhibitor + Memantine	0.97 (0.24 to 3.94)	0.96 (0.66 to 1.43)	–
Cholinesterase Inhibitors	0.94 (0.24 to 3.73)	0.94 (0.79 to 1.14)	–
Dextromethorphan-Quinidine	3.66 (0.72 to 18.91)	3.65 (1.4 to 10.42)	–
Memantine	0.95 (0.24 to 3.82)	0.95 (0.79 to 1.15)	–

Abbreviations: *CrI* credible interval, *NRS* non-randomized study, *RCT* randomized trial, *RR* relative risk

### Mortality

For our secondary outcome of mortality risk, 165 studies were included in our systematic review and 130 studies included in our NMAs (104 RCTs [38,683 persons with dementia] plus 26 NRSs [211,511 persons with dementia]). Mortality data for 13 RCTs were retrieved from a secondary source. Network diagrams of (1) RCTs only and (2) RCTs and NRSs were connected and there was no evidence of inconsistency (Fig. 2 and Additional file 1 Figures 3c-d). Between-study heterogeneity was low in our NMAs of RCTs.

In older patients (mean age ≥ 80 years), anticonvulsants were associated with increased odds of mortality compared to placebo (NMA OR 8.36, 95% CrI 1.17 to 203.4; NNH = 35; pairwise meta-analysis OR 5.42, 95% CrI 1.01 to 77.23), antipsychotics were associated with increased odds of mortality compared to antidepressants (NMA OR 5.28, 95% CrI 1.06 to 35.05; NNH = 47; pairwise meta-analysis OR 11.05, 95% CrI 0.51 to 4610.42), and antipsychotics were associated with increased odds of mortality compared to cholinesterase inhibitors (OR 1.93, 95% CrI 1.06 to 3.51; NNH = 121). In studies less than 13 weeks in duration, anticonvulsants were associated with increased odds of mortality compared to antidepressants (OR 11.61, 95% CrI 1.21 to 258.6; NNH = 31) and cholinesterase inhibitors (OR 7.34, 95% CrI 1.05 to 121.92; NNH = 38).

Cholinesterase inhibitors were associated with lower odds of mortality compared to placebo (Bayesian OR 0.82, 95% CrI 0.68 to 1.00, PrI 0.52 to 1.28; NNT = 412; frequentist OR 0.80, 95% CI 0.65 to 0.98) in NMAs incorporating RCTs only (Table 2 and Additional file 1 Tables 9d-e). Direct treatment comparisons between cholinesterase inhibitors and placebo incorporated data from 48 RCTs (22,828 patients; 529 deaths). Compared to placebo, lower odds of mortality were observed in subgroups of studies conducted in nursing homes (OR 0.59, 95% CrI 0.35 to 0.97; NNT = 152) and in older patients (OR 0.59, 95% CrI 0.36 to 0.97; NNT = 152). Lower odds of mortality were also seen in the sensitivity analysis involving studies at low risk of bias from randomization (OR 0.80, 95% CrI 0.64 to 0.99; NNT = 358). Cholinesterase inhibitors were no longer associated with decreased odds of mortality when (1) RCTs in which mortality data were not found in the primary publication were removed from the NMA (OR 0.83, 95% CrI 0.68 to 1.01) or (2) we implemented a weakly informative prior (OR 0.82, 95% CrI 0.65 to 1.02).

### Cerebrovascular event

For our secondary outcome of cerebrovascular event risk, there were 66 studies included in our systematic review and 52 studies included in our NMAs (47 RCTs [19,070 persons with dementia] plus 5 NRSs [52,933 persons with dementia]). Data from 16 of 47 RCTs were retrieved from a secondary source. Network diagrams of (1) RCTs only and (2) RCTs and NRSs were connected and there was no evidence of inconsistency (Fig. 2 and Additional file 1 Figures 3e-f). Between-study heterogeneity was low in our NMA of RCTs. Antipsychotics were associated with greater odds of a cerebrovascular event compared to placebo (Bayesian OR 2.23, 95% CrI 1.36 to 3.79, PrI 1.12 to 4.49; NNH = 99; frequentist OR 1.94, 95% CI 1.15 to 3.27) in our NMA of RCTs (Table 2 and Additional file 1 Tables 9 g-h). This finding among patients randomized to receive antipsychotics compared to placebo was insensitive to our choice of prior for between-study variance (OR 2.2, 95% CrI 1.32 to 3.83). Significantly higher odds of cerebrovascular events associated with antipsychotics compared to placebo/control were not seen in NRSs (Table 3 and Additional file 1 Table 9i).

In patients with mild-moderate dementia, antipsychotics were associated with increased odds of cerebrovascular event compared to placebo (OR 4.43, 95% CrI 1.89 to 11.9; NNH = 53), memantine (OR 15.24, 95% CrI 3.33 to 86.31; NNH = 29) and cholinesterase inhibitors (OR 4.58, 95% CrI 1.75 to 13.46; NNH = 52). In RCTs at low risk of bias from randomization, antipsychotics were associated with increased odds of cerebrovascular event compared to placebo (OR 4.42, 95% CrI 2.1 to 10.29; NNH = 53), memantine (OR 5.43, 95% CrI 2.16 to 14.52; NNH = 47) and cholinesterase inhibitors (OR 4.02, 95% CrI 1.7 to 10.02; NNH = 57). And, in RCTs enrolling ≥50% women, antipsychotics were associated with increased odds of cerebrovascular events compared to placebo (OR 2.21, 95% CrI 1.33 to 3.82; NNH = 100) and memantine (OR 2.61, 95% CrI 1.26 to 5.59; NNH = 84).

### Falls

For our secondary outcome of falls risk, there were 79 studies included in our systematic review and 61 studies included in our NMAs (58 RCTs [21,776 persons with dementia] plus 3 NRSs [16,355 persons with dementia]). Network diagrams of (1) RCTs only and (2) RCTs and NRSs were connected (Fig. 2). Data from 16 of 61 RCTs were reported in a secondary source. There was one inconsistent loop of evidence in the network of RCTs and the network of RCTs and NRSs (Additional file 1 Figures 3g-h). Between-study heterogeneity was low in our NMA of RCTs. Dextromethorphan-quinidine use was associated with increased odds of falls compared to placebo (Bayesian OR 4.24, 95% CrI 1.47 to 13.79, PrI 1.44 to 14.03; NNH = 55; frequentist OR 3.97, 95% CI 1.36 to 11.55) (Table 2 and Additional file 1 Tables 9j-k). In subgroup analysis of shorter duration RCTs, dextromethorphan-quinidine was associated with increased odds of falls compared to placebo (OR 4.14, 95% CrI 1.41 to 14.09; NNH = 56), antidepressants (OR 3.64, 95% CrI 1.01 to 15.16; NNH = 61), and antipsychotics (OR 4.14, 95% CrI 1.38 to 14.32; NNH = 56) and there was no evidence of inconsistency in the network.

### Treatment rankings

**Fig. 3:**
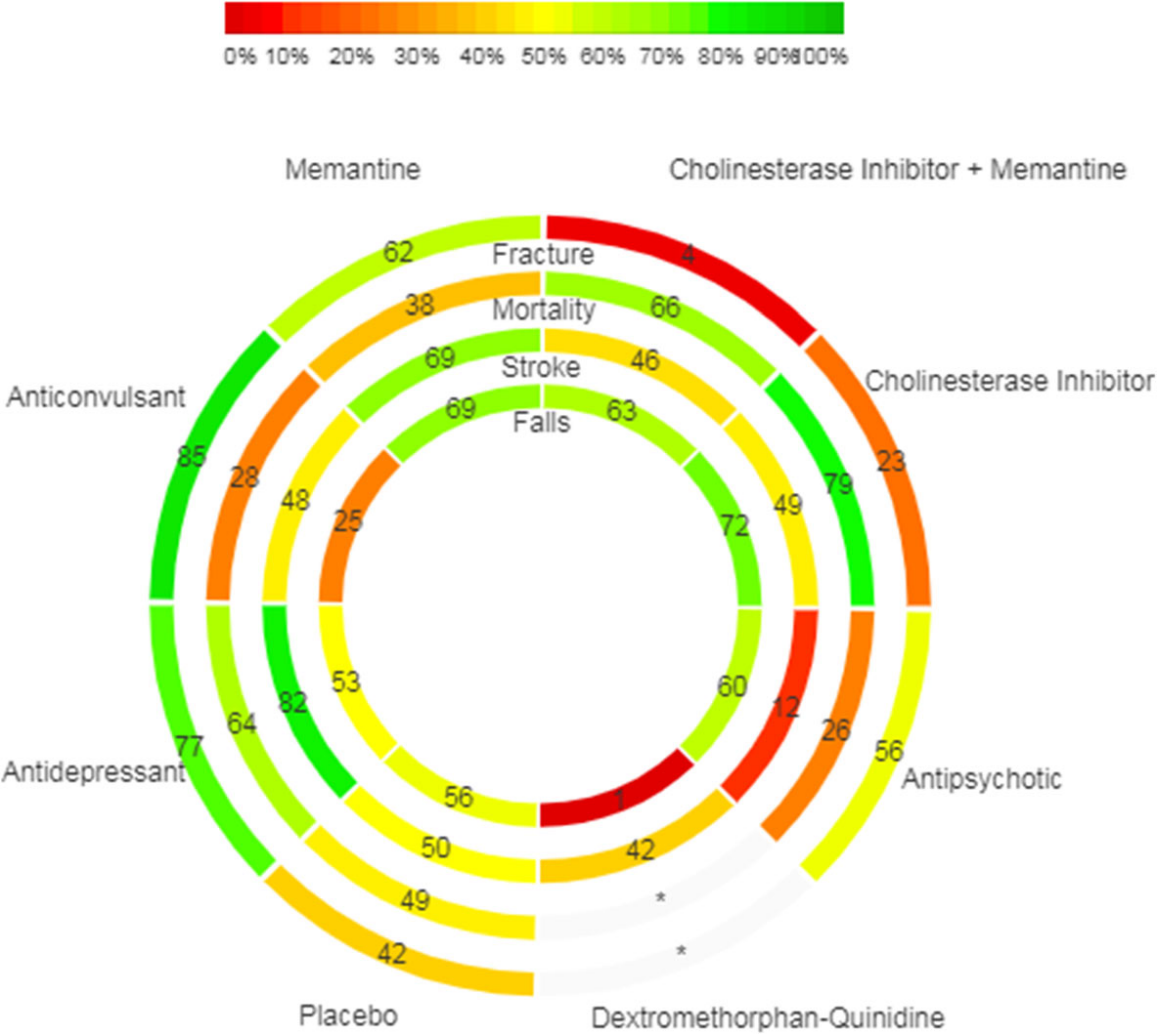
Rank-Heat Plot of Safety Outcomes Associated with Pharmacologic Interventions Prescribed to Treat Neuropsychiatric Symptoms in Persons with Dementia. The scale bar represents the Surface Under the Cumulative Ranking Curve (SUCRA) value for each intervention. The lowest SUCRA values are indicated in red (worst/most dangerous treatments) and the highest SUCRA values are indicated in green (best/safest treatments). An asterisk (*) indicates this is a treatment without data for the outcome of interest

In our primary analyses, anticonvulsants (SUCRA 85, 95% CrI 17 to 100%) were the safest pharmacological intervention with respect to fracture risk (Fig. 3). Cholinesterase inhibitors (SUCRA 79, 95% CrI 50 to 100%) were the safest pharmacological intervention with respect to risk of mortality. Antidepressants (SUCRA 82, 95% CrI 0 to 100%) were the safest pharmacological intervention with respect to risk of cerebrovascular events. Cholinesterase inhibitors (SUCRA 72, 95% CrI 29 to 100%) were the safest pharmacological interventions with respect to risk of falling.

## Discussion

Our systematic review and NMA described the comparative risk of fractures, mortality, cerebrovascular events, and falls associated with pharmacologic interventions implemented for treating neuropsychiatric symptoms in dementia. There were no pharmacologic interventions associated with increased odds of fracture; however, few studies reported fractures as an adverse event and there was large uncertainty for some effect estimates. Importantly, in older adults with dementia, we identify increased odds of mortality associated with anticonvulsants compared to placebo and antipsychotics compared to antidepressants. And, dextromethorphan-quinidine was associated with increased odds of falling compared to placebo, antidepressants, and antipsychotics. These estimates, based on RCT data, suggest that specific pharmacologic interventions dispensed for treating neuropsychiatric symptoms in dementia are associated with harm.

Owing to a paucity of head-to-head RCTs comparing the harms associated with pharmacologic interventions in persons with dementia, we used NMA to fill a critical knowledge gap. For example, we used indirect evidence to demonstrate that antipsychotics are associated with increased odds of death compared to antidepressants in older adults with dementia. Knowledge users (e.g. persons with dementia, caregivers, and clinicians) can personalize decision-making by using our rank-heat plot to visualize treatment risk rankings across each of the four outcomes of harm (Fig. 3).

Similar to two published NMAs of pharmacologic interventions in persons with dementia, we found that antipsychotics were associated with an increased risk of cerebrovascular events [30, 31]. Although one of these NMAs included cholinesterase inhibitors, authors did not conduct analyses to assess for an association with mortality risk [30]. We found a possible association between cholinesterase inhibitor use and lower mortality risk among RCTs only; however, wide predictive intervals (that cross one) associated with this effect estimate suggest that, in a future study, we might not see this association. The strength of this finding may be limited by: the greater uncertainty in our Bayesian compared to frequentist effect estimate, the dependence of our effect estimate on unpublished data, and a number of studies at high risk of bias from missing data [32].

There were limitations to our study. First, although neuropsychiatric symptoms occur in 75% of patients with dementia each month, only 42.6% of included studies reported a measure of neuropsychiatric symptom severity (e.g. Neuropsychiatric Inventory) [33]. Therefore, our systematic review included all studies of persons with dementia who received pharmacologic interventions commonly used for neuropsychiatric symptoms. Second, as a significant amount of adverse event data were not reported in primary RCTs, we abstracted these data from clinical trial registries, systematic reviews, meta-analyses, and secondary analyses of RCTs. Third, we were not able to conduct missing data analyses to test the missing at random assumption because these data were not always available (see Additional file 1 for protocol deviations). Lastly, we did not derive effect estimates for specific treatments (e.g. risperidone, olanzapine) or doses because outcome data were too sparsely reported across treatment networks.

## Conclusion

In conclusion, we demonstrated potential harms associated with pharmacologic interventions intended to treat neuropsychiatric symptoms in persons with dementia. Our findings suggest that changing prescribing trends towards pharmacologic interventions other than antipsychotics may not be appropriate given the risk for harm. Rather, we should advocate for increased use of nonpharmacologic interventions given their demonstrated efficacy [10].

## Supplementary information

**Additional file 1.** Appendix.

## Data Availability

The full RCT dataset is available in Additional file 1 Table 3.

## Abbreviations

CI: Confidence interval
CrI: Credible interval
HR: Hazard ratio
NMDA: N-methyl-D-aspartate
NMA: Network meta-analysis
NRS: Non-randomized study
NNH: Number needed to harm
OR: Odds ratio
PrI: Predictive interval
RCT: Randomized trial
RR: Relative risk
SUCRA: Surface under the cumulative ranking curve
