# Supplementary material for: Safety of pharmacologic interventions for neuropsychiatric symptoms in dementia: a systematic review and network meta-analysis
Source: BMC Geriatr. 2020 Jun 16;20:212. doi: 10.1186/s12877-020-01607-7 (PMC7298771; doi:10.1186/s12877-020-01607-7)
Supplement: Supplementary file 1 — Additional file 1. Appendix. [file 12877_2020_1607_MOESM1_ESM.docx]

Contents: Appendix

[File 1. Protocol Deviations 3](#_Toc25913464)

[File 2. Grey Literature Search Strategy Databases and Websites 4](#_Toc25913465)

[Table 1. Individual Study Characteristics: Randomized and Non-Randomized Studies 5](#_Toc25913466)

[Table 2. Individual Study-Level Patient Characteristics: Randomized and Non-Randomized Studies 15](#_Toc25913467)

[Table 3. Arm-Level Data for RCTs Included in Pairwise and Network Meta-Analyses 23](#_Toc25913468)

[Table 4a. Assessment of Transitivity: RCTs Reporting Fractures 27](#_Toc25913469)

[Table 4b. Assessment of Transitivity: RCTs Reporting Mortality 28](#_Toc25913470)

[Table 4c. Assessment of Transitivity: RCTs Reporting Stroke 29](#_Toc25913471)

[Table 4d. Assessment of Transitivity: RCTs Reporting Falls 30](#_Toc25913472)

[Table 5. Risk of Bias Assessment for Each RCT with the Cochrane Risk of Bias Tool[219] 31](#_Toc25913473)

[Table 6. Risk of Bias Assessment for Each Cohort Study with the Newcastle-Ottawa Quality Assessment Scale[220] 36](#_Toc25913474)

[Table 7. Risk of Bias Assessment for Each Case-Control Study with the Newcastle-Ottawa Quality Assessment Scale [220] 38](#_Toc25913475)

[Table 8. Risk of Bias Assessment for Each ‘Other’ Non-Randomized Study with the Cochrane Effective Practice and Organization of Care Tool [221] 38](#_Toc25913476)

[Figure 1. Summary of Risk of Bias Assessment for RCTs 39](#_Toc25913477)

[Table 9a. Bayesian Pairwise and Network Meta-Analysis: RCTs Reporting Fractures 40](#_Toc25913478)

[Table 9b. Frequentist Pairwise and Network Meta-Analysis: RCTs Reporting Fractures 41](#_Toc25913479)

[Table 9c. Bayesian Network Meta-Analysis: RCTs + NRSs Reporting Fractures 42](#_Toc25913480)

[Table 9d. Bayesian Pairwise and Network Meta-Analysis: RCTs Reporting Mortality 43](#_Toc25913481)

[Table 9e. Frequentist Pairwise and Network Meta-Analysis: RCTs Reporting Mortality 44](#_Toc25913482)

[Table 9f. Bayesian Network Meta-Analysis: RCTs + NRSs Reporting Mortality 45](#_Toc25913483)

[Table 9g. Bayesian Pairwise and Network Meta-Analysis: RCTs Reporting Cerebrovascular Event 46](#_Toc25913484)

[Table 9h. Frequentist Pairwise and Network Meta-Analysis: RCTs Reporting Cerebrovascular Event 47](#_Toc25913485)

[Table 9i. Bayesian Network Meta-Analysis: RCTs + NRSs Reporting Cerebrovascular Event 48](#_Toc25913486)

[Table 9j. Bayesian Pairwise and Network Meta-Analysis: RCTs Reporting Falls 49](#_Toc25913487)

[Table 9k. Frequentist Pairwise and Network Meta-Analysis: RCTs Reporting Falls 50](#_Toc25913488)

[Table 9l. Bayesian Network Meta-Analysis: RCTs + NRSs Reporting Falls 51](#_Toc25913489)

[Figure 2a. Comparison-Adjusted Funnel Plot: RCTs Reporting Fracture 52](#_Toc25913490)

[Figure 2b. Comparison-Adjusted Funnel Plot: RCTs + NRSs Reporting Fracture 52](#_Toc25913491)

[Figure 2c. Comparison-Adjusted Funnel Plot: RCTs Reporting Mortality 53](#_Toc25913492)

[Figure 2d. Comparison-Adjusted Funnel Plot: RCTs + NRSs Reporting Mortality 53](#_Toc25913493)

[Figure 2e. Comparison-Adjusted Funnel Plot: RCTs Reporting Cerebrovascular Event 54](#_Toc25913494)

[Figure 2f. Comparison-Adjusted Funnel Plot: RCTs + NRSs Reporting Cerebrovascular Event 54](#_Toc25913495)

[Figure 2g. Comparison-Adjusted Funnel Plot: RCTs Reporting Falls 55](#_Toc25913496)

[Figure 2h. Comparison-Adjusted Funnel Plot: RCTs + NRSs Reporting Falls 55](#_Toc25913497)

[Figure 3a. Inconsistency Plot: RCTs Reporting Fracture 56](#_Toc25913498)

[Figure 3b. Inconsistency Plot: RCTs + NRSs Reporting Fracture 56](#_Toc25913499)

[Figure 3c. Inconsistency Plot: RCTs Reporting Mortality 57](#_Toc25913500)

[Figure 3d. Inconsistency Plot: RCTs + NRSs Reporting Mortality 57](#_Toc25913501)

[Figure 3e. Inconsistency Plot: RCTs Reporting Cerebrovascular Event 58](#_Toc25913502)

[Figure 3f. Inconsistency Plot: RCTs + NRSs Reporting Cerebrovascular Event 58](#_Toc25913503)

[Figure 3g. Inconsistency Plot: RCTs Reporting Falls 59](#_Toc25913504)

[Figure 3h. Inconsistency Plot: RCTs + NRSs Reporting Falls 59](#_Toc25913505)

[References 60](#_Toc25913506)

# File 1. Protocol Deviations

We made the following changes to our study protocol:[1]

1. Our primary analyses were based on data from randomized controlled trials (RCTs) only. In our protocol, we decided that our summary effect estimate should be an odds ratio (OR). Many of the non-randomized studies reported rate ratios. Therefore, we decided our primary analyses would include RCT data and we conducted a secondary analysis where we assumed that a rate ratio was approximately equivalent to relative risk, which allowed us to combine data from randomized and non-randomized studies.
2. To lessen uncertainty in our effect estimates, we used the informative priors derived by Turner et al. for death and semi-objective outcomes (i.e. stroke, fall, and fracture) for our common within-network between-study heterogeneity parameters in our primary analyses.[2] We tested the effect of using a weakly informative prior in place of the informative prior as the heterogeneity parameter in a sensitivity analysis.
3. We did not perform analyses to test for the impact of missing data on our NMA effect estimates. Data from a number of RCTs was not published in primary studies reported in scholarly journals, but it was found in secondary analyses, meta-analyses, or clinical trial registries. Many of these studies would be excluded from missing data analyses, which would render said analyses uninterpretable.
4. In secondary analyses, we conducted frequentist random-effects network and pairwise meta-analyses for each outcome. Frequentist approaches are more commonly reported in the medical literature and we wanted to facilitate interpretation of our findings.[3]

# File 2. Grey Literature Search Strategy Databases and Websites

**General grey literature**

Government of Canada:  <http://publications.gc.ca/site/eng/search/eCollection.html>

GreyNet International:  [http://www.greylit.org](http://www.greylit.org/)

SIGLE (System for Information on Grey Literature in Europe):  [http://www.opengrey.eu](http://www.opengrey.eu/)

**Search engines**

TRIP database:  <http://www.tripdatabase.com/>

Google: <https://www.google.ca/advanced_search>

Google Scholar:  <https://scholar.google.com/intl/en/scholar/about.html>

**International**

Agency for Healthcare Research and Quality (see for policy makers): <http://www.ahrq.gov/research/index.html>

WHO (WHOLIS): <http://dosei.who.int/uhtbin/cgisirsi/Tue+Apr++5+17:45:43+MEST+2016/0/49>

**Thesis**

Center for Research Libraries Foreign Dissertation: <https://www.crl.edu/collections/topics/dissertations>

DART-Europe E-theses Portal: <http://www.dart-europe.eu/basic-search.php>

Electronic Theses Online Service (ETHOS) | British Library:  <http://ethos.bl.uk/Home.do;jsessionid=D96E9CF245B0FE0199DDDB94FF4BD2A7>

Open access dissertations: [https://oatd.org](https://oatd.org/)

Thesis Canada Portal:  <http://www.bac-lac.gc.ca/eng/services/theses/Pages/theses-canada.aspx>

# Table 1. Individual Study Characteristics: Randomized and Non-Randomized Studies

| **Author, Year** | **Study Design** | **Setting** | **Interventions** | **Study Length (weeks)** | **Outcome(s) Reported** | **Name of Study Sponsorship Organization** |
| --- | --- | --- | --- | --- | --- | --- |
| 5077 US-039[4] | Parallel RCT | LTC | Quetiapine, placebo | 10 | Mortality | - |
| Aarsland, 2009[5] | Parallel RCT | Specialty Clinic | Memantine, placebo | 24 | Stroke | H Lundbeck A/S, Western Norway Regional Health Authority |
| AD2000 Collaborative Group, 2004[6] | Cross-Over RCT | Home/  Community | Donepezil, placebo | 192 | Mortality | NHS Executive R&D (West Midlands); Eisai; Pfizer |
| Aguglia, 2004[7] | Cohort Study | Alzheimer Evaluation Unit | Rivastigmine, galantamine, donepezil | 26 | Mortality | Novartis Pharma AG |
| Allain, 2000[8] | Parallel RCT | LTC/Hospital | Haloperidol, placebo | 3 | Mortality | - |
| Ancoli-Israel, 2005[9] | Parallel RCT | Home/  Community | Galantamine, donepezil | 8 | Mortality | Janssen Medical Affairs, L.L.C |
| Auchus, 2007[10] | Parallel RCT | - | Galantamine, placebo | 26 | Mortality, stroke, falls | - |
| B351[11] | Parallel RCT | - | Rivastigmine, placebo | 26 | Fracture | Novartis |
| Bakchine, 2007[12] | Parallel RCT | Clinic | Memantine, placebo | 24 | Mortality, stroke, falls | H. Lundbeck A/S |
| Ballard, 2004[13] | Parallel RCT | LTC | Antipsychotics, placebo | 13 | Mortality | Research into Ageing, Age Concern |
| Ballard, 2005[14] | Parallel RCT | Care facility | Quetiapine, rivastigmine, placebo | 26 | Mortality | Alzheimer's Research Trust |
| Ballard, 2008[15] | Parallel RCT | LTC/Assisted Living | Antipsychotics, placebo | 52 | Mortality | The Alzheimer's Research Trust |
| Ballard, 2008[15] | Parallel RCT | - | Rivastigmine, placebo | 24 | Mortality, stroke, falls | Novartis |
| Ballard, 2015[16] | Parallel RCT | LTC | Memantine, antipsychotics | 24 | Mortality, stroke | Lundbeck |
| Ballard, 2018[17] | Parallel RCT | LTC | Pimavanserin, placebo | 12 | Mortality, stroke, fracture, falls | ACADIA Pharmaceuticals |
| Banerjee, 2011[18] | Parallel RCT | Specialty clinic/LTC/home | Sertraline, mirtazapine, placebo | 13 | Mortality | UK National Institute for Health Research (NIHR) Health Technology Assessment (HTA) programme |
| Barak, 2011[19] | Parallel RCT | Hospital | Risperidone, escitalopram | 6 | Falls | H. Lundbeck A/S |
| Barnett, 2007[20] | Cohort Study | - | Haloperidol, quetiapine, olanzapine, risperidone | 86 | Stroke | Department of Veterans Affairs, the Veterans Health Administration, Health Services Research and Development Service; Eli Lilly and Company |
| Baxer, 2013[21] | Parallel RCT | Specialty Clinic | Memantine, placebo | 26 | Falls | Forest Research Institute |
| Black, 2003[22] | Parallel RCT | Outpatient | Donepezil, placebo | 24 | Mortality, stroke | Eisai Inc. |
| Black, 2007[23] | Parallel RCT | Clinic | Donepezil, placebo | 24 | Mortality | - |
| Brodaty, 2003[24] | Parallel RCT | - | Risperidone, placebo | 12 | Mortality, stroke, falls | Janssen-Cilag Australi, Johnson & Johnson, L.L.C. |
| Brodaty, 2005[25] | Parallel RCT | - | Galantamine, placebo | 26 | Mortality, falls | - |
| Bronskill, 2018[26] | Cohort Study | LTC | Trazodone, benzodiazepines | 13 | Fracture, falls | Canadian Frailty Network |
| Bullock, 2005[27] | Parallel RCT | Outpatient | Rivastigmine, donepezil | 104 | Mortality, falls | Novartis Pharma AG, Basel, Switzerland |
| Burns, 1999[28] | Parallel RCT | - | Donepezil, placebo | 24 | Mortality | Eisai Inc., Teaneck, N.J., USA; Eisai Co. Ltd., Tokyo, Japan |
| Burns, 2009[29] | Parallel RCT | LTC | Galantamine, placebo | 26 | Mortality, falls | Janssen-Cilag EMEA |
| Camargos, 2014[30] | Parallel RCT | Specialty Clinic | Trazodone, placebo | 2 | Fracture | No funding |
| Campbell, 2017[31] | Parallel RCT | Specialty Clinic | Donepezil, galantamine, rivastigmine | 18 | Falls | Agency for Healthcare Research and Quality (AHRQ) |
| Carlyle, 1993[32] | Parallel RCT | Specialty Clinic | Haloperidol, loxapine | 4 | Falls | - |
| Chan, 2001[33] | Parallel RCT | Specialty Inpt/Outpt Services | Haloperidol, risperidone | 12 | Fracture | Janssen Research Foundation |
| Chan, 2010[34] | Cohort Study | Hospital | Haloperidol, usual care | 146* | Stroke | - |
| Choe, 2016[35] | Parallel RCT | Specialty Clinic | Escitalopram, placebo | 52 | Mortality, stroke, falls | H. Lundbeck A/S (Copenhagen, Denmark), Ministry of Science, ICT, and Future Planning, Republic of Korea |
| Choi, 2011[36] | Parallel RCT | - | Memantine + rivastigmine patch, rivastigmine patch | 16 | Fracture | Novartis; Korea Healthcare technology R&D project; Ministry for Health & Welfare |
| Corey-Bloom, 1998[37] | Parallel RCT | - | Rivastigmine, placebo | 26 | Mortality, fracture | Novartis Pharmaceuticals |
| Culo, 2010[38] | Parallel RCT | Specialty Clinic/Hospital | Citalopram, risperidone | 12 | Mortality | USPHS; Sandra A. Rotman Program in Neuropsychiatry (Toronto) |
| Cumbo, 2014[39] | Parallel RCT | Clinic | Memantine, donepezil, galantamine, rivastigmine | 52 | Mortality | Alzheimer and Dementia Unit of the Neurodegenerative disorder O.U. of the NHS District of Caltanissetta |
| Cummings, 2012[40] | Parallel RCT | Home/Community | Rivastigmine patch 15cm, rivastigmine patch 10cm | 48 | Mortality, fracture, falls | Novartis |
| Cummings, 2015[41] | Parallel RCT | Specialty clinic/AL/LTC | Dextromethorphan-quinidine, placebo | 10 | Mortality, stroke, falls | Avanir Pharmaceutical Inc. |
| De Deyn, 1999[42] | Parallel RCT | LTC | Risperidone, placebo | 12 | Mortality, stroke | Janssen Research Foundation, Beerse, Belgium. |
| De Deyn, 2004[43] | Parallel RCT | LTC | Olanzapine, placebo | 10 | Mortality, stroke | Eli Lilly and Company |
| De Deyn, 2005[44] | Parallel RCT | Home/Community/Assisted Living | Aripiprazole, placebo | 10 | Mortality, stroke, fracture, falls | Bristol-Myers Squibb, Otsuka Pharmaceutical Co., Ltd. |
| de Vasconcelos Cunha, 2007[45] | Parallel RCT | Outpatient | Venlafaxine, placebo | 6 | Falls | - |
| Deberdt, 2005[46] | Parallel RCT | LTC/AL/Clinic | Risperidone, olanzapine, placebo | 10 | Mortality, stroke, falls | Eli Lilly and Company |
| Devanand, 2012[47] | Parallel RCT | Community/LTC/Assisted Living | Risperidone, placebo | 32 | Mortality, fracture, falls | National Institutes of Health; Department of Veterans Affairs |
| Donepezil 319[48] | Parallel RCT | - | Donepezil, placebo | 24 | Fracture | Eisai |
| Doody, 2012[49] | Parallel RCT | - | Donepezil + memantine, donepezil | 24 | Mortality, falls | Pfizer |
| Dubois, 2012[50] | Parallel RCT | Clinic | Donepezil, placebo | 24 | Mortality, fracture, falls | Eisai Inc. |
| Dysken, 2014[51] | Parallel RCT | Veterans Affairs Medical Centres | Memantine, placebo | 118* | Mortality, stroke, fracture, falls | Veterans Affairs Cooperative Studies Program, Office of Research and Development, Clinical Science R&D. |
| Eady, 2018[52] | Cohort Study | Community | Cholinesterase inhibitor + memantine, cholinesterase inhibitor, memantine, usual care | NR | Mortality | Baily Thomas Charitable Fund |
| Emre, 2004[53] | Parallel RCT | - | Rivastigmine, placebo | 24 | Mortality, falls | Novartis |
| Emre, 2010[54] | Parallel RCT | Specialty Clinic | Memantine, placebo | 24 | Mortality, stroke, falls | Lundbeck |
| Emre, 2014[55] | Parallel RCT | Home/Community | Rivastigmine, rivastigmine patch | 76 | Mortality, stroke, fracture, falls | Novartis Pharma AG, Basel, Switzerland |
| Erkinjuntti, 2002[56] | Parallel RCT | - | Galantamine, placebo | 26 | Mortality, stroke | Janssen Research Foundation |
| Farlow, 2010[57] | Parallel RCT | - | Rivastigmine + memantine, rivastigmine | 5 | Mortality, stroke, fracture, falls | - |
| Farlow, 2013[58] | Parallel RCT | - | Rivastigmine patch 13.3mg, rivastigmine patch 4.6mg | 24 | Mortality, stroke, fracture, falls | Novartis Pharmaceuticals Corporation |
| Feldman, 2001[59] | Parallel RCT | Home/Community/Assisted Living | Donepezil, placebo | 24 | Mortality | Pfizer, Inc. (New York, NY), Eisai, Inc. (Teaneck, NJ) |
| Feldman, 2007[60] | Parallel RCT | - | Rivastigmine, placebo | 26 | Mortality, fracture | Novartis Pharma AG |
| Finkel, 1995[61] | Parallel RCT | LTC | Thiothixene, placebo | 11 | Mortality | - |
| Finkel, 2005[62] | Cohort Study | - | Benzodiazepines, antipsychotics | 13 | Stroke | Ortho-McNeil Janssen Scientific Affairs, LLC |
| Fontaine, 2003[63] | Parallel RCT | LTC/Assisted Living | Risperidone, olanzapine | 2 | Mortality, stroke, falls | Eli Lilly and Company, Indianapolis, Ind |
| Fox, 2012[64] | Parallel RCT | LTC/hospital | Memantine, placebo | 12 | Mortality | Lundbeck |
| GAL-95-05[65, 66] | Parallel RCT | - | Galantamine, placebo | NR | Mortality | - |
| GAL-JPN-3[65] | Parallel RCT | - | Galantamine, placebo | NR | Mortality | - |
| GAL-USA-16[65] | Parallel RCT | - | Galantamine, placebo | NR | Mortality | - |
| Gasper, 2005[67] | Cohort Study | LTC | Donepezil, usual care | 104 | Mortality | US Department of Health and Human Services, Health Care and Financing Administration to the University of Michigan, NIA |
| Gault, 2016[68] | Parallel RCT | - | Donepezil, placebo | 24 | Mortality, falls | AbbVie Inc |
| Gerhard, 2014[69] | Cohort Study | Home/Community | Aripirazole, haloperidol, olanzapine, quetiapine, ziprasidone | 26 | Mortality | Agency for Healthcare Research and Quality/Food and Drug Administration (AHRQ/FDA); AHRQ |
| Gill, 2005[70] | Cohort Study | - | Typical antipsychotics, atypical antipsychotics | 260 | Stroke | Health Research Chronic Disease New Emerging Team |
| Gill, 2007[71] | Cohort Study | Community/LTC | Atypical antipsychotics, usual care | 26 | Mortality | Canadian Institutes for Health Research; Canadian Diabetes Association, the Kidney Foundation of Canada, the Heart and Stroke Foundation of Canada and the Canadian Institutes for Health Research Institutes of Nutrition, Metabolism & Diabetes and Circulatory & Respiratory Health |
| Gill, 2009[72] | Cohort Study | Community | Cholinesterase inhibitors, control | 104 | Fracture | Clinical Teachers Association of Queen’s Endowment Fund, Chronic Disease New Emerging Team Program, Canadian Institutes of Health Research |
| Gold, 2010[73] | Parallel RCT | - | Donepezil, placebo | 24 | Mortality, stroke, fracture | GlaxoSmithKline |
| Grossberg, 2013[74] | Parallel RCT | Clinic | Memantine, placebo | 24 | Mortality, stroke, fracture, falls | Forest Laboratories, Inc. |
| Hager, 2014[75] | Parallel RCT | - | Galantamine, placebo | 104 | Mortality, stroke, fracture, falls | Janssen Research and Development LLC, Raritan, New Jersey, USA. |
| Hampel, 2009[76] | Parallel RCT | Specialty Clinic | Lithium, placebo | 10 | Mortality | Astra Zeneca, Adelaide and Meath Hospital |
| Herrmann, 2007[77] | Cross-Over RCT | LTC | Valproate, placebo | 12 | Falls | Alzheimer’s Society of Canada |
| Herrmann, 2013[78] | Parallel RCT | - | Memantine, placebo | 24 | Mortality, stroke, fracture, falls | H. Lundbeck A/S |
| Herrmann, 2016[79] | Parallel RCT | LTC | Cholinesterase inhibitors, placebo | 8 | Mortality, falls | Alzheimer’s Society of Canada, Coleman Fund |
| HGAO[4] | Parallel RCT | Outpatient | Olanzapine, placebo | 8 | Mortality, stroke | Eli Lilly and Company |
| Homma, 1998[80] | Parallel RCT | 55 facilities | Donepezil, placebo | 12 | Mortality | - |
| Homma, 2000[81] | Parallel RCT | Outpatient | Donepezil, placebo | 24 | Fracture | - |
| Homma, 2008[82] | Parallel RCT | Home/Community/Assisted Living | Donepezil, placebo | 24 | Mortality, falls | Eisai Co., Ltd., Tokyo, Japan |
| Howard, 2007[83] | Parallel RCT | Home/Residential care facility/other | Donepezil, placebo | 12 | Mortality, stroke, fracture, falls | Eisai UK, MRC, Alzheimer's Society |
| Howard, 2012[84] | Parallel RCT | Clinic | Donepezil + memantine, memantine, donepezil, placebo | 52 | Mortality, stroke, falls | U.K. Medical Research Council and the U.K. Alzheimer’s Society |
| Hu, 2006[85] | Parallel RCT | Outpatient | Memantine, donepezil | 16 | Mortality | - |
| Huybrechts, 2012[86] | Cohort Study | LTC | Haloperidol, risperidone, aripiprazole, olanzapine, quetiapine, ziprasidone | 26 | Mortality | AHRQ/FDA; National Institute of Mental Health |
| Jalbert, 2010[87] | Case-Control Study | LTC | Atypical antipsychotics, usual care | NR | Fracture | Pfizer |
| Jia, 2017[88] | Parallel RCT | - | Donepezil, placebo | 24 | Mortality, stroke | - |
| Johannsen, 2006[89] | Parallel RCT | Home/community/assisted living | Donepezil, placebo | 12 | Mortality | Pfizer Inc., Eisai Inc. |
| Kales, 2007[90] | Cohort Study | - | SSRIs, TCAs, anticonvulsants, anxiolytic/hypnotics, antipsychotics, usual care | 52 | Mortality | VA Health Services Research; Career Development Award; VA grant |
| Kales, 2012[91] | Cohort Study | Specialty Clinic | Valproic acid and derivatives, risperidone | 26 | Mortality | NIMH |
| Katona, 1998[92] | Parallel RCT | Home/Community | Imipramine, paroxetine | 8 | Mortality | SmithKline Beecham Pharmaceuticals |
| Katz, 1999[93]^ | Parallel RCT | LTC | Risperidone, placebo | 12 | Mortality, falls | Janssen Research Foundation, |
| Katz, 2004[94]^ | Parallel RCT | LTC | Risperidone, placebo | 12 | Fracture | Janssen Pharmaceutica Products, L.P. |
| Kennedy, 2005[95] | Parallel RCT | Home/Community/Assisted Living | Olanzapine, placebo | 26 | Mortality, stroke | Eli Lilly and company |
| Kertesz, 2008[96] | Parallel RCT | Specialty Clinic | Galantamine, placebo | 8 | Mortality | Janssen-Ortho Inc., Canad, and Johnson & Johnson Pharmaceutical Research and Development. |
| Kheirbek, 2019[97] | Cohort Study | Community | Antipsychotics, usual care | NR | Mortality | Veterans Affairs Capitol Health Care Network; National Center for Advancing Translational Science of the National Institutes of Health |
| Lanctot, 2002[98] | Cross-Over RCT | LTC | Sertraline, placebo | 8 | Falls | Pfizer, Physicians' Services Incorporated Foundation, Alzheimer Society of Canada, Kunin-Lunenfeld Applied Research Unit |
| Langballe, 2014[99] | Cohort Study | Outpatient | Risperidone, haloperidol | 343 | Mortality | Research Council of Norway, National Institute for Health Research (UK), Biomedical Research Unit for Dementia and the Biomedical Research Centre for Mental Health at King’s College London |
| Layton, 2005[100] | Cohort Study | - | Risperidone, quetiapine, olanzapine | 26 | Stroke | Drug Safety Research Unit |
| Lee, 2017[101] | Cohort Study | - | Cholinesterase inhibitors, rivastigmine patch | 24 | Mortality, falls | Novartis |
| Lin, 2016[102] | Cohort Study | - | Cholinesterase inhibitors, usual care | 263* | Mortality, stroke | Kaohsiung Medical University Hospital |
| Liperoti, 2005[103] | Case-Control Study | LTC | Risperidone, olanzapine, typical antipsychotics, usual care | NR | Stroke | National Institute on Aging, National Institutes of Health |
| Liperoti, 2009[104] | Cohort Study | LTC | Typical antipsychotics, atypical antipsychotics | 26 | Mortality | National Institute on Aging, National Institutes of Health |
| Litvinenko, 2008[105] | Parallel RCT | Specialty Clinic | Galantamine, usual care | 24 | Falls | - |
| Lopez, 1999[106] | Cohort Study | Specialty Clinic | Antidepressants, antipsychotics, sedative/hypnotics, usual care | 215* | Mortality | National Institute on Aging, Bethesda, MD |
| Lopez, 2009[107] | Cohort Study | Specialty Clinic | Cholinesterase inhibitor + memantine, memantine, usual care | 271* | Mortality | National Institute on Aging, VISN 4 Mental Illness Research Education and Clinical Center, VA Pittsburgh Health Care System, Pittsburgh, Pennsylvania. |
| Lopez-Pousa, 2006[108] | Cohort Study | Specialty Clinic | Donepezil, galantamine | 101* | Mortality | - |
| Lyketsos, 2003[109] | Parallel RCT | Home/Community/Assisted Living | Sertraline, placebo | 12 | Mortality, falls | National Institute of Mental Health, Bethseda, MD |
| Maher-Edwards, 2011[110] | Parallel RCT | Clinic | Donepezil, placebo | 24 | Mortality | GlaxoSmithKline |
| Martin, 2003[111] | Cohort Study | LTC | Risperidone, olanzapine | 13 | Falls | Janssen Pharmaceutica Products, L.P. |
| Martinez Martinez, 2009[112] | Cohort Study | - | Quetiapine, ziprasidone, olanzapine | 122 | Mortality | - |
| Maust, 2015[113] | Case-Control Study | Specialty Clinic | Haloperidol, valproic acid, usual care | 26 | Mortality | National Institute on Aging,  American Federation for Aging Research, The John A. Hartford Foundation, The Atlantic Philanthropies |
| McKeith, 2000[114] | Parallel RCT | Specialty Clinic | Rivastigmine, placebo | 20 | Mortality | Novartis |
| Mintzer, 2006[115] | Parallel RCT | LTC | Risperidone, placebo | 8 | Mortality, stroke, falls | Johnson & Johnson Pharmaceutical Research & Development, L.L.C. |
| Mintzer, 2007[116] | Parallel RCT | LTC | Aripiprazole, placebo | 10 | Mortality, stroke | Bristol-Myers Squibb Company and Otsuka Pharmaceutical Development & Commercialization (Princeton, NJ) |
| Mohs, 2001[117] | Parallel RCT | - | Donepezil, placebo | 54 | Mortality | Eisai, Inc. and Pfizer, Inc. |
| Mok, 2007[118] | Parallel RCT | Specialty Clinic | Rivastigmine, placebo | 26 | Mortality, stroke | Novartis Pharmaceutical (Hong Kong), Ltd. |
| Moretti, 2005[119] | Parallel RCT | LTC | Typical antipsychotics, olanzapine | 52 | Mortality, fracture, falls | - |
| Mueller, 2017[120] | Cohort Study | - | Cholinesterase inhibitors, usual care | 191* | Mortality | National Institute for Health Research Mental Health Biomedical Research Centre at South London; Maudsley NHS Foundation Trust and King’s College London; Guy’s and St Thomas’ Charity, Maudsley Charity |
| Musicco, 2011[121] | Cohort Study | - | Antipsychotics, usual care | 103 | Mortality | European Research Council |
| Nakamura, 2011[122] | Parallel RCT | - | Rivastigmine patch, placebo | 24 | Mortality, stroke, fracture, falls | Novartis and Ono Pharmaceutical Co. Ltd. |
| Nakamura, 2014[123] | Pairwise Meta-Analysisf | Clinic | Memantine, placebo | 24 | Falls | Daiichi Sankyo Co. |
| Nordstrom, 2013[124] | Cohort Study | LTC/community | Cholinesterase inhibitors, memantine, usual care | 72* | Mortality | Swedish research council, Swedish Brain Power consortium, Swedish Society of Mediine and Foundation for Geriatric Diseases at Karolinska Institutet, Swedish Dementia Foundation, Swedish Association of Local Authorities and Regions |
| Nyth, 1990[125] | Parallel RCT | Hospital | Citalopram, placebo | 16 | Mortality | - |
| Orgogozo, 2002[126] | Parallel RCT | - | Memantine, placebo | 28 | Mortality | Merz Pharmaceuticals, Frankfurt/M., Germany |
| Pakdaman, 2015[127] | Parallel RCT | - | Donepezil, galantamine, rivastigmine | 70 | Mortality | - |
| Paleacu, 2008[128] | Parallel RCT | - | Quetiapine, placebo | 6 | Falls | AstraZeneca |
| Peskind, 2006[129] | Parallel RCT | Outpatient | Memantine, placebo | 24 | Mortality, falls | Forest Laboratories, Inc. |
| Peters, 2015[130] | Parallel RCT | - | Memantine + galantamine, galantamine | 52 | Falls | Bundesministerium fur Bildung und Forschung |
| Piersanti, 2014[131] | Cohort Study | Specialty Clinic | Atypical antipsychotics, usual care | 156 | Mortality | - |
| Porsteinsson, 2001[132] | Parallel RCT | LTC | Divalproex sodium, placebo | 6 | Stroke, falls | Alzheimer's Association, National Institute on Aging, Abbott Laboratories |
| Porsteinsson, 2008[133] | Parallel RCT | Home/Community | Cholinesterase inhibitors + memantine, cholinesterase inhibitor | 24 | Mortality, falls | Forest Laboratories, Inc., New York, NY |
| Porsteinsson, 2014[134] | Parallel RCT | Clinic/LTC/Community | Citalopram, placebo (+psychosocial interention) | 9 | Mortality | NIA, NIMH |
| Profenno, 2005[135] | Parallel RCT | Home/Community | Divalproex, placebo | 10 | Falls | Abbott Laboratories, NIA |
| Rafaniello, 2014[136] | Cohort Study | Dementia Unit | Quetiapine, risperidone, olanzapine, clozapine, aripiprazole | 44* | Mortality | - |
| Rainer, 2007[137] | Parallel RCT | Outpatient | Quetiapine, risperidone | 8 | Mortality, stroke, fracture, falls | AstraZeneca Pharmaceuticals. |
| Raivio, 2007[138] | Cohort Study | Hospital/LTC | Typical antipsychotics, atypical antipsychotics, usual care | 104 | Mortality | Societas Gerontologica Fennica, Finnish Geriatric Association, Uuolo Arhio Foundation, Medical Society of Kyminlaakso, Duodecim, Finland |
| Rappaport, 2009[139] | Parallel RCT | Health care facility | Aripiprazole, placebo | 0 | Mortality, stroke, fracture, falls | Bristol-Myers Squibb, Otsuka Pharmaceutical Co., Ltd. |
| Raskind, 2000[140] | Parallel RCT | - | Galantamine, placebo | 26 | Mortality | Janssen Research Foundation |
| Raskind, 2000; Wilcock, 2000[48] | Parallel RCT | Clinic | Galantamine, placebo | 26 | Falls | - |
| Reisberg, 2003[141] | Parallel RCT | Home/Community | Memantine, placebo | 28 | Mortality, falls | Merz Pharmaceuticals |
| RIS-BEL-14[142, 143] | Parallel RCT | LTC/Assisted Living | Risperidone, placebo | 4 | Mortality, stroke | Johnson & Johnson Pharmaceutical Research and Development |
| RIS-INT-83[142, 144] | Parallel RCT | LTC/Assisted Living | Risperidone, placebo | 8 | Mortality, stroke | Johnson & Johnson Pharmaceutical Research and Development |
| Rochon, 2008[145] | Cohort Study | Home/Community/  LTC | Atypical antipsychotics, usual care | 4 | Mortality, stroke | Canadian Institutes of Health Research (CIHR) |
| Rockwood, 2001[146] | Parallel RCT | - | Galantamine, placebo | 13 | Mortality | Janssen Research Foundation, Beerse, Belgium |
| Rogers, 1998a[147] | Parallel RCT | Outpatient | Donepezil, placebo | 12 | Mortality, stroke | Eisai, Inc. Tenaeck, NJ; Eisai Co Ltd. Tokyo, Japan |
| Rogers, 1998b[148] | Parallel RCT | - | Donepezil, placebo | 24 | Mortality, stroke, fracture | NIA, Howard Hughes Institute, Fulbright Commission, United States Information Agency, Human Brain Project, NCRR, Alzheimer’s Disease Center, NIMH, Alzheimer’s Association |
| Roman, 2010[149] | Parallel RCT | Outpatient | Donepezil, placebo | 24 | Mortality | Eisai Medical Research Inc |
| Rosenberg, 2010[150] | Parallel RCT | Home/Community | Sertraline, placebo | 12 | Mortality, falls | National Institute of Mental Health |
| Rosenberg, 2013[151] | Parallel RCT | Medical Centre | Methylphenidate, placebo | 6 | Mortality | National Institute on Aging |
| Rosler, 1999[152] | Parallel RCT | Home/Community | Rivastigmine, placebo | 26 | Mortality, fracture | Novartis Pharma AG, Basle, Switzerland |
| Rossom, 2010[153] | Cohort Study | - | Haloperidol, usual care | 4 | Mortality | Veterans Health Administration University of Minnesota; Minnesota Veterans Research Institute |
| Ruths, 2008[154] | Parallel RCT | LTC | Antipsychotics, placebo | 4 | Mortality | - |
| Sahlberg, 2015[155] | Cohort Study | - | Risperidone, flupentixol, chlorprothixen, levomepromazine, haloperidol, ziprazidone, quetiapine, olanzapine | >52.1429 | Mortality | Danish Agency for Science, Technology and Innovation; Novo Nordisk Foundation |
| Santos-Garcia, 2010[156] | Cohort Study | NR | Quetiapine, ziprasidone, olanzapine | 87* | Mortality, stroke | - |
| Saxton, 2012[157] | Parallel RCT | Clinic | Memantine, placebo | 12 | Mortality, stroke, fracture, falls | Forest Laboratories, Inc. |
| Scarpini, 2011[158] | Parallel RCT | - | Galantamine, placebo | 104 | Mortality | Janssen-Cilag EMEA |
| Schneeweiss, 2007[159] | Cohort Study | LTC/community | Typical antipsychotics, atypical antipsychotics | 26 | Mortality | NIA, Agency for Health Care Quality and Research |
| Schneider, 2006[160] | Parallel RCT | Home/Community/Assisted Living | Olanzapine, quetiapine, risperidone, placebo | 36 | Mortality, stroke | National Institute of Mental Health (NIMH) |
| Shin, 2015[161] | Cohort Study | - | Haloperidol, risperidone | - | Stroke | Ministry of Food and Drug Safety |
| Sinforiani, 2011[162] | Cohort Study | Specialty Clinic | Cholinesterase inhibitor + memantine, memantine | 156 | Mortality | - |
| Sival, 2002[163] | Cross-Over RCT | Teaching Hospital | Sodium valproate, placebo | 6 | Stroke | Van Helten Foundation, Royal Netherlands |
| Sommer, 2009[164] | Parallel RCT | LTC | Oxcarbazepine, placebo | 8 | Falls | Novartis |
| Sterke, 2012[165] | Cohort Study | LTC | Antipsychotics (clozapine, olanzapine, quetiapine), antidepressants (citalopram, paroxetine, sertraline, fluvoxamine) | 43* | Falls | - |
| Street, 2000[166] | Parallel RCT | LTC | Olanzapine, placebo | 6 | Mortality, stroke | Eli Lilly |
| Streim, 2008[167] | Parallel RCT | LTC/Assisted Living | Aripiprazole, placebo | 10 | Mortality, stroke | Bristol-Myers Squibb Company and Otsuka Pharmaceutical Company, Ltd |
| Sturm, 2018[168] | Cohort Study | LTC | Quetiapine, risperidone, olanzapine, aripiprazole | >104 | Mortality, stroke, falls | Avatar Foundation, PDM Healthcare |
| Su, 2019[169] | Cohort Study | - | Mirtazapine ≥28cDDD, mirtazapine <28cDDD | - | Mortality | Chang Gung Memorial Hospital |
| Suh, 2005[170] | Cohort Study | LTC | Haloperidol+risperidone, usual care | 52 | Mortality | Janssen Korea and Eisai Korea |
| Sultana, 2014[171] | Cohort Study | - | Atypical antipsychotics, usual care | 99* | Mortality | National Institute for Health Research Mental Health Biomedical Research Centre; Dementia Unit at SLaM and (Institute of Psychiatry) King’s College London |
| Tamimi, 2012[172] | Case-Control Study | Hospital | Cholinesterase inhibitors, usual care | 261 | Fracture | PSR-SIIRI |
| Tamimi, 2018[173] | Case-Control Study | - | Cholinesterase inhibitors, usual care | NR | Fracture | CIHR |
| Tan, 2018[174] | Cohort Study | Community/Clinic/LTC | Cholinesterase inhibitors, usual care | 120* | Mortality, stroke | - |
| Tariot, 1995[175] | Quasi-Randomized Controlled Trial | LTC | Carbamazepine, placebo | 5 | Mortality | NIMH/University of Rochester |
| Tariot, 1998[176] | Parallel RCT | LTC | Carbamazepine, placebo | 6 | Falls | National Institute on Aging, Monroe Community Hospital, CIBA-Geigy Corp., Summit, N.J. |
| Tariot, 2000[177] | Parallel RCT | Home/Community | Galantamine, placebo | 22 | Mortality, falls | Janssen Research Foundation |
| Tariot, 2001a[178] | Parallel RCT | LTC | Donepezil, placebo | 24 | Mortality | Eisai, Inc.; Pfizer, Inc. |
| Tariot, 2001b[179] | Parallel RCT | LTC | Divalproex sodium, placebo | 6 | Mortality | Abbott Laboratories |
| Tariot, 2004[180] | Parallel RCT | - | Memantine, placebo | 24 | Mortality, falls | Forest Laboratories, Inc. |
| Tariot, 2005[181] | Parallel RCT | LTC | Divalproex sodium, placebo | 6 | Mortality, falls | Abbott Laboratories, National Institute on Aging |
| Tariot, 2006[182] | Parallel RCT | LTC | Quetiapine, haloperidol, placebo | 10 | Mortality, stroke, fracture, falls | AstraZeneca Pharmaceuticals LP. |
| Tariot, 2011[183] | Parallel RCT | Clinic | Valproate, placebo | 104 | Mortality, stroke, fracture, falls | Abbott Laboratories; NIA |
| Teranishi, 2013[184] | Parallel RCT | Hospital | Risperidone, fluvoxamine | 8 | Mortality, fracture, falls | - |
| Trifiro, 2007[185] | Cohort Study | Outpatient | Atypical antipsychotics, usual care | NR | Mortality | - |
| Torstensson, 2017[186] | Cohort Study | - | Risperidone, olanzapine, quetiapine, zuclopenthixol, chlorprothixen, flupentixol, levomepromazine, haloperidol, usual care | 501* | Fracture | Regional Research Foundation, Region Zealand |
| USA-63[143] | Parallel RCT | - | Risperidone, placebo | NR | Stroke | Janssen-Ortho Inc. |
| van Dyck, 2007[187] | Parallel RCT | - | Memantine, placebo | 24 | Mortality, falls | Forest Laboratories, Inc, |
| Vercelletto, 2011[188] | Parallel RCT | - | Memantine, placebo | 52 | Mortality, falls | Regional Research Grant; H. Lundbeck A/S, Copenhagen, Denmark |
| Wang, 2005[189] | Cohort Study | - | Typical antipsychotics, atypical antipsychotics | 26 | Mortality | National Institute of Mental Health; the Agency for Healthcare Research and Quality (AHRQ) |
| Watt, 2018[190] | Cohort Study | LTC | Atypical antipsychotics, trazodone | 13 | Mortality, fracture, falls | breaKThrough (knowledge translation) Program of St. Michael’s Hospital |
| Wei, 2017[191] | Cohort Study | LTC | Antidepressants, antipsychotics | 104 | Fracture, falls | Research Retirement Foundation |
| Wilcock, 2000[192] | Parallel RCT | Clinic | Galantamine, placebo | 26 | Mortality | Janssen Research Foundation |
| Wilcock, 2002[193] | Parallel RCT | Outpatient | Memantine, placebo | 28 | Mortality, stroke, falls | Merz pharmaceuticals |
| Wilcock, 2003[194] | Parallel RCT | - | Galantamine, donepezil | 52 | Mortality, falls | Janssen-Cilag UK, Janssen Pharmaceutica Products L.P., Shire Pharmaceuticals Ltd |
| Wilcock, 2008[195] | Pairwise meta-analysis | Home/community/specialty clinic | Memantine, placebo | 24-28 | Stroke | GlaxoSmithKline, Lundbeck A/S |
| Wilkinson, 2001[196] | Parallel RCT | Specialty clinic | Galantamine, placebo | 12 | Mortality | Shire Pharmaceuticals |
| Wilkinson, 2002[197] | Parallel RCT | - | Donepezil, rivastigmine | 12 | Mortality | Eisai Inc and Pfizer Inc |
| Wilkinson, 2003[198] | Parallel RCT | Clinic | Donepezil, placebo | 24 | Mortality, stroke, fracture | Eisai Inc. |
| Wilkinson, 2012[199] | Parallel RCT | Specialty Clinic | Memantine, placebo | 52 | Mortality, stroke, fracture, falls | H. Lundbeck A/S and Merz Pharmaceuticals GmbH |
| Winblad, 1999[200] | Parallel RCT | LTC | Memantine, placebo | 12 | Mortality | - |
| Winblad, 2001[201] | Parallel RCT | - | Donepezil, placebo | 52 | Mortality, fracture | Pfizer Pharmaceuticals Group, Pfizer, Inc |
| Winblad, 2006[202] | Parallel RCT | LTC | Donepezil, placebo | 26 | Mortality, fracture, falls | Pfizer |
| Winblad, 2007[203] | Parallel RCT | Specialty Clinic | Rivastigmine, placebo | 24 | Mortality | Novartis Pharma AG |
| Wu, 2015[204] | Cohort Study | - | Memantine or cholinesterase inhibitor, usual care | 238* | Mortality | National Science Council, Taiwan; Taipei Veterans General Hospital |
| Wysowski, 1996[205] | Cohort Study | - | Temazepam, triazolam | 4 | Fracture | - |
| Yin, 2015[206] | Cohort Study | Specialty Clinic | Risperidone, zolpidem, usual care | 261 | Mortality | National Natural Science Foundation of China, The Ministry of Science and Technology, Natural Science Foundation of Shanghai |
| Zhang, 2012[207] | Parallel RCT | Hospital | Galantamine, donepezil | 16 | Mortality | Xian-Janssen Pharmaceutical Ltd, Beijing, China |
| Zhong, 2007[208] | Parallel RCT | LTC | Quetiapine, placebo | 10 | Mortality, stroke, falls | AstraZeneca Pharmaceuticals. |
| Zhu, 2013[209] | Cohort Study | Clinic | Cholinesterase inhibitor, memantine, usual care | 312 | Mortality | Department of Veterans Affairs, Veterans Health Administration |

Abbreviations: long-term care (LTC), not reported (NR), randomized controlled trial (RCT)

*average length of follow-up

^companion report

# Table 2. Individual Study-Level Patient Characteristics: Randomized and Non-Randomized Studies

| **Author, Year** | **Country** | **Sample Size (overall)** | **Mean Age (years)** | **Female (%)** | **Type of Dementia** | **Mean MMSE Score** | **Mean Behavioural Score** |
| --- | --- | --- | --- | --- | --- | --- | --- |
| 5077 US-039[4] | - | 378 | 83.9 | 73.0 | Multiple | 12.8 | - |
| Aarsland, 2009[5] | Norway, Sweden, UK | 75 | 73.5 | 24.0 | Multiple | 20.0 | NPI total 13.8 |
| AD2000 Collaborative Group, 2004[6] | UK | 566 | 75.5 | 59.2 | Multiple | 19.0 | NPI-total 15 |
| Aguglia, 2004[7] | Italy | 242 | 77.2 | 66.1 | AD | 20.6 | - |
| Allain, 2000[8] | France, The Netherlands, Germany, Latvia, Portugal | 306 | 79.6 | 64.4 | - | - | - |
| Ancoli-Israel, 2005[9] | USA | 63 | 77.2 | 61.9 | AD | 19.4 | - |
| Auchus, 2007[10] | Europe, North America, Israel, Asia, Australia | 788 | 72.3 | 36.0 | VaD | 20.3 | NPI-total 11.6 |
| B351[11] | USA | 702 | 74.5 | 60.0 | AD | - | - |
| Bakchine, 2007[12] | Austria, Belgium, Denmark, Finland, France, Greece, Lithuania, the Netherlands, Poland, Spain, Sweden, UK | 470 | 73.8 | 63.2 | AD | 18.7 | - |
| Ballard, 2004[13] | UK | 100 | 83.3 | 81.0 | AD | 5.5 | NPI 14.6 |
| Ballard, 2005[14] | England | 93 | 83.8 | 79.6 | AD | 62.3 |  |
| Ballard, 2008[15] | UK | 165 | 84.8 | 76.4 | AD | 12.5 | NPI-total 16 |
| Ballard, 2008[15] | Austria, Canada, France, Germany, Italy, Korea, Russia, Spain, Switzerland, Taiwan, UK, USA | 710 | 72.8 | 37.7 | VaD | 19.2 | NPI-total 13.2 |
| Ballard, 2015[16] | UK, Norway | 199 | 83.2 | 69.3 | AD | 5.6 | NPI 15.7 |
| Banerjee, 2011[18] | UK | 326 | 79.3 | 67.8 | AD | 18.1 | NPI-total 29 |
| Barak, 2011[19] | Israel | 40 | 78.4 | 57.5 | AD | 14.5 | NPI-total 19.4 |
| Barnett, 2007[20] | USA | 14029 | 77.5 | 2.7 | Multiple | - | - |
| Baxer, 2013[21] | USA | 81 | 66.0 | 37.0 | FTD | 24.7 | NPI 21.1 |
| Black, 2003[22] | Australia, Canada, Ireland, Germany, UK, USA | 603 | 73.9 | 44.8 | VaD | 21.3 | - |
| Black, 2007[23] | United States, Canada, France, United Kingdom, Australia | 343 | 78.0 | 70.3 | AD | 7.5 | NPI 22.5 |
| Brodaty, 2003[24] | Australia, New Zealand | 345 | 82.9 | 71.8 | Multiple | 5.5 | BEHAVE-AD 18.8 |
| Brodaty, 2005[25] | United States, Australia, Canada, South Africa, New Zealand | 971 | 76.5 | 64.0 | AD | 18.0 | NPI 11.3 |
| Bronskill, 2018[26] | Canada | 10132 | 83.9 | 63.9 | Multiple | - | - |
| Bullock, 2005[27] | Australia, Canada, France, Germany, Italy, Spain, UK | 998 | 75.9 | 68.7 | LBD | 15.1 | NPI-10 14.4 |
| Burns, 1999[28] | Australia, Belgium, Canada, France, Germany, Ireland, New Zealand, South Africa, the UK | 818 | 71.7 | 57.5 | AD | 20.0 | - |
| Burns, 2009[29] | Europe | 407 | 84.0 | 81.0 | Multiple | 8.9 | - |
| Camargos, 2014[30] | Brazil | 36 | 81.0 | 66.7 | AD | 11.2 | - |
| Campbell, 2017[31] | USA | 196 | 80.2 | 74.0 | AD | - | - |
| Carlyle, 1993[32] | Canada | 40 | 79.0 | 45.0 | Multiple | - | - |
| Chan, 2001[33] | Hong Kong | 58 | 80.5 | 72.4 | Multiple | 8.0 | - |
| Chan, 2010[34] | Hong Kong | 1089 | 81.0 | 65.7 | Multiple | 12.5 | - |
| Choe, 2016[35] | Korea | 74 | 74.8 | 62.2 | AD | 16.3 | NPI-total 10.2 |
| Choi, 2011[36] | Korea | 172 |  | 79.5 | AD | - | NPI 13.7 |
| Corey-Bloom, 1998[37] | USA | 699 | 74.5 | 60.9 | AD | 19.7 | - |
| Culo, 2010[38] | USA | 103 | 82.3 | 61.9 | Multiple | 11.3 | NPI-total 36.4 |
| Cumbo, 2014[39] | Italy | 177 | 78.5 | 54.2 | AD | 16.3 | NPI 34.3 |
| Cummings, 2012[40] | North America, Europe | 567 | 75.7 | 64.7 | AD | 14.2 | NPI 13.4 |
| Cummings, 2015[41] | USA | 220 | 77.8 | 57.3 | AD | 17.3 | NPI 38.9 |
| De Deyn, 1999[42] |  | 344 | 81.3 | 56.4 | Multiple | 8.4 | BEHAVE-AD 16.5 |
| De Deyn, 2004[43] | Europe, Australia, Israel, Lebanon, South Africa | 652 | 76.6 | 75.0 | AD | - | NPI-NH 33.9 |
| De Deyn, 2005[44] | Belgium | 208 | 81.5 | 72.0 | AD | 14.2 | NPI-total 39.9 |
| de Vasconcelos Cunha, 2007[45] | Brazil | 31 | 77.6 | 74.2 | - | - | - |
| Deberdt, 2005[46] | USA | 494 | 78.3 | 65.6 | Multiple | 14.4 | NPI 42.2 |
| Devanand, 2012[47] | USA | 110 | 80.0 | 60.2 | AD | 14.2 | NPI-total 9 |
| Donepezil 319[48] | - | 974 | - | - | VaD | - | - |
| Doody, 2012[49] | - | 1467 | 73.9 | 62.8 | AD | 13.1 | - |
| Dubois, 2012[50] | Germany, Austria, Spain, Russia, UK, France, Australia, New Zealand, South Africa, Canada, Italy, Belgium, Portugal | 550 | 71.9 | 17.3 | PDD | 21.4 | NPI 13.4 |
| Dysken, 2014[51] | USA | 613 | 79.1 | 2.9 | AD | 20.8 | NPI-12 8 |
| Eady, 2018[52] | UK | 310 | 55.3 | 43.9 | - | - | - |
| Emre, 2004[53] | Austria, Belgium, Canada, France, Germany, Italy, the Netherlands, Norway, Portugal, Spain, Turkey, UK | 541 | 72.7 | 35.1 | PDD | 13.3 | NPI-10 12.9 |
| Emre, 2010[54] | Austria, France, Germany, UK, Greece, Italy, Spain, Turkey | 199 | 73.4 | 44.6 | Multiple | 20.7 | NPI 16.8 |
| Emre, 2014[55] | Europe, USA, Argentina, Canada, Australia | 583 | 72.3 | 31.7 | PDD | 20.9 | NPI-10 11.4 |
| Erkinjuntti, 2002[56] | Canada, Denmark, Finland, France, Germany, Ireland, Israel, Netherlands, Poland, UK | 592 | 75.1 | 47.3 | Multiple | 20.5 | NPI-total 11.9 |
| Farlow, 2010[57] | - | 262 | 77.2 | 57.8 | AD | 18.3 |  |
| Farlow, 2013[58] | USA | 716 | 77.0 | 64.4 | AD | 8.8 | NPI 17.1 |
| Feldman, 2001[59] | Canada, Australia, France | 291 | 73.6 | 61.0 | AD | 11.8 | NPI-total 19.4 |
| Feldman, 2007[60] | Australia, Canada, Ireland, Italy, South Africa, and UK | 678 | 71.2 | 59.0 | AD | 18.6 | - |
| Finkel, 1995[61] | USA | 35 | 85.0 | 86.0 | - | 8.8 | - |
| Finkel, 2005[62] | USA | 18987 | 79.6 | 73.2 | - | - | - |
| Fontaine, 2003[63] | USA | 39 | 83.2 | 66.7 | Multiple | 8.2 | NPI 51.7 |
| Fox, 2012[64] | UK | 153 | 84.6 | 73.8 | AD | 7.3 | NPI 36.6 |
| GAL-95-05[65] | - | 554 | - | - | AD | - | - |
| GAL-JPN-3[65] | - | 394 | - | - | AD | - | - |
| GAL-USA-16[65] | - | 139 | - | - | AD | - | - |
| Gasper, 2005[67] | USA | 10846 | 83.0 | 71.0 | - | - | - |
| Gault, 2016[68] | Russia, South Africa, UK, Ukraine, Poland, USA | 438 | 74.2 | 60.8 | AD | 18.9 | NPI-total 11.1 |
| Gerhard, 2014[69] | USA | 43183 | 79.1 | 76.2 | - | - | - |
| Gill, 2005[70] | Canada | 32710 | 82.6 | 62.8 | - | - | - |
| Gill, 2007[71] | Canada | 54518 | 82.8 | 64.9 | - | - | - |
| Gill, 2009[72] | Canada | 81302 | 80.4 | 61.5 | - | - | - |
| Gold, 2010[73] | 19 countries | 581 | - | 63.0 | AD | - | - |
| Grossberg, 2013[74] | Argentina, USA, Mexico, Chile | 677 | 76.5 | 72.0 | AD | 10.8 | NPI 16.8 |
| Hager, 2014[75] | Czech Republic, Estonia, France, Germany, Greece, Italy, Latvia, Lithuania, Romania, Russia, Slovakia, Slovenia, and Ukraine | 2051 | 73.0 | 64.8 | AD | 19.0 | - |
| Hampel, 2009[76] | Germany | 74 | 68.6 | 52.1 | AD | 23.6 | NPI-total 10.1 |
| Herrmann, 2007[77] | Canada | 14 | 85.6 | 42.9 | AD | 4.5 | NPI 33.4 |
| Herrmann, 2013[78] | Canada | 369 | 74.9 | 58.3 | AD | 11.8 | NPI-total 30 |
| Herrmann, 2016[79] | Canada | 40 | 89.3 | 20.0 | AD | 8.1 | NPI-NH 21.1 |
| HGAO[4] | - | 238 | 78.6 | 66.0 | AD | - | - |
| Homma, 1998[80] | Japan | 187 | - | - | AD | 18.7 | - |
| Homma, 2000[81] | Japan | 268 | 69.8 | 67.1 | AD | 17.2 | - |
| Homma, 2008[82] | Japan | 325 | 78.2 | 80.3 | AD | 7.8 | BEHAVE-AD 9 |
| Howard, 2007[83] | England | 272 | 84.6 | 84.6 | AD | 8.2 | NPI 23.6 |
| Howard, 2012[84] | UK | 295 | 77.1 | 65.4 | AD | 9.1 | NPI-total 22.2 |
| Hu, 2006[85] | China | 100 | 68.5 | NR | AD | 19.3 | - |
| Huybrechts, 2012[86] | USA | 45085 | - | - | - | - | - |
| Jalbert, 2010[87] | USA | 4346 | 82.5 | 73.9 | - | - | - |
| Jia, 2017[88] | China | 313 | 70.8 | 64.9 | AD | 7.3 | - |
| Johannsen, 2006[89] | Belgium, Denmark, Germany, Greece, Hungary, Iceland, The Netherlands, Poland, USA | 202 | 72.7 | 61.4 | AD | 18.6 | - |
| Kales, 2007[90] | USA | 10615 | 79.0 | 2.6 | - | - | - |
| Kales, 2012[91] | USA | 33604 | - | 2.5 | - | - | - |
| Katona, 1998[92] | Australia, Germany, Austria, France, Italy, Switzerland | 198 | 76.6 | 77.8 | - | 19.9 | - |
| Katz, 1999[93]^ | USA | 625 | 82.7 | 67.8 | Multiple | 6.6 | BEHAVE-AD 15.8 |
| Katz, 2004[94]^ | USA | 537 | 82.5 | 67.6 | Multiple | 7.0 | BEHAVE-AD 16 |
| Kennedy, 2005[95] | USA | 268 | 78.0 | 56.0 | AD | 21.5 | NPI-behaviour 0.2 |
| Kertesz, 2008[96] | Canada | 39 | 63.3 | 38.5 | FTD | 19.0 | NPI-total 20.3 |
| Kheirbek, 2019[97] | USA | 13385 | - | 1.7 | Multiple | - | - |
| Lanctot, 2002[98] | Canada | 22 | 82.0 | 45.5 | AD | 4.1 | NPI 18 |
| Langballe, 2014[99] | Norway | 26940 | 79.9 | - | - | - | - |
| Layton, 2005[100] | UK | 364 | - | 45.2 | Multiple | - | - |
| Lee, 2017[101] | South Korea | 398 | 76.0 | 65.8 | AD | 17.3 | - |
| Lin, 2016[102] | Taiwan | 10364 | - | 57.1 | Multiple | - | - |
| Liperoti, 2005[103] | USA | 4788 | - | 71.0 | Multiple | - | - |
| Liperoti, 2009[104] | USA | 9729 | 83.8 | 71.9 | Multiple | - | - |
| Litvinenko, 2008[105] | Russia | 41 | 70.6 | - | PDD | 17.8 | NPI 22.7 |
| Lopez, 1999[106] | USA | 179 | 71.3 | 55.1 | AD | - | - |
| Lopez, 2009[107] | USA | 943 | 73.1 | 67.0 | AD | 18.2 | - |
| Lopez-Pousa, 2006[108] | Spain | 404 | 78.9 | 68.2 | AD | 17.4 | - |
| Lyketsos, 2003[109] | USA | 44 | 77.5 | 68.2 | AD | 17.0 | NPI-total 35.9 |
| Maher-Edwards, 2011[110] | Austria, Bulgaria, Chile, Estonia, Germany, Russian Federation, Slovakia, and UK | 198 | 71.3 | 66.3 | AD | 19.0 | - |
| Martin, 2003[111] | USA | 730 | 81.0 | 77.0 | Multiple | - | - |
| Martinez Martinez, 2009[112] | Spain | 289 | 82.7 | 61.2 | - | - | - |
| Maust, 2015[113] | USA | 90786 | - | 2.5 | - | - | - |
| McKeith, 2000[114] | UK, Spain, Italy | 120 | 73.9 | 43.3 | LBD | 17.8 | - |
| Mintzer, 2006[115] | USA | 473 | 83.3 | 77.0 | Multiple | 13.2 | BEHAVE-AD 16.3 |
| Mintzer, 2007[116] | United States, Australia, Canada, South Africa, Argentina | 487 | 82.5 | 79.0 | AD | 12.4 | NPI-NH 41.4 |
| Mohs, 2001[117] | USA | 431 | 75.3 | 62.9 | AD | 17.1 |  |
| Mok, 2007[118] | Hong Kong | 40 | 74.9 | 60.0 | Multiple | 13.2 | NPI 12.8 |
| Moretti, 2005[119] | Italy | 356 | 76.8 | 43.9 | VaD | - | NPI 35.2 |
| Mueller, 2017[120] | UK | 2464 | 81.9 | 66.7 | AD | 19.0 | - |
| Musicco, 2011[121] | Italy | 4369 | 78.5 | 65.0 | AD | - | - |
| Nakamura, 2011[122] | Japan | 859 | 74.6 | 68.3 | AD | 16.6 | - |
| Nakamura, 2014[123] | Japan | 633 | 74.2 | 67.0 | AD | 9.9 | - |
| Nordstrom, 2013[124] | Sweden | 7073 | 79.0 | 63.4 | Multiple | 21.5 | - |
| Nyth, 1990[125] | Sweden, Norway, Finland | 98 | 77.6 | 77.5 | Multiple | - | - |
| Orgogozo, 2002[126] | France, Belgium, Switzerland | 321 | 76.4 | 47.2 | VaD | 16.9 | - |
| Pakdaman, 2015[127] | Iran | 264 | 72.3 | 56.8 | AD | 17.6 | - |
| Paleacu, 2008[128] | Israel | 40 | 82.2 | 65.0 | AD | 14.6 | - |
| Peskind, 2006[129] | USA | 403 | 77.5 | 58.8 | AD | 17.3 | NPI 6.3 |
| Peters, 2015[130] | Germany | 226 | 72.4 | 63.7 | AD | 22.2 | NPI 6.8 |
| Piersanti, 2014[131] | Italy | 696 | 81.1 | 66.2 | Multiple | - | - |
| Porsteinsson, 2001[132] | USA | 56 | 85.0 | 69.6 | Multiple | 6.9 | BPRS 55.2 |
| Porsteinsson, 2008[133] | USA | 433 | 75.4 | 52.2 | AD | 16.8 | NPI 12 |
| Porsteinsson, 2014[134] | USA, Canada | 186 | 78.0 | 46.1 | AD | 15.7 | NPI 37.3 |
| Profenno, 2005[135] | USA | 20 | 77.6 | 55.0 | AD | 16.1 | NPI-total 4 |
| Rafaniello, 2014[136] | Italy | 1618 | 80.3 | 67.8 | Multiple | - | - |
| Rainer, 2007[137] | Austria | 72 | 77.8 | 58.5 | Multiple | 18.3 | NPI 25.6 |
| Raivio, 2007[138] | Finland | 254 | 86.0 | 85.0 | - | 9.9 | - |
| Rappaport, 2009[139] | USA | 129 | 79.9 | 64.3 | Multiple | 9.5 | - |
| Raskind, 2000[140] | USA | 636 | 75.4 | 61.9 | AD | 19.3 | - |
| Raskind, 2000; Wilcock, 2000[48] | Europe, Canada, USA | 1289 | 75.4 | 61.9 | AD | 19.3 | - |
| Reisberg, 2003[141] | USA | 252 | 76.1 | 67.5 | AD | 7.9 | NPI-12 20.5 |
| RIS-BEL-14[142, 143] | - | 39 | 78.0 | - | AD | - | - |
| RIS-INT-83[142, 144] | - | 18 | 86.0 | - | AD | - | - |
| Rochon, 2008[145] | Canada | 41241 | 83.3 | 66.1 | - | - | - |
| Rockwood, 2001[146] | USA, Canada, UK, South Africa, Australia, New Zealand | 386 | 75.0 | 55.7 | AD | 19.7 | NPI-total 9.3 |
| Rogers, 1998a[147] | USA | 468 | 73.7 | 63.7 | AD | 19.5 | - |
| Rogers, 1998b[148] | USA | 473 | 73.4 | 61.9 | AD | 19.0 | - |
| Roman, 2010[149] | USA | 974 | 73.0 | 41.1 | VaD | 23.5 | - |
| Rosenberg, 2010[150] | USA | 131 | 77.3 | 54.2 | AD | 20.0 | - |
| Rosenberg, 2013[151] | USA, Canada | 60 | 76.0 | 62.0 | AD | 20.0 | NPI-total 16 |
| Rosler, 1999[152] | Austria, France, Germany, Switzerland, North America | 725 | 72.0 | 59.0 | AD | 19.9 | - |
| Rossom, 2010[153] | USA | 90635 | 78.0 | 2.8 | Multiple | - | - |
| Ruths, 2008[154] | Norway | 55 | 84.1 | 78.2 | - | - | NPI 8.2 |
| Sahlberg, 2015[155] | Denmark | 9953 | 82.0 | 61.0 | - | - | - |
| Santos-Garcia, 2010[156] | Spain | 133 | 81.9 | 63.2 | - | - | NPI-total 50.2 |
| Saxton, 2012[157] | South Africa, New Zealand, Australia | 265 | 74.9 | 58.3 | AD | 15.8 | - |
| Scarpini, 2011[158] | Italy | 139 | 74.5 | 59.7 | AD | 21.6 | - |
| Schneeweiss, 2007[159] | USA | 4334 | 80.2 | 63.3 | - | - | - |
| Schneider, 2006[160] | USA | 421 | 77.9 | 56.0 | AD | 15.0 | NPI-total 36.9 |
| Shin, 2015[161] | Korea | 16039 | 75.0 | 58.7 | - | - | - |
| Sinforiani, 2011[162] | Italy | 201 | 78.6 | 60.2 | AD | 13.4 | NPI-total 10.3 |
| Sival, 2002[163] | Netherlands | 43 | 80.4 | 59.5 | Multiple | 11.4 | - |
| Sommer, 2009[164] | Norway | 103 | 84.0 | 70.9 | Multiple | 5.8 | NPI 45.5 |
| Sterke, 2012[165] | Netherlands | 248 | 82.0 | 59.7 | - | - | - |
| Street, 2000[166] | USA | 206 | 74.8 | 54.7 | AD | 13.0 | - |
| Streim, 2008[167] | USA | 256 | 83.0 | 59.4 | AD | 13.6 | NPI-NH 37.8 |
| Sturm, 2018[168] | USA | 88 | 78^ | 55 | Multiple | 14.4 | - |
| Su, 2019[169] | Taiwan | 25890 | - | 54.9 | - | - | - |
| Suh, 2005[170] | Korea | 273 | 79.5 | 82.8 | Multiple | 10.6 | BEHAVE-AD 15.8 |
| Sultana, 2014[171] | UK | 1531 | 81.0 | 57.7 | VaD | - | - |
| Tamimi, 2012[172] | Spain | 2258 | 82.5 | 71.5 | AD | 15.4 | - |
| Tamimi, 2018[173] | UK | 5950 | - | 76.4 | Multiple | - | - |
| Tan, 2018[174] | Sweden | 23144 | 79.7 | 59.4 | Multiple | 20.4 | - |
| Tariot, 1995[175] | USA | 25 | 84.5 | 76.0 | Multiple | - | - |
| Tariot, 1998[176] | USA | 51 | 86.0 | 80.0 | Multiple | 6.0 | BPRS 54.2 |
| Tariot, 2000[177] | USA | 978 | 76.9 | 63.9 | AD | 17.8 | NPI-total 11.9 |
| Tariot, 2001a[178] | USA | 208 | 85.7 | 82.5 | Multiple | 14.4 | NPI-total 20.7 |
| Tariot, 2001b[179] | USA | 173 | 83.3 | 64.5 | Multiple | 7.4 | BPRS 42.6 |
| Tariot, 2004[180] | USA | 404 | 75.5 | 65.3 | AD | 10.0 | NPI 13.4 |
| Tariot, 2005[181] | USA | 153 | 84.0 | 68.6 | AD | 10.7 | BPRS 34.2 |
| Tariot, 2006[182] | USA | 284 | 83.2 | 73.0 | Multiple | 12.8 | NPI-NH2 9.9 |
| Tariot, 2011[183] | USA | 313 | 75.8 | 58.8 | AD | 16.9 | NPI 2.9 |
| Teranishi, 2013[184] | Japan | 82 | 82.5 | 67.1 | Multiple | 4.7 | NPI-NH 24 |
| Trifiro, 2007[185] | Netherlands | 4421 | 84.6 | 72.4 | Multiple | - | - |
| Torstensson, 2017[186] | Denmark | 33682 | 71.1 | 54.6 | - | - | - |
| USA-63[143] | - | 625 | - | - | - | - | - |
| van Dyck, 2007[187] | USA | 350 | 78.2 | 71.4 | AD | 10.1 | NPI 18.9 |
| Vercelletto, 2011[188] | France | 52 | 65.6 | 36.7 | FTD | 24.8 | NPI-total 29.5 |
| Wang, 2005[189] | USA | 10948 | 83.4 | 81.1 | - | - | - |
| Watt, 2018[190] | Canada | 9463 | 85.3 | 68.7 | Multiple | - | - |
| Wei, 2017[191] | USA | 6644 | 82.9 | 73.0 | AD | - | - |
| Wilcock, 2000[192] | Canada, European Union | 653 | 72.2 | 62.6 | AD | 19.3 | - |
| Wilcock, 2002[193] | UK | 579 | 77.4 | 48.7 | VaD | 17.6 | - |
| Wilcock, 2003[194] | UK | 188 | 73.5 | 62.1 | AD | 15.0 | - |
| Wilcock, 2008[195] | USA | 604 | 76.8 | 67.5 | AD | 9.4 | NPI total 23.6 |
| Wilkinson, 2001[196] | UK | 285 | 73.7 | 57.7 | AD | 18.7 | - |
| Wilkinson, 2002[197] | UK, South Africa, Switzerland | 112 | 74.4 | 58.6 | AD | 21.1 | - |
| Wilkinson, 2003[198] | USA, Canada, Europe, Australia | 616 | 75.0 | 40.1 | VaD | 21.5 | - |
| Wilkinson, 2012[199] | France, Germany, Switzerland, and United Kingdom | 278 | 74.1 | 57.0 | AD | 16.9 | NPI 13 |
| Winblad, 1999[200] | Latvia | 167 | 71.6 | 57.8 | Multiple | 6.3 | - |
| Winblad, 2001[201] | Denmark, Finland, Norway, Sweden, Netherlands | 286 | 72.5 | 64.3 | AD | 19.3 | NPI 12.4 |
| Winblad, 2006[202] | Sweden | 249 | 84.9 | 76.6 | AD | 6.1 | NPI 19.3 |
| Winblad, 2007[203] | Europe, USA | 1195 | 73.6 | 66.5 | AD | 16.5 | NPI-12 14.8 |
| Wu, 2015[204] | Taiwan | 12193 | 80.4 | - | - | - | - |
| Wysowski, 1996[205] | USA | - | - | 75.0 | AD | - | - |
| Yin, 2015[206] | China | 156 | 76.7 | 62.4 | AD | 17.3 | NPI-total 4.8 |
| Zhang, 2012[207] | China | 233 | 73.6 | 52.8 | AD | 18.4 | - |
| Zhong, 2007[208] | USA | 333 | 83.2 | 74.2 | Multiple | 5.3 | NPI-NH 36.5 |
| Zhu, 2013[209] | USA | 201 | 76.3 | 61.2 | AD | 21.9 | - |

^median

Abbreviations: Mini-Mental State Examination (MMSE), United Kingdom (UK), United States of America (USA), Alzheimer’s disease (AD), vascular dementia (VaD), frontotemporal dementia (FTD), Parkinson disease dementia (PDD), Neuropsychiatric Inventory (NPI), Neuropsychiatric Inventory – Nursing Home (NPI-NH), Brief Psychiatric Rating Scale (BPRS), Behavioral Pathology in Alzheimer’s Disease Rating Scale (BEHAVE-AD).

# Table 3. Arm-Level Data for RCTs Included in Pairwise and Network Meta-Analyses

| **Author, Year** | **Fracture (#events/#participants)** | **Mortality (#events/#participants)** | **Cerebrovascular Event (#events/#participants)** | **Falls (#events/#participants)** |
| --- | --- | --- | --- | --- |
|  |  |  |  |  |
| 5077 US-039[4] | - | Quetiapine (4/124), placebo (4/125) | - | - |
| Aarsland, 2009[5] | - | - | Memantine (1/35), placebo (1/40) | - |
| AD2000 Collaborative Group, 2004[6] | - | Donepezil (63/282), placebo (50/283) | - | - |
| Allain, 2000[8] | - | Haloperidol (2/101), placebo (1/103) | - | - |
| Auchus, 2007[10, 48] | - | Galantamine (5/397), placebo (11/390) | Galantamine (10/397), placebo (14/390) | Galantamine (29/397), placebo (36/390) |
| B351[11] | Rivastigmine (7/522), placebo (1/172) | - | - | - |
| Bakchine, 2007[12, 48] | - | Memantine (5/318), placebo (2/152) | Memantine (2/318), placebo (0/152) | Memantine (1/318), placebo (0/152) |
| Ballard, 2004[13] | - | Antipsychotics (3/54), placebo (3/46) | - | - |
| Ballard, 2005[14] | - | Quetiapine (2/31), rivastigmine (2/31), placebo (0/31) | - | - |
| Ballard, 2008[15] | - | Rivastigmine (8/365), placebo (4/345) | Rivastigmine (20/365), placebo (15/345) | Rivastigmine (24/365), placebo (17/345) |
| Ballard, 2008[210] | - | Antipsychotics (7/83), placebo (9/82) | - | - |
| Ballard, 2015[16] | - | Memantine (9/100), antipsychotics (4/99) | Memantine (0/100), antipsychotics (3/99) | - |
| Ballard, 2018[17] | Pimavanserin (1/90), placebo (0/91) | Pimavanserin (4/90), placebo (4/91) | Pimavanserin (1/90), placebo (0/91) | Pimavanserin (21/90), placebo (21/91) |
| Banerjee, 2011[18] | - | Sertraline+mirtazapine (10/215), placebo (5/111) | - | - |
| Barak, 2011[19] | - | - | - | Risperidone (2/20), escitalopram (0/20) |
| Baxer, 2013[21] | - | - | - | Memantine (5/39), placebo (2/42) |
| Black, 2003[22, 211] | - | Donepezil (8/404), placebo (7/199) | Donepezil (20/404), placebo (6/199) | - |
| Black, 2007[23] | - | Donepezil (2/176), placebo (8/167) | - | - |
| Brodaty, 2003[24] | - | Risperidone (6/167), placebo (5/170) | Risperidone (15/167), placebo (3/170) | Risperidone (42/167), placebo (46/170) |
| Brodaty, 2005[25, 48] | - | Galantamine (6/645), placebo (2/320) | - | Galantamine (40/645), placebo (19/320) |
| Burns, 1999[28] | - | Donepezil (3/544), placebo (2/274) | - | - |
| Burns, 2009[29, 48] | - | Galantamine (9/207), placebo (22/200) | - | Galantamine (24/207), placebo (22/200) |
| Camargos, 2014[30] | Trazodone (0/19), placebo (1/17) | - | - | - |
| Choe, 2016[35] | - | Escitalopram (1/37), placebo (0/37) | Escitalopram (0/37), placebo (1/37) | Escitalopram (0/37), placebo (1/37) |
| Choi, 2011[36] | Memantine + rivastigmine patch (2/88), rivastigmine patch (0/84) | - | - | - |
| Corey-Bloom, 1998[11, 37] | Rivastigmine (7/464), placebo (3/235) | Rivastigmine (1/464), placebo (0/235) | - | - |
| Cumbo, 2014[39] | - | Memantine (6/48), donepezil + galantamine + rivastigmine (15/129) | - | - |
| Cummings, 2015[41] | - | Dextromethorphan-quinidine (0/93), placebo (0/127)* | Dextromethorphan-quinidine (1/93), placebo (1/27) | Dextromethorphan-quinidine (13/93), placebo (5/127) |
| De Deyn, 1999[42, 142, 143] | - | Risperidone (1/115), placebo (5/114) | Risperidone (9/115), placebo (2/114) | - |
| De Deyn, 2004[43, 212] | - | Olanzapine (15/520), placebo (2/129) | Olanzapine (4/520), placebo (0/129) | - |
| De Deyn, 2005[44]^ | Aripiprazole (2/106), placebo (0/102) | Aripiprazole (4/106), placebo (0/102) | Aripiprazole (1/106), placebo (1/102) | Aripiprazole (1/106), placebo (1/102) |
| de Vasconcelos Cunha, 2007[45] | - | - | - | Venlafaxine (1/14), placebo (0/17) |
| Deberdt, 2005[4, 46] | - | Risperidone+olanzapine (10/400), placebo (1/94) | Risperidone+olanzapine (9/400), placebo (0/94) | Risperidone+olanzapine (41/400), placebo (6/94) |
| Devanand, 2012[47] | Risperidone (0/70), placebo (0/40)* | Risperidone (2/70), placebo (1/40) | - | Risperidone (2/70), placebo (1/40) |
| Donepezil 319[48] | Donepezil (5/648), placebo (2/326) | - | - | - |
| Doody, 2012[49] | - | Donepezil + memantine (7/520), donepezil (8/914) | - | Donepezil + memantine (4/520), donepezil (4/914) |
| Dubois, 2012[50] | Donepezil (3/377), placebo (0/173) | Donepezil (5/377), placebo (5/173) | - | Donepezil (22/377), placebo (9/173) |
| Dysken, 2014[51]^ | Memantine (1/155), placebo (0/152) | Memantine (39/155), placebo (31/152) | Memantine (1/155), placebo (4/152) | Memantine (28/155), placebo (31/152) |
| Emre, 2004[48, 53] | - | Rivastigmine (4/362), placebo (7/179) | - | Rivastigmine (21/362), placebo (11/179) |
| Emre, 2010[54] | - | Memantine (3/98), placebo (3/101) | Memantine (3/98), placebo (0/101) | Memantine (8/98), placebo (8/101) |
| Erkinjuntti, 2002[56] | - | Galantamine (9/396), placebo (7/196) | Galantamine (8/396), placebo (6/196) |  |
| Farlow, 2010[57] | Rivastigmine + memantine (1/135), rivastigmine (0/126) | Rivastigmine + memantine (1/135), rivastigmine (0/126) | Rivastigmine + memantine (1/135), rivastigmine (0/126) | Rivastigmine + memantine (6/135), rivastigmine (7/126) |
| Feldman, 2001[59] | - | Donepezil (1/144), placebo (0/146) | - | - |
| Feldman, 2007[11, 60] | Rivastigmine (1/228), placebo (2/222) | Rivastigmine (0/456), placebo (0/222)* | - | - |
| Finkel, 1995[61] | - | Thiothixene (0/17), placebo (4/18) | - | - |
| Fox, 2012[64] | - | Memantine (5/74), placebo (7/79) | - | - |
| GAL-95-05[66] | - | Galantamine (2/275), placebo (2/279) | - | - |
| GAL-JPN-3[65] | - | Galantamine (2/256), placebo (2/138) | - | - |
| GAL-USA-16[65] | - | Galantamine (1/70), placebo (0/69) | - | - |
| Gault, 2016[68] | - | Donepezil (2/76), placebo (0/104) | - | Donepezil (5/76), placebo (4/104) |
| Gold, 2010[73] | Donepezil (1/84), placebo (3/166) | Donepezil (0/84), placebo (1/166) | Donepezil (1/84), placebo (1/166) |  |
| Grossberg, 2013[74]^ | Memantine (0/341), placebo (3/335) | Memantine (4/341), placebo (5/335) | Memantine (2/341), placebo (0/335) | Memantine (19/341), placebo (26/335) |
| Hager, 2014[75]^ | Galantamine (2/1024), placebo (3/1021) | Galantamine (33/1024), placebo (56/1021) | Galantamine (4/1024), placebo (2/1021) | Galantamine (2/1024), placebo (3/1021) |
| Herrmann, 2013[78]^ | Memantine (1/182), placebo (0/187) | Memantine (1/182), placebo (1/187) | Memantine (1/182), placebo (1/187) | Memantine (20/182), placebo (8/187) |
| Herrmann, 2016[79] | - | Galantamine (0/21), placebo (1/19) | - | Galantamine (3/21), placebo (3/19) |
| HGAO[4, 212] | - | Olanzapine (3/120), placebo (2/118) | Olanzapine (0/118), placebo (1/118) | - |
| Homma, 1998[80] | - | Donepezil (1/128), placebo (0/59) | - | - |
| Homma, 2000[81] | Donepezil (1/136), placebo (3/131) | - | - | - |
| Homma, 2008[82] | - | Donepezil (4/197), placebo (1/105) | - | Donepezil (13/197), placebo (6/105) |
| Howard, 2007[48, 213] | Donepezil (2/128), placebo (0/131) | Donepezil (3/128), placebo (4/131) | Donepezil (1/128), placebo (0/131) | Donepezil (2/128), placebo (2/131) |
| Howard, 2012[84] | - | Donepezil + memantine (7/73), memantine (10/76), donepezil (13/73), placebo (10/73) | Donepezil + memantine (3/73), memantine (1/76), donepezil (5/73), placebo (3/73) | Donepezil + memantine (3/73), memantine (8/76), donepezil (9/73), placebo (12/73) |
| Hu, 2006[85] | - | Memantine (0/50), donepezil (1/50) | - | - |
| Jia, 2017[88] | - | Donepezil (1/157), placebo (3/156) | Donepezil (3/157), placebo (3/156) | - |
| Johannsen, 2006[89] | - | Donepezil (1/99), placebo (1/103) | - | - |
| Katz, 1999[93] | - | Risperidone (25/462), placebo (5/163) | - | Risperidone (84/462), placebo (33/163) |
| Katz, 2004[94] | Risperidone (4/398), placebo (4/139) | - | - | - |
| Kennedy, 2005[95] | - | Olanzapine (1/178), placebo (1/90) | Olanzapine (3/178), placebo (1/90) | - |
| Kertesz, 2008[96] | - | Rivastigmine (0/18), placebo (1/18) | - | - |
| Lyketsos, 2003[109] | - | Sertraline (0/24), placebo (1/20) | - | Sertraline (1/24), placebo (2/20) |
| Maher-Edwards, 2011[110] | - | Donepezil (2/67), placebo (0/62) | - | - |
| McKeith, 2000[114] | - | Rivastigmine (0/59), placebo (2/61) | - | - |
| Mintzer, 2006[115] | - | Risperidone (9/235), placebo (6/238) | Risperidone (4/235), placebo (1/238) | Risperidone (26/235), placebo (30/238) |
| Mintzer, 2007[116] | - | Aripiprazole (15/366), placebo (3/121) | Aripiprazole (7/366), placebo (0/121) | - |
| Mohs, 2001[117] | - | Donepezil (3/214), placebo (4/217) | - | - |
| Mok, 2007[118] | - | Rivastigmine (0/20), placebo (1/20) | Rivastigmine (1/20), placebo (4/20) | - |
| Nakamura, 2011[122]^ | Rivastigmine patch (3/571), placebo (0/288) | Rivastigmine patch (1/571), placebo (1/288) | Rivastigmine patch (4/571), placebo (1/288) | Rivastigmine patch (0/571), placebo (0/288)* |
| Nakamura, 2014[123] | - | - | - | Memantine (31/321), placebo (33/319) |
| Nyth, 1990[125] | - | Citalopram (1/44), placebo (0/45) | - | - |
| Paleacu, 2008[128] | - | - | - | Quetiapine (2/20), placebo (0/20) |
| Peskind, 2006[48, 129] | - | Memantine (1/201), placebo (1/202) | - | Memantine (15/201), placebo (15/202) |
| Peters, 2015[130] | - | - | - | Cholinesterase inhibitors (18/112), placebo (14/114) |
| Porsteinsson, 2001[132] | - | - | Divalproex sodium (1/28), placebo (0/28) | Divalproex sodium (7/28), placebo (5/28) |
| Porsteinsson, 2008[48, 133] | - | Cholinesterase inhibitors + memantine (3/217), cholinesterase inhibitor (2/216) | - | Cholinesterase inhibitors + memantine (22/217), cholinesterase inhibitor (15/216) |
| Profenno, 2005[135] | - | - | - | Divalproex (2/16), placebo (0/4) |
| Rappaport, 2009[139] | Aripiprazole (1/103), placebo (0/26) | Aripiprazole (1/103), placebo (0/26) | Aripiprazole (1/103), placebo (0/26) | Aripiprazole (1/103), placebo (1/26) |
| Raskind, 2000[140] | - | Galantamine (2/423), placebo (1/213) | - | - |
| Raskind, 2000; Wilcock, 2000[48] | - | - | - | Galantamine (46/861), placebo (28/428) |
| Reisberg, 2003[141, 214] | - | Memantine (2/126), placebo (5/126) | - | Memantine (9/126), placebo (9/126) |
| RIS-BEL-14[142, 143] | - | Risperidone (1/20), placebo (0/19) | Risperidone (0/20), placebo (0/19)* | - |
| RIS-INT-83[142, 144] | - | Risperidone (0/10), placebo (1/8) | Risperidone (0/10), placebo (0/8)* | - |
| Rockwood, 2001[146] | - | Galantamine (0/261), placebo (2/125) | - | - |
| Rogers, 1998[147] | - | Donepezil (0/315), placebo (1/153) | Donepezil (1/315), placebo (0/153) | - |
| Rogers, 1998[148] | Donepezil (4/311), placebo (0/162) | Donepezil (1/311), placebo (1/162) | Donepezil (1/311), placebo (1/162) | - |
| Roman, 2010[149] | - | Donepezil (11/648), placebo (0/326) | - | - |
| Rosenberg, 2010[150] | - | Sertraline (0/67), placebo (1/64) | - | Sertraline (16/67), placebo (11/64) |
| Rosler, 1999[11, 152] | Rivastigmine (5/486), placebo (3/239) | Rivastigmine (1/486), placebo (0/239) | - | - |
| Ruths, 2008[154] |  | Antipsychotics (1/28), placebo (3/28) |  |  |
| Saxton, 2012[157]^ | Memantine (0/136), placebo (1/129) | Memantine (0/136), placebo (1/129) | Memantine (0/136), placebo (1/129) | Memantine (4/136), placebo (4/129) |
| Scarpini, 2011[158] | - | Galantamine (5/76), placebo (2/63) | - | - |
| Schneider, 2006[160] | - | Olanzapine+quetiapine+ risperidone (5/279), placebo (3/142) | Olanzapine+quetiapine+ risperidone (4/279), placebo (1/142) |  |
| Sommer, 2009[164] | - | - | - | Oxcarbazepine (10/52), placebo (8/51) |
| Street, 2000[4, 166, 212] | - | Olanzapine (6/159), placebo (0/47) | Olanzapine (1/159), placebo (0/47) | - |
| Streim, 2008[167] | - | Aripiprazole (3/131), placebo (3/125) | Aripiprazole (0/131), placebo (1/125) | - |
| Tariot, 1998[176] | - | - | - | Carbamazepine (12/27), placebo (11/24) |
| Tariot, 2000[48, 177] | - | Galantamine (7/692), placebo (4/286) | - | Galantamine (26/692), placebo (14/286) |
| Tariot, 2001a[178] | - | Donepezil (3/103), placebo (7/105) | - | - |
| Tariot, 2001b[179] | - | Divalproex sodium (5/87), placebo (1/85) | - | - |
| Tariot, 2004[180, 215] | - | Memantine (1/203), placebo (2/201) | - | Memantine (15/203), placebo (14/201) |
| Tariot, 2005[181] | - | Divalproex sodium (1/75), placebo (0/78) | - | Divalproex sodium (16/75), placebo (13/78) |
| Tariot, 2006[182] | Quetiapine+haloperidol (8/185), placebo (7/99) | Quetiapine+ haloperidol (9/185), placebo (4/99) | Quetiapine+haloperidol (3/185), placebo (3/99) | Quetiapine+haloperidol (53/185), placebo (28/99) |
| Tariot, 2011[183]^ | Valproate (0/153), placebo (2/160) | Valproate (9/153), placebo (12/160) | Valproate (0/153), placebo (1/160) | Valproate (60/153), placebo (51/160) |
| Teranishi, 2013[184] | Risperidone (0/27), fluvoxamine (0/28)* | Risperidone (2/27), fluvoxamine (0/28) | - | Risperidone (1/27), fluvoxamine (1/28) |
| USA-63[143] | - | - | Risperidone (5/462), placebo (2/163) | - |
| van Dyck, 2007[48, 187] | - | Memantine (5/178), placebo (3/172) | - | Memantine (10/178), placebo (17/172) |
| Vercelletto, 2011[188] | - | Memantine (2/23), placebo (0/26) | - | Memantine (0/23), placebo (2/26) |
| Wilcock, 2000[65, 192] | - | Galantamine (2/438), placebo (2/215) | - | - |
| Wilcock, 2002[193, 214] | - | Memantine (9/295), placebo (9/284) | Memantine (14/295), placebo (17/284) | Memantine (18/295), placebo (21/284) |
| Wilcock, 2008[195] | - | - | Memantine (1/310), placebo (4/294) | - |
| Wilkinson, 2001[65, 196] | - | Galantamine (1/198), placebo (0/87) | - | - |
| Wilkinson, 2003[198, 211] | Donepezil (6/423), placebo (2/193) | Donepezil (5/423), placebo (1/193) | Donepezil (15/423), placebo (11/193) | - |
| Wilkinson, 2012[199]^ | Memantine (0/133), placebo (1/144) | Memantine (3/133), placebo (1/144) | Memantine (1/133), placebo (0/144) | Memantine (8/133), placebo (9/144) |
| Winblad, 1999[200] | - | Memantine (4/82), placebo (4/84) | - | - |
| Winblad, 2001[201] | Donepezil (8/142), placebo (5/144) | Donepezil (4/142), placebo (3/144) | - | - |
| Winblad, 2006[48, 202] | Donepezil (7/128), placebo (4/120) | Donepezil (18/128), placebo (19/120) | - | Donepezil (17/128), placebo (15/120) |
| Winblad, 2007[216] | - | Rivastigmine (12/893), placebo (4/302) | - | - |
| Zhong, 2007[208] | - | Quetiapine (15/241), placebo (3/92) | Quetiapine (2/241), placebo (1/92) | Quetiapine (63/241), placebo (24/92) |

*zero events in all treatment arms - excluded from pairwise and network meta-analyses; ^data found at clinicaltrials.gov

# Table 4a. Assessment of Transitivity: RCTs Reporting Fractures

| **Treatment Comparison** | **No. of Studies (No. of Patients)** | **Age (years)** | **Sex** | **Study Setting** | **Type of Fracture** | **Study Duration (weeks)** | **Type of Dementia** | **Severity of Dementia** | **RoB: Missing Data** | **RoB: Randomization Procedure** |
| --- | --- | --- | --- | --- | --- | --- | --- | --- | --- | --- |
| Anticonvulsant vs. Placebo [183] | 1 (313) | 70-79.9 | ≥50% | Clinic | Hip | >30 | AD | Moderate | High | Low |
| Antidepressant vs. Placebo [30] | 1 (36) | ≥80 | ≥50% | Clinic | Arm | <13 | AD | Mild/  moderate/  severe | High | Low |
| Antipsychotics vs. Placebo [17, 44, 94, 139, 182] | 5 (1339) | ≥80 | ≥50% | LTC | Hip | <13 | Multiple | Mild/  moderate/  severe | High | Unclear |
| Cholinesterase Inhibitor vs. Placebo [11, 37, 48, 50, 60, 73, 75, 82, 122, 148, 152, 198, 201, 202, 213] | 15 (9395) | 70-79.9 | ≥50% | Home/  community/  LTC/assisted living | Hip | >30 | AD | Mild/  moderate | High | Low |
| Memantine vs. Placebo [51, 74, 78, 157, 199] | 5 (1894) | 70-79.9 | ≥50% | Clinic | Hip | >30 | AD | Moderate/  severe | High | Low |
| Memantine + Cholinesterase Inhibitor vs. Cholinesterase Inhibitor [36, 57] | 2 (433) | 70-79.9 | ≥50% | Not stated | Hip/  femur | 13-30 | AD | Mild/  moderate | High | Unclear |

Abbreviations: long-term care (LTC), Alzheimer’s disease (AD), number (No.), risk of bias (RoB)

# Table 4b. Assessment of Transitivity: RCTs Reporting Mortality

| **Treatment Comparison** | **No. of Studies (No. of Patients)** | **Age (years)** | **Sex** | **Study Setting** | **Study Duration (weeks)** | **Type of Dementia** | **Severity of Dementia** | **RoB: Missing Data** | **RoB: Randomization Procedure** |
| --- | --- | --- | --- | --- | --- | --- | --- | --- | --- |
| Anticonvulsants vs. Placebo [179, 181, 183] | 3 (638) | ≥80 | ≥50% | LTC | >30 | AD/multiple | Mild/moderate/  severe | High | Low |
| Antidepressants vs. Placebo [18, 35, 109, 150, 217] | 5 (664) | 70-79.9 | ≥50% | Community  /clinic/assisted living/LTC | 13-30 | AD | Mild/moderate | High | Low |
| Antidepressants vs. Antipsychotics [184] | 1 (55) | ≥80 | ≥50% | Hospital | <13 | AD/VaD/LBD | Moderate/severe | Low | Low |
| Antipsychotics vs. Memantine [16] | 1 (199) | ≥80 | ≥50% | LTC | 13-30 | AD | Moderate/severe | High | Low |
| Antipsychotics vs. Cholinesterase Inhibitors [14] | 1 (62) | ≥80 | ≥50% | Care facility | 13-30 | AD | Mild/moderate/  severe | High | Low |
| Antipsychotics vs. Placebo [4, 8, 13, 14, 17, 24, 42-44, 46, 47, 61, 93, 95, 115, 116, 139, 142, 154, 160, 166, 167, 182, 208, 210] | 27 (6856) | ≥80 | ≥50% | LTC | 13-30 | AD | Mild/moderate/  severe | High | Unclear |
| Cholinesterase Inhibitors vs. Memantine [39, 84, 85] | 3 (426) | 70-79.9 | ≥50% | Clinic | >30 | AD | Mild/moderate | High | Low |
| Cholinesterase Inhibitors vs. Placebo [6, 10, 14, 15, 22, 23, 25, 28, 29, 37, 50, 53, 56, 59, 65, 66, 68, 73, 75, 79, 80, 82-84, 88, 89, 96, 110, 114, 117, 118, 122, 140, 146-149, 152, 158, 177, 178, 192, 196, 198, 201-203] | 48 (22828) | 70-79.9 | ≥50% | Clinic | 13-30 | AD | Mild/moderate | High | Low |
| Cholinesterase Inhibitors + Memantine vs. Placebo [84] | 1 (146) | 70-79.9 | ≥50% | Clinic | >30 | AD | Moderate/severe | High | Low |
| Cholinesterase Inhibitors + Memantine vs. Cholinesterase Inhibitor [49, 57, 84, 133] | 4 (2274) | 70-79.9 | ≥50% | Clinic/  community | 13-30 | AD | Mild/moderate/  severe | High | Low |
| Cholinesterase Inhibitors + Memantine vs. Memantine [84] | 1 (149) | 70-79.9 | ≥50% | Clinic | >30 | AD | Moderate/severe | High | Low |
| Memantine vs. Placebo [12, 51, 54, 64, 74, 78, 84, 129, 141, 157, 180, 187, 188, 193, 199, 200] | 16 (5068) | 70-79.9 | ≥50% | Clinic/  community | >30 | AD | Moderate/severe | High | Low |

Abbreviations: long-term care (LTC), number (No.), risk of bias (RoB), Alzheimer’s disease (AD), vascular dementia (VaD), Lewy body dementia (LBD)

# Table 4c. Assessment of Transitivity: RCTs Reporting Stroke

| **Treatment Comparison** | **No. of Studies (No. of Patients)** | **Age (years)** | **Sex** | **Study Setting** | **Type of Stroke** | **Study Duration (weeks)** | **Type of Dementia** | **Severity of Dementia** | **RoB: Missing Data** | **RoB: Randomization Procedure** |
| --- | --- | --- | --- | --- | --- | --- | --- | --- | --- | --- |
| Anticonvulsant vs. Placebo [132, 183] | 2 (369) | 70-79.9 | ≥50% | Clinic/  LTC | Not specified | >30 | AD | Mild/  Moderate/  severe | High | Unclear |
| Antidepressant vs. Placebo [35] | 1 (74) | 70-79.9 | ≥50% | Specialty clinic | Hemorrhagic | >30 | AD | Mild/ moderate | High | Low |
| Antipsychotics vs. Placebo [17, 24, 43, 44, 46, 95, 115, 116, 139, 143, 160, 166, 167, 182, 208, 212] | 17 (5816) | ≥80 | ≥50% | LTC/  assisted living | Not specified | <13 | AD | Mild/  moderate | High | Unclear |
| Cholinesterase Inhibitor vs. Memantine [84] | 1 (149) | 70-79.9 | ≥50% | Clinic | Not specified | >30 | AD | Moderate/  severe | High | Low |
| Cholinesterase Inhibitor vs. Placebo [10, 22, 56, 73, 75, 83, 84, 88, 118, 122, 147, 148, 198, 210] | 14 (8161) | 70-79.9 | ≥50% | Home/  community/  clinic | Not specified | >30 | AD | Mild/  moderate | High | Low |
| Cholinesterase Inhibitor + Memantine vs. Cholinesterase Inhibitor [57, 84] | 2 (407) | 70-79.9 | ≥50% | Clinic | Not specified | 13-30 | AD | Mild/  moderate/  severe | High | Unclear |
| Cholinesterase Inhibitor + Memantine vs. Memantine [84] | 1 (149) | 70-79.9 | ≥50% | Clinic | Not specified | >30 | AD | Moderate/  severe | High | Low |
| Cholinesterase Inhibitor + Memantine vs. Placebo [84] | 1 (146) | 70-79.9 | ≥50% | Clinic | Not specified | >30 | AD | Moderate/  severe | High | Low |
| Dextromethorphan-Quinidine vs. Placebo [41] | 1 (220) | 70-79.9 | ≥50% | Specialty clinic | Not specified | <13 | AD | Mild/  moderate/  severe | High | Low |
| Memantine vs. Antipsychotics [16] | 1 (199) | ≥80 | ≥50% | LTC | Not specified | 13-30 | AD | Not specified | High | Low |
| Memantine vs. Placebo [5, 12, 51, 54, 74, 78, 84, 157, 193, 195, 199] | 11 (3970) | 70-79.9 | ≥50% | Clinic | Not specified | >30 | AD | Mild/  moderate | High | Low |

Abbreviations: long-term care (LTC), Alzheimer’s disease (AD), number (No.), risk of bias (RoB)

# Table 4d. Assessment of Transitivity: RCTs Reporting Falls

| **Treatment Comparison** | **No. of Studies (No. of Patients)** | **Age (years)** | **Sex** | **Study Setting** | **Study Duration (weeks)** | **Type of Dementia** | **Severity of Dementia** | **RoB: Missing Data** | **RoB: Randomization Procedure** |
| --- | --- | --- | --- | --- | --- | --- | --- | --- | --- |
| Anticonvulsants vs. Placebo [132, 135, 164, 176, 181, 183] | 6 (696) | ≥80 | ≥50% | LTC/assisted living | >30 | Multiple | Moderate | High | Unclear |
| Antidepressants vs. Placebo [35, 45, 109, 150] | 4 (280) | 70-79.9 | ≥50% | Clinic/community | 13-30 | AD | Mild/  moderate | High | Low |
| Antipsychotics vs. Antidepresants [19, 184] | 2 (95) | ≥80 | ≥50% | Hospital | <13 | AD/multiple | Mild/  moderate/  severe | High | Unclear |
| Antipsychotics vs. Placebo [17, 24, 44, 46, 47, 93, 115, 128, 139, 182, 208] | 11 (3214) | ≥80 | ≥50% | LTC/assisted living | <13 | Multiple | Mild/  moderate/  severe | High | Unclear |
| Cholinesterase Inhibitors vs. Memantine [84] | 1 (149) | 70-79.9 | ≥50% | Clinic | >30 | AD | Moderate/  severe | High | Low |
| Cholinesterase Inhibitor vs. Placebo [10, 15, 25, 29, 50, 53, 68, 75, 79, 82-84, 177, 192, 202] | 15 (9447) | 70-79.9 | ≥50% | Clinic/community/  LTC/assisted living | >30 | AD | Mild/  moderate | High | Low |
| Cholinesterase Inhibitor + Memantine vs. Memantine [84] | 1 (149) | 70-79.9 | ≥50% | Clinic | >30 | AD | Moderate/  severe | High | Low |
| Cholinesterase Inhibitor + Memantine vs. Cholinesterase Inhibitor [49, 57, 84, 130, 133] | 5 (2500) | 70-79.9 | ≥50% | Clinic/community | >30 | AD | Mild/  moderate | High | Low |
| Cholinesterase Inhibitor + Memantine vs. Placebo [84] | 1 (146) | 70-79.9 | ≥50% | Clinic | >30 | AD | Moderate/  severe | High | Low |
| Dextromethorphan-Quinidine vs. Placebo [41] | 1 (220) | 70-79.9 | ≥50% | Clinic/LTC/  assisted living | <13 | AD | Mild/  moderate/ severe | High | Low |
| Memantine vs. Placebo [12, 51, 54, 74, 78, 84, 123, 129, 141, 157, 180, 187, 188, 193, 199, 218] | 16 (5470) | 70-79.9 | ≥50% | Clinic | >30 | AD | Moderate/  severe | High | Low |

Abbreviations: long-term care (LTC), Alzheimer’s disease (AD), number (No.), risk of bias (RoB)

# Table 5. Risk of Bias Assessment for Each RCT with the Cochrane Risk of Bias Tool[219]

| **Author, Year** | **Random sequence generation** | **Allocation concealment** | **Blinding of participants and personnel** | **Blinding of outcome assessment** | **Incomplete outcome data** | **Selective reporting** | **Other sources of bias** |
| --- | --- | --- | --- | --- | --- | --- | --- |
| Aarsland, 2009[5] | Low risk | Unclear risk | Low risk | Low risk | High risk | Low risk | Low risk |
| Allain, 2000[8] | Unclear risk | Unclear risk | Low risk | Low risk | High risk | Unclear risk | Unclear risk |
| Ancoli-Israel, 2005[9] | Unclear risk | Unclear risk | Low risk | Low risk | Low risk | Low risk | High risk |
| Auchus, 2007[10] | Unclear risk | Unclear risk | Low risk | Low risk | High risk | Unclear risk | Unclear risk |
| Bakchine, 2007[12] | Unclear risk | Low risk | Low risk | Low risk | High risk | Unclear risk | High risk |
| Ballard, 2004[13] | Unclear risk | Unclear risk | Low risk | Low risk | High risk | Unclear risk | Low risk |
| Ballard, 2005[14] | Low risk | Low risk | Low risk | Low risk | High risk | Unclear risk | Low risk |
| Ballard, 2008a[15] | Low risk | Low risk | Low risk | Low risk | High risk | Low risk | High risk |
| Ballard, 2008b[210] | Low risk | Low risk | Low risk | Low risk | High risk | Low risk | Low risk |
| Ballard, 2015[16] | Low risk | Low risk | Low risk | Low risk | High risk | Low risk | High risk |
| Ballard, 2018[17] | Low risk | Low risk | Low risk | Low risk | High risk | Low risk | High risk |
| Banerjee, 2011[18] | Low risk | Low risk | Low risk | Low risk | High risk | Low risk | Low risk |
| Barak, 2011[19] | Unclear risk | Unclear risk | Low risk | Low risk | High risk | Low risk | High risk |
| Baxer, 2013[21] | Low risk | Low risk | Low risk | Low risk | Low risk | Low risk | Low risk |
| Black, 2003[22] | Low risk | Unclear risk | Low risk | Low risk | Low risk | Unclear risk | High risk |
| Black, 2007[23] | Low risk | Unclear risk | Low risk | Low risk | High risk | Low risk | High risk |
| Brodaty, 2003[24] | Low risk | Unclear risk | Low risk | Low risk | High risk | Unclear risk | High risk |
| Brodaty, 2005[25] | Low risk | Unclear risk | Low risk | Low risk | High risk | Unclear risk | High risk |
| Bullock, 2005[27] | Low risk | Low risk | Low risk | Low risk | Low risk | Unclear risk | High risk |
| Burns, 1999[28] | Unclear risk | Unclear risk | Low risk | Low risk | Low risk | High risk | High risk |
| Burns, 2009[29] | Low risk | Low risk | Low risk | Low risk | High risk | High risk | High risk |
| Camargos, 2014[30] | Low risk | Low risk | Low risk | Low risk | High risk | Low risk | Low risk |
| Campbell, 2017[31] | Low risk | Unclear risk | High risk | High risk | High risk | High risk | Low risk |
| Carlyle, 1993[32] | Unclear risk | Unclear risk | High risk | Low risk | High risk | Unclear risk | Unclear risk |
| Chan, 2001[33] | Unclear risk | Unclear risk | Low risk | Low risk | Low risk | Unclear risk | High risk |
| Choe, 2016[35] | Low risk | Low risk | Low risk | Low risk | High risk | Unclear risk | Low risk |
| Choi, 2011[36] | Low risk | Unclear risk | Low risk | Low risk | Low risk | Low risk | Unclear risk |
| Corey-Bloom, 1998[37] | Unclear risk | Low risk | Low risk | Low risk | High risk | Unclear risk | High risk |
| AD2000 Collaborative Group, 2004[6] | Low risk | Low risk | Low risk | Unclear risk | Low risk | Unclear risk | Low risk |
| Culo, 2010[38] | Unclear risk | Unclear risk | Low risk | Low risk | Low risk | Low risk | Low risk |
| Cumbo, 2014[39] | Unclear risk | Unclear risk | High risk | Low risk | High risk | Unclear risk | Low risk |
| Cummings, 2012[40] | Low risk | Low risk | Low risk | Low risk | High risk | High risk | High risk |
| Cummings, 2015[41] | Low risk | Unclear risk | Low risk | Low risk | High risk | Low risk | High risk |
| De Deyn, 1999[42] | Low risk | Unclear risk | Low risk | Low risk | High risk | High risk | Unclear risk |
| De Deyn, 2004[43] | Unclear risk | Unclear risk | Low risk | Low risk | High risk | Unclear risk | High risk |
| De Deyn, 2005[44] | Unclear risk | Unclear risk | Low risk | Low risk | High risk | High risk | High risk |
| de Vasconcelos Cunha, 2007[45] | Unclear risk | Unclear risk | Low risk | Low risk | High risk | Unclear risk | Unclear risk |
| Deberdt, 2005[46] | Unclear risk | Unclear risk | Low risk | Low risk | High risk | Unclear risk | High risk |
| Devanand, 2012[47] | Low risk | Low risk | Low risk | Low risk | Low risk | Low risk | Low risk |
| Doody, 2012[49] | Low risk | Unclear risk | Low risk | Low risk | High risk | Unclear risk | High risk |
| Dubois, 2012[50] | Low risk | Unclear risk | Low risk | Low risk | High risk | Unclear risk | High risk |
| Dysken, 2014[51] | Low risk | Low risk | Low risk | Low risk | High risk | High risk | Low risk |
| Emre, 2004[53] | Low risk | Low risk | Low risk | Low risk | Low risk | Unclear risk | High risk |
| Emre, 2010[54] | Low risk | Low risk | Low risk | Low risk | Low risk | Low risk | High risk |
| Emre, 2014[55] | Low risk | Unclear risk | High risk | High risk | High risk | High risk | High risk |
| Erkinjuntti, 2002[56] | Low risk | Unclear risk | Low risk | Low risk | High risk | High risk | High risk |
| Farlow, 2010[57] | Unclear risk | High risk | High risk | High risk | High risk | Low risk | High risk |
| Farlow, 2013[58] | Low risk | Low risk | Low risk | Low risk | Low risk | High risk | High risk |
| Feldman, 2001[59] | Low risk | Unclear risk | Low risk | Low risk | High risk | Unclear risk | High risk |
| Feldman, 2007[60] | Unclear risk | Unclear risk | Low risk | Low risk | High risk | Unclear risk | Unclear risk |
| Finkel, 1995[61] | Unclear risk | Unclear risk | Low risk | Low risk | High risk | Unclear risk | Unclear risk |
| Fontaine, 2003[63] | Unclear risk | Unclear risk | Low risk | Low risk | Low risk | Unclear risk | High risk |
| Fox, 2012[64] | Low risk | Unclear risk | Low risk | Low risk | High risk | High risk | High risk |
| Gault, 2016[68] | Low risk | Unclear risk | Low risk | Low risk | High risk | Low risk | High risk |
| Gold, 2010[73] | Low risk | Unclear risk | Low risk | Low risk | High risk | High risk | High risk |
| Grossberg, 2013[74] | Low risk | Low risk | Low risk | Low risk | High risk | High risk | High risk |
| Hager, 2014[75] | Low risk | Low risk | Low risk | Low risk | High risk | High risk | High risk |
| Hampel, 2009[76] | Low risk | Unclear risk | Low risk | High risk | High risk | Low risk | High risk |
| Herrmann, 2007[77] | Unclear risk | Unclear risk | Unclear risk | Unclear risk | Low risk | Unclear risk | Low risk |
| Herrmann, 2013[78] | Low risk | Unclear risk | Low risk | Low risk | High risk | High risk | High risk |
| Herrmann, 2016[79] | Low risk | Unclear risk | Low risk | Low risk | Low risk | Low risk | Low risk |
| Homma, 1998[80] | Low risk | Low risk | Low risk | Low risk | High risk | Unclear risk | Unclear risk |
| Homma, 2000[81] | Unclear risk | Unclear risk | Low risk | Low risk | Low risk | Unclear risk | Unclear risk |
| Homma, 2008[82] | Low risk | Low risk | Low risk | Low risk | Low risk | Unclear risk | High risk |
| Howard, 2007[83] | Low risk | Low risk | Low risk | Low risk | High risk | Low risk | High risk |
| Howard, 2012[84] | Low risk | Low risk | Low risk | Low risk | High risk | Low risk | Unclear risk |
| Hu, 2006[85] | Low risk | Unclear risk | High risk | High risk | Low risk | Unclear risk | Unclear risk |
| Jia, 2017[88] | Low risk | Low risk | Low risk | Unclear risk | Low risk | Unclear risk | Unclear risk |
| Johannsen, 2006[89] | Low risk | Unclear risk | Low risk | Low risk | Low risk | Unclear risk | High risk |
| Katona, 1998[92] | Unclear risk | Unclear risk | Low risk | Low risk | High risk | Unclear risk | Unclear risk |
| Katz, 1999[93] | Low risk | Unclear risk | Low risk | Low risk | High risk | Unclear risk | High risk |
| Katz, 2004[94] | Low risk | Unclear risk | Low risk | Low risk | High risk | Unclear risk | High risk |
| Kennedy, 2005[95] | Unclear risk | Unclear risk | Low risk | Low risk | High risk | Unclear risk | High risk |
| Kertez, 2008[96] | Low risk | Low risk | Low risk | Low risk | Low risk | Low risk | High risk |
| Lanctot, 2002[98] | Unclear risk | Unclear risk | Low risk | Low risk | Low risk | Unclear risk | Unclear risk |
| Litvinenko, 2008[105] | Unclear risk | Unclear risk | High risk | High risk | Low risk | Unclear risk | Unclear risk |
| Lyketsos, 2003[109] | Low risk | Low risk | Low risk | Low risk | High risk | Unclear risk | Low risk |
| Maher-Edwards, 2011[110] | Low risk | Unclear risk | Low risk | Low risk | Low risk | Low risk | High risk |
| McKeith, 2000[114] | Low risk | Low risk | Low risk | Low risk | High risk | Unclear risk | High risk |
| Mintzer, 2006[115] | Low risk | Low risk | Low risk | Low risk | High risk | Unclear risk | Unclear risk |
| Mintzer, 2007[116] | Unclear risk | Unclear risk | Low risk | Low risk | High risk | Unclear risk | High risk |
| Mohs, 2001[117] | Unclear risk | Unclear risk | Low risk | Low risk | High risk | Unclear risk | High risk |
| Mok, 2007[118] | Low risk | Unclear risk | Low risk | Low risk | High risk | Unclear risk | Unclear risk |
| Moretti, 2005[119] | Unclear risk | Unclear risk | High risk | High risk | Low risk | Unclear risk | Unclear risk |
| Nakamura, 2011[122] | Low risk | Low risk | Low risk | Low risk | High risk | High risk | High risk |
| Nyth, 1990[125] | Unclear risk | Unclear risk | Low risk | Low risk | High risk | Unclear risk | Unclear risk |
| Orgogozo, 2002[126] | Unclear risk | Unclear risk | Low risk | Low risk | Low risk | Unclear risk | High risk |
| Pakdaman, 2015[127] | Low risk | Unclear risk | High risk | High risk | High risk | Unclear risk | Unclear risk |
| Paleacu, 2008[128] | Unclear risk | Unclear risk | Low risk | Low risk | High risk | Unclear risk | High risk |
| Peskind, 2006[129] | Low risk | Low risk | Low risk | Low risk | High risk | Unclear risk | High risk |
| Peters, 2015[130] | Unclear risk | Unclear risk | Low risk | Low risk | High risk | Low risk | Unclear risk |
| Porsteinsson, 2001[132] | Unclear risk | Unclear risk | High risk | Low risk | High risk | Unclear risk | Unclear risk |
| Porsteinsson, 2008[133] | Low risk | Unclear risk | Low risk | Low risk | Low risk | Unclear risk | High risk |
| Porsteinsson, 2014[134] | Low risk | Low risk | Low risk | Low risk | High risk | Low risk | Low risk |
| Profenno, 2005[135] | Low risk | Unclear risk | Low risk | Low risk | High risk | Unclear risk | High risk |
| Rainer, 2007[137] | Low risk | Low risk | High risk | Low risk | Low risk | Unclear risk | High risk |
| Raskind, 2000[140] | Low risk | Unclear risk | Low risk | Low risk | High risk | Low risk | High risk |
| Rappaport, 2009[139] | Unclear risk | Unclear risk | Low risk | Low risk | Low risk | High risk | High risk |
| Reisberg, 2003[141] | Low risk | Low risk | Low risk | Low risk | High risk | Unclear risk | High risk |
| Rockwood, 2001[146] | Low risk | Low risk | Low risk | Low risk | High risk | Low risk | High risk |
| Rogers, 1998a[147] | Unclear risk | Unclear risk | Low risk | Low risk | Low risk | Unclear risk | High risk |
| Rogers, 1998b[148] | Low risk | Unclear risk | Low risk | Low risk | High risk | Unclear risk | Unclear risk |
| Roman, 2010[149] | Unclear risk | Unclear risk | Low risk | Low risk | High risk | Low risk | High risk |
| Rosenberg, 2010[150] | Low risk | Unclear risk | Low risk | Unclear risk | High risk | Unclear risk | Low risk |
| Rosenberg, 2013[151] | Low risk | Low risk | Low risk | Low risk | Low risk | Low risk | Low risk |
| Rosler, 1999[152] | Low risk | Unclear risk | Low risk | Low risk | Low risk | Unclear risk | High risk |
| Ruths, 2008[154] | Low risk | Unclear risk | Low risk | Unclear risk | Low risk | Unclear risk | Unclear risk |
| Saxton, 2012[157] | Low risk | Low risk | Low risk | Low risk | Low risk | High risk | High risk |
| Scarpini, 2011[158] | Low risk | Unclear risk | Low risk | Low risk | High risk | Unclear risk | High risk |
| Schneider, 2006[160] | Low risk | Low risk | Low risk | Low risk | High risk | Unclear risk | Low risk |
| Sival, 2002[163] | Unclear risk | Unclear risk | Low risk | Low risk | Low risk | Unclear risk | Low risk |
| Sommer, 2009[164] | Unclear risk | Unclear risk | Low risk | Low risk | High risk | Low risk | Low risk |
| Street, 2000[166] | Low risk | Unclear risk | Low risk | Low risk | High risk | Unclear risk | High risk |
| Streim, 2008[167] | Unclear risk | Unclear risk | Low risk | Low risk | Low risk | High risk | High risk |
| Tariot, 1998[176] | Unclear risk | Unclear risk | High risk | Low risk | Low risk | Unclear risk | Low risk |
| Tariot, 2000[177] | Low risk | Unclear risk | Low risk | Low risk | High risk | Unclear risk | High risk |
| Tariot, 2001a[178] | Low risk | Unclear risk | Low risk | Low risk | Low risk | Unclear risk | High risk |
| Tariot, 2001b[179] | Unclear risk | Unclear risk | Low risk | Low risk | High risk | Unclear risk | Unclear risk |
| Tariot, 2004[180] | Low risk | Low risk | Low risk | Low risk | High risk | Unclear risk | High risk |
| Tariot, 2005[181] | Low risk | Low risk | Low risk | Low risk | High risk | Unclear risk | High risk |
| Tariot, 2006[182] | Unclear risk | Unclear risk | Low risk | Low risk | High risk | Unclear risk | High risk |
| Tariot, 2011[183] | Low risk | Unclear risk | Low risk | Low risk | High risk | Low risk | High risk |
| Teranishi, 2013[184] | Low risk | Unclear risk | High risk | Low risk | Low risk | High risk | Unclear risk |
| van Dyck, 2007[187] | Unclear risk | Unclear risk | Low risk | Low risk | Low risk | Unclear risk | High risk |
| Vercelletto, 2011[188] | Low risk | Low risk | Low risk | Low risk | High risk | Low risk | Low risk |
| Wilcock, 2000[192] | Low risk | Low risk | Low risk | Low risk | High risk | High risk | Unclear risk |
| Wilcock, 2002[193] | Low risk | Low risk | Low risk | Low risk | Low risk | Unclear risk | High risk |
| Wilcock, 2003[194] | Low risk | Unclear risk | Unclear risk | Low risk | High risk | Unclear risk | High risk |
| Wilkinson, 2001[196] | Low risk | Low risk | Low risk | Low risk | High risk | High risk | High risk |
| Wilkinson, 2002[197] | Unclear risk | Unclear risk | Unclear risk | Low risk | High risk | Unclear risk | Unclear risk |
| Wilkinson, 2003[198] | Low risk | Unclear risk | Low risk | Low risk | Low risk | Unclear risk | High risk |
| Wilkinson, 2012[199] | Low risk | Low risk | Low risk | Low risk | High risk | High risk | Unclear risk |
| Winblad, 1999[200] | Unclear risk | Unclear risk | Low risk | Low risk | Low risk | Unclear risk | Unclear risk |
| Winblad, 2001[201] | Low risk | Unclear risk | Low risk | Low risk | High risk | Unclear risk | Unclear risk |
| Winblad, 2006[202] | Low risk | Unclear risk | Low risk | Low risk | High risk | Unclear risk | High risk |
| Winblad, 2007[203] | Low risk | Low risk | Low risk | Low risk | High risk | Unclear risk | High risk |
| Zhang, 2012[207] | Unclear risk | Unclear risk | Low risk | Low risk | High risk | Unclear risk | High risk |
| Zhong, 2007[208] | Unclear risk | Low risk | Low risk | Low risk | High risk | Unclear risk | High risk |

# Table 6. Risk of Bias Assessment for Each Cohort Study with the Newcastle-Ottawa Quality Assessment Scale[220]

| **Author, Year** | **Representative-ness of Exposed Cohort** | **Selection of Non-Exposed Cohort** | **Ascertainment of Exposure** | **Demonstration that Outcome of Interest Not Present at Start of Study** | **Comparability of Cohorts: Design or Analysis** | **Assessment of Outcome** | **Follow-Up Long Enough for Outcomes to Occur** | **Adequacy of Cohort Follow-Up** |
| --- | --- | --- | --- | --- | --- | --- | --- | --- |
| Aguglia, 2004[7] | A | A | A | A | C | D | A | A |
| Barnett, 2007[20] | B | A | A | A | A | A | A | A |
| Bronskill, 2018[26] | A | A | A | A | B | A | A | A |
| Chan, 2010[34] | B | A | A | A | A | A | A | A |
| Eady, 2018[52] | B | A | A | A | A | A | A | A |
| Finkel, 2005[62] | A | A | A | A | A | A | A | A |
| Gasper, 2004[67] | A | A | A | A | B | B | A | A |
| Gerhard, 2014[69] | A | A | A | A | A | A | A | B |
| Gill, 2005[70] | A | A | A | A | A | A | A | A |
| Gill, 2007[71] | A | A | A | A | A | A | A | A |
| Gill, 2009[72] | A | A | A | A | A | A | A | A |
| Huybrechts, 2012[86] | A | A | A | A | A | A | A | A |
| Kales, 2007[90] | B | A | A | A | A | A | A | A |
| Kales, 2012[91] | A | A | A | A | A | A | A | A |
| Kheirbek, 2019[97] | B | A | A | A | C | A | A | A |
| Langballe, 2014[99] | B | A | A | A | A | A | A | C |
| Layton, 2005[100] | A | A | A | A | A | A | A | A |
| Lee, 2017[101] | B | A | A | A | C | A | B | A |
| Lin, 2016[102] | A | A | A | A | A | A | A | A |
| Liperoti, 2009[104] | B | A | A | A | B | A | A | A |
| Lopez, 1999[106] | A | A | A | A | B | A | A | A |
| Lopez, 2009[107] | A | A | A | A | B | B | A | A |
| Lopez-Pousa, 2006[108] | B | A | A | A | B | A | A | B |
| Martin, 2003[111] | B | A | A | A | B | A | A | A |
| Martinez Martinez, 2009[112] | A | A | A | A | A | A | A | A |
| Mueller, 2017[120] | A | A | A | A | B | A | A | A |
| Musicco, 2011[121] | C | A | A | A | A | A | A | A |
| Nordstrom, 2013[124] | B | A | A | A | B | A | A | A |
| Piersanti, 2014[131] | A | A | A | A | A | B | A | A |
| Rafaniello, 2014[136] | B | A | A | A | B | B | A | A |
| Raivio, 2007[138] | B | A | A | A | B | A | A | A |
| Rochon, 2008[145] | A | A | A | A | A | A | A | A |
| Rossom, 2010[153] | C | A | A | A | A | A | A | A |
| Sahlberg, 2015[155] | A | A | A | A | A | B | A | A |
| Santos-Garcia, 2010[156] | B | A | A | A | C | A | A | A |
| Schneeweiss, 2007[159] | A | A | A | A | A | A | A | A |
| Shin, 2015[161] | A | A | A | A | A | A | A | A |
| Sinforiani, 2011[162] | B | A | A | A | B | B | A | A |
| Sterke, 2012[165] | B | A | A | A | B | A | A | A |
| Sturm, 2018[168] | B | A | A | A | C | A | A | A |
| Su, 2019[169] | A | A | A | A | A | A | A | A |
| Suh, 2005[170] | B | A | A | A | B | B | A | A |
| Sultana, 2014[171] | A | A | A | A | B | B | A | A |
| Tan, 2018[174] | A | B | A | A | A | A | A | A |
| Trifiro, 2007[185] | A | A | A | A | A | A | A | A |
| Torstensson, 2017[186] | A | A | A | A | A | A | A | A |
| Wang, 2005[189] | A | A | A | A | A | A | B | A |
| Watt, 2018[190] | A | A | A | A | B | A | A | A |
| Wei, 2017[191] | A | A | A | A | B | A | A | A |
| Wu, 2015[204] | B | A | A | A | A | A | A | A |
| Wysowski, 1996[205] | A | A | A | A | B | A | A | A |
| Yin, 2015[206] | B | A | A | A | C | A | A | D |
| Zhu, 2013[209] | B | A | A | A | B | A | A | A |

Note: letters earlier in the alphabet denote an item is at lower risk of bias, except for the item ‘comparability of cohorts on the basis of the design or analysis’ where ‘B’ implies the cohorts are more comparable than ‘A’ (i.e. controlled for a geriatric syndrome such as frailty, falls risk, degree or cognitive or functional impairment) and ‘C’ implies there was no adjustment for possible confounders.

# Table 7. Risk of Bias Assessment for Each Case-Control Study with the Newcastle-Ottawa Quality Assessment Scale [220]

| **Author, Year** | **Adequate Case Definition** | **Representativeness of Cases** | **Selection of Controls** | **Definition of Controls** | **Comparability of Cases and Controls** | **Assessment of Exposure** | **Method of Ascertainment for Cases and Controls** |
| --- | --- | --- | --- | --- | --- | --- | --- |
| Jalbert, 2010[87] | B | A | A | A | B | A | A |
| Liperoti, 2005[103] | B | A | A | A | B | A | A |
| Maust, 2015[113] | B | A | A | A | A | A | A |
| Tamimi, 2012[172] | B | A | A | A | B | A | A |
| Tamimi, 2017[173] | B | A | A | A | B | A | A |

Note: letters earlier in the alphabet denote an item is at lower risk of bias, except for the item ‘comparability of cohorts on the basis of the design or analysis’ where ‘B’ implies the cohorts are more comparable than ‘A’ (i.e. controlled for a geriatric syndrome such as frailty, falls risk, degree or cognitive or functional impairment) and ‘C’ implies there was no adjustment for possible confounders.

# Table 8. Risk of Bias Assessment for Each ‘Other’ Non-Randomized Study with the Cochrane Effective Practice and Organization of Care Tool [221]

| **Author, Year** | **Random Sequence Generation** | **Allocation Concealment** | **Similar Baseline Outcome Measures** | **Similar Baseline Characteristics** | **Incomplete Outcome Data** | **Blinding** | **Contamination** | **Selective Outcome Reporting** | **Other Bias** |
| --- | --- | --- | --- | --- | --- | --- | --- | --- | --- |
| Tariot, 1995[175] | High risk | High risk | Low risk | Unclear risk | Low risk | Low risk | Low risk | Unclear risk | Unclear risk |

# Figure 1. Summary of Risk of Bias Assessment for RCTs

# Table 9a. Bayesian Pairwise and Network Meta-Analysis: RCTs Reporting Fractures

| **Comparison** | **NMA OR estimate** | **95% CrI** | **95% PrI** | **MA OR estimate** | **95% CrI** |
| --- | --- | --- | --- | --- | --- |
| Antidepressants vs Placebo | 0.16 | <0.01 to 5.34 | <0.01 to 5.5 | 0.16 | <0.01 to 4.57 |
| Anticonvulsants vs Placebo | 0.11 | <0.01 to 2.10 | <0.01 to 2.19 | 0.11 | <0.01 to 2.15 |
| Memantine vs Placebo | 0.58 | 0.16 to 1.93 | 0.14 to 2.09 | 0.59 | 0.15 to 1.92 |
| Cholinesterase Inhibitors vs Placebo | 1.34 | 0.86 to 2.14 | 0.71 to 2.58 | 1.34 | 0.86 to 2.15 |
| Antipsychotics vs Placebo | 0.72 | 0.33 to 1.66 | 0.29 to 1.88 | 0.72 | 0.33 to 1.69 |
| Cholinesterase Inhibitor + Memantine vs Placebo | 6.63 | 0.75 to 209.7 | 0.72 to 214.1 | - | - |
| Anticonvulsants vs Antidepressants | 0.64 | <0.01 to 544.8 | <0.01 to 547.7 | - | - |
| Memantine vs Antidepressants | 3.58 | 0.08 to 1668 | 0.08 to 1695 | - | - |
| Cholinesterase Inhibitors vs Antidepressants | 8.19 | 0.24 to 3618 | 0.24 to 3690 | - | - |
| Antipsychotics vs Antidepressants | 4.46 | 0.12 to 2007 | 0.12 to 2039 | - | - |
| Cholinesterase Inhibitor + Memantine vs Antidepressants | 48.71 | 0.65 to 36400 | 0.63 to 36790 | - | - |
| Memantine vs Anticonvulsants | 5.30 | 0.21 to 2338 | 0.2 to 2374 | - | - |
| Cholinesterase Inhibitors vs Anticonvulsants | 11.99 | 0.62 to 5246 | 0.59 to 5310 | - | - |
| Antipsychotics vs Anticonvulsants | 6.59 | 0.3 to 2767 | 0.29 to 2816 | - | - |
| Cholinesterase Inhibitor + Memantine vs Anticonvulsants | 74.46 | 1.43 to 47880 | 1.4 to 48760 | - | - |
| Cholinesterase Inhibitors vs Memantine | 2.33 | 0.64 to 9.28 | 0.6 to 9.93 | - | - |
| Antipsychotics vs Memantine | 1.26 | 0.3 to 5.82 | 0.28 to 6.24 | - | - |
| Cholinesterase Inhibitor + Memantine vs Memantine | 11.93 | 0.94 to 450.7 | 0.91 to 462.8 | - | - |
| Antipsychotics vs Cholinesterase Inhibitors | 0.54 | 0.21 to 1.38 | 0.19 to 1.54 | - | - |
| Cholinesterase Inhibitor + Memantine vs Cholinesterase Inhibitors | 4.92 | 0.59 to 152.5 | 0.56 to 156.2 | 5.29 | 0.62 to 136.4 |
| Cholinesterase Inhibitor + Memantine vs Antipsychotics | 9.20 | 0.87 to 315 | 0.83 to 324.6 | - | - |
| *Common within-network between-study heterogeneity variance* | *0.02* | *<0.01 to 0.27* |  | *0.02* | *<0.01 to 0.28* |
| *Consistency NMA model:*  *DIC statistic 91, residual deviance 52.8, # of data points 58, effective number of parameters 38.2* | | | | | |

Abbreviations: network meta-analysis (NMA), credible interval (CrI), predictive interval (PrI), meta-analysis (MA), odds ratio (OR), deviance information criterion (DIC)

# Table 9b. Frequentist Pairwise and Network Meta-Analysis: RCTs Reporting Fractures

| **Comparison** | **NMA OR estimate** | **95% CI** | **MA OR estimate** | **95% CI** |
| --- | --- | --- | --- | --- |
| Antidepressants vs Placebo | 0.28 | 0.01 to 7.4 | 0.28 | 0.01 to 7.4 |
| Anticonvulsants vs Placebo | 0.21 | 0.01 to 4.34 | 0.21 | 0.01 to 4.34 |
| Memantine vs Placebo | 0.64 | 0.16 to 2.64 | 0.64 | 0.16 to 2.64 |
| Cholinesterase Inhibitors vs Placebo | 1.29 | 0.81 to 2.04 | 1.29 | 0.81 to 2.04 |
| Antipsychotics vs Placebo | 0.64 | 0.3 to 1.37 | 0.64 | 0.3 to 1.37 |
| Cholinesterase Inhibitor + Memantine vs Placebo | 4.84 | 0.51 to 46.35 | - | - |
| Anticonvulsants vs Antidepressants | 0.73 | 0.01 to 63.69 | - | - |
| Memantine vs Antidepressants | 2.28 | 0.07 to 80.22 | - | - |
| Cholinesterase Inhibitors vs Antidepressants | 4.56 | 0.17 to 123.58 | - | - |
| Antipsychotics vs Antidepressants | 2.27 | 0.08 to 65.04 | - | - |
| Cholinesterase Inhibitor + Memantine vs Antidepressants | 17.17 | 0.32 to 911.42 | - | - |
| Memantine vs Anticonvulsants | 3.12 | 0.11 to 89.41 | - | - |
| Cholinesterase Inhibitors vs Anticonvulsants | 6.23 | 0.29 to 135.41 | - | - |
| Antipsychotics vs Anticonvulsants | 3.10 | 0.13 to 71.55 | - | - |
| Cholinesterase Inhibitor + Memantine vs Anticonvulsants | 23.45 | 0.53 to 1038.71 | - | - |
| Cholinesterase Inhibitors vs Memantine | 2.00 | 0.45 to 8.81 | - | - |
| Antipsychotics vs Memantine | 0.99 | 0.2 to 4.94 | - | - |
| Cholinesterase Inhibitor + Memantine vs Memantine | 7.52 | 0.52 to 107.83 | - | - |
| Antipsychotics vs Cholinesterase Inhibitors | 0.50 | 0.2 to 1.21 | - | - |
| Cholinesterase Inhibitor + Memantine vs Cholinesterase Inhibitors | 3.76 | 0.41 to 34.36 | 3.76 | 0.41 to 34.36 |
| Cholinesterase Inhibitor + Memantine vs Antipsychotics | 7.56 | 0.7 to 81.95 | - | - |
| *Common within-network between-study heterogeneity variance* | <0.01 | - | *-* | *-* |
| *Design-by-treatment interaction model for inconsistency χ^2^(d.f., P-value, heterogeneity variance)* | N/A | - | *-* | *-* |

Abbreviations: network meta-analysis (NMA), confidence interval (CI), meta-analysis (MA), odds ratio (OR), not applicable (N/A)

# Table 9c. Bayesian Network Meta-Analysis: RCTs + NRSs Reporting Fractures

| **Comparison** | **Combined RCT+NRS estimate (RR)** | **95% CrI** | **RCT estimate (RR)** | **95% CrI** | **NRS estimate (RR)** | **95% CrI** |
| --- | --- | --- | --- | --- | --- | --- |
| Antidepressants vs. Placebo | 0.82 | 0.16 to 3.24 | 0.74 | 0.12 to 3.11 | 0.94 | 0.25 to 2.39 |
| Antipsychotics vs. Placebo | 0.89 | 0.29 to 2.68 | 0.77 | 0.36 to 1.58 | 1.05 | 0.41 to 2.25 |
| Cholinesterase Inhibitors vs. Placebo | 1.02 | 0.36 to 2.76 | 1.23 | 0.81 to 1.91 | 0.84 | 0.48 to 1.52 |
| Memantine vs. Placebo | 0.59 | 0.1 to 3.38 | 0.59 | 0.17 to 1.93 | - | - |
| Anticonvulsants vs. Placebo | 0.63 | 0.05 to 6.54 | 0.63 | 0.08 to 4.33 | - | - |
| Cholinesterase Inhibitor + Memantine vs. Placebo | 6.18 | 0.5 to 325.1 | 6.1 | 0.7 to 294.8 | - | - |
| Antipsychotics vs. Antidepressants | 1.11 | 0.22 to 6.46 | 1.08 | 0.21 to 6.29 | 1.13 | 0.56 to 2.59 |
| Cholinesterase Inhibitors vs. Antidepressants | 1.25 | 0.23 to 8.76 | 1.64 | 0.39 to 11.87 | 0.89 | 0.3 to 4.13 |
| Memantine vs. Antidepressants | 0.74 | 0.08 to 7.88 | 0.82 | 0.12 to 7.08 | - | - |
| Anticonvulsants vs. Antidepressants | 0.80 | 0.04 to 12.56 | 0.91 | 0.07 to 11.07 | - | - |
| Cholinesterase Inhibitor + Memantine vs. Antidepressants | 7.98 | 0.45 to 479.3 | 8.98 | 0.61 to 450.8 | - | - |
| Cholinesterase Inhibitor vs. Antipsychotics | 1.14 | 0.25 to 5.17 | 1.6 | 0.68 to 3.94 | 0.8 | 0.3 to 2.65 |
| Memantine vs. Antipsychotics | 0.66 | 0.08 to 5.19 | 0.77 | 0.18 to 3.07 | - | - |
| Anticonvulsant vs. Antipsychotics | 0.71 | 0.05 to 9.13 | 0.82 | 0.08 to 6.42 | - | - |
| Cholinesterase Inhibitor + Memantine vs. Antipsychotics | 7.08 | 0.45 to 398.6 | 8.1 | 0.77 to 373.4 | - | - |
| Memantine vs. Cholinesterase Inhibitor | 0.58 | 0.08 to 4.38 | 0.49 | 0.13 to 1.67 | - | - |
| Anticonvulsant vs. Cholinesterase Inhibitor | 0.62 | 0.04 to 7.89 | 0.51 | 0.06 to 3.81 | - | - |
| Cholinesterase Inhibitor + Memantine vs. Cholinesterase Inhibitors | 6.04 | 0.45 to 338.5 | 4.89 | 0.61 to 230.7 | - | - |
| Anticonvulsants vs. Memantine | 1.06 | 0.05 to 20.55 | 1.06 | 0.09 to 11.4 | - | - |
| Cholinesterase Inhibitor + Memantine vs. Memantine | 10.86 | 0.49 to 748.1 | 10.78 | 0.83 to 505.7 | - | - |
| Cholinesterase Inhibitor + Memantine vs. Anticonvulsants | 10.61 | 0.33 to 872.4 | 10.59 | 0.54 to 602.9 | - | - |
| *Between-study type heterogeneity variance* | *0.18 (95% CrI <0.01 to 2.19)* | | | | | |
| *Between-RCT heterogeneity variance* | *0.02 (95% CrI <0.01 to 0.27)* | | | | | |
| *Between-NRS heterogeneity variance* | *0.16 (0.02 to 1.32)* | | | | | |

Abbreviations: credible interval (CrI), network meta-analysis (NMA), nonrandomized study (NRS), randomized trial (RCT), relative risk (RR)

# Table 9d. Bayesian Pairwise and Network Meta-Analysis: RCTs Reporting Mortality

| **Comparison** | **NMA OR estimate** | **95% CrI** | **95% PrI** | **MA OR estimate** | **95% CrI** |
| --- | --- | --- | --- | --- | --- |
| Antidepressants vs Placebo | 0.83 | 0.33 to 2.25 | 0.3 to 2.4 | 1.04 | 0.39 to 2.99 |
| Anticonvulsants vs Placebo | 1.29 | 0.55 to 2.95 | 0.51 to 3.29 | 1.26 | 0.54 to 2.99 |
| Memantine vs Placebo | 1.08 | 0.78 to 1.45 | 0.63 to 1.78 | 1.06 | 0.75 to 1.5 |
| Cholinesterase Inhibitors vs Placebo | 0.82 | 0.68 to 1 | 0.52 to 1.28 | 0.82 | 0.66 to 1 |
| Antipsychotics vs Placebo | 1.17 | 0.87 to 1.6 | 0.71 to 1.97 | 1.24 | 0.92 to 1.69 |
| Cholinesterase Inhibitor + Memantine vs Placebo | 0.85 | 0.45 to 1.67 | 0.4 to 1.9 | 0.66 | 0.22 to 1.83 |
| Anticonvulsants vs Antidepressants | 1.51 | 0.46 to 5.65 | 0.43 to 5.95 | - | - |
| Memantine vs Antidepressants | 1.29 | 0.45 to 3.48 | 0.43 to 3.81 | - | - |
| Cholinesterase Inhibitors vs Antidepressants | 0.99 | 0.36 to 2.56 | 0.34 to 2.78 | - | - |
| Antipsychotics vs Antidepressants | 1.42 | 0.5 to 3.8 | 0.46 to 4.07 | 10.65 | 0.53 to 1978.24 |
| Cholinesterase Inhibitor + Memantine vs Antidepressants | 1.01 | 0.32 to 3.33 | 0.3 to 3.55 | - | - |
| Memantine vs Anticonvulsants | 0.84 | 0.34 to 2.05 | 0.31 to 2.2 | - | - |
| Cholinesterase Inhibitors vs Anticonvulsants | 0.64 | 0.28 to 1.52 | 0.25 to 1.64 | - | - |
| Antipsychotics vs Anticonvulsants | 0.92 | 0.38 to 2.32 | 0.34 to 2.5 | - | - |
| Cholinesterase Inhibitor + Memantine vs Anticonvulsants | 0.66 | 0.24 to 1.95 | 0.22 to 2.1 | - | - |
| Cholinesterase Inhibitors vs Memantine | 0.76 | 0.54 to 1.09 | 0.45 to 1.32 | 1.27 | 0.62 to 2.72 |
| Antipsychotics vs Memantine | 1.09 | 0.73 to 1.68 | 0.61 to 2.01 | 0.4 | 0.1 to 1.32 |
| Cholinesterase Inhibitor + Memantine vs Memantine | 0.79 | 0.4 to 1.59 | 0.36 to 1.81 | 0.69 | 0.23 to 1.92 |
| Antipsychotics vs Cholinesterase Inhibitors | 1.43 | 0.99 to 2.09 | 0.82 to 2.47 | 0.96 | 0.09 to 9.9 |
| Cholinesterase Inhibitor + Memantine vs Cholinesterase Inhibitors | 1.04 | 0.55 to 2.01 | 0.49 to 2.26 | 0.96 | 0.48 to 1.95 |
| Cholinesterase Inhibitor + Memantine vs Antipsychotics | 0.72 | 0.36 to 1.5 | 0.33 to 1.68 | - | - |
| *Common within-network between-study heterogeneity variance* | *0.03* | *<0.01 to 0.15* | *-* | *0.03* | *<0.01 to 0.15* |
| *Consistency NMA model:*  *DIC statistic 374.6, residual deviance 236.8, # of data points 211, effective number of parameters 137.8* | | | | | |

Abbreviations: network meta-analysis (NMA), credible interval (CrI), predictive interval (PrI), meta-analysis (MA), odds ratio (OR), deviance information criterion (DIC)

# Table 9e. Frequentist Pairwise and Network Meta-Analysis: RCTs Reporting Mortality

| **Comparison** | **NMA OR estimate** | **95% CI** | **MA OR estimate** | **95% CI** |
| --- | --- | --- | --- | --- |
| Antidepressants vs Placebo | 0.88 | 0.36 to 2.16 | 1.01 | 0.41 to 2.5 |
| Anticonvulsants vs Placebo | 1.13 | 0.48 to 2.64 | 1.5 | 0.41 to 5.47 |
| Memantine vs Placebo | 1.05 | 0.77 to 1.44 | 1.07 | 0.78 to 1.47 |
| Cholinesterase Inhibitors vs Placebo | 0.80 | 0.65 to 0.98 | 0.8 | 0.67 to 0.96 |
| Antipsychotics vs Placebo | 1.14 | 0.84 to 1.55 | 1.21 | 0.89 to 1.66 |
| Cholinesterase Inhibitor + Memantine vs Placebo | 0.86 | 0.44 to 1.65 | 0.67 | 0.24 to 1.86 |
| Anticonvulsants vs Antidepressants | 1.28 | 0.37 to 4.42 | - | - |
| Memantine vs Antidepressants | 1.20 | 0.46 to 3.1 | - | - |
| Cholinesterase Inhibitors vs Antidepressants | 0.91 | 0.36 to 2.28 | - | - |
| Antipsychotics vs Antidepressants | 1.29 | 0.5 to 3.32 | 5.59 | 0.26 to 125 |
| Cholinesterase Inhibitor + Memantine vs Antidepressants | 0.97 | 0.32 to 2.96 | - | - |
| Memantine vs Anticonvulsants | 0.93 | 0.38 to 2.32 | - | - |
| Cholinesterase Inhibitors vs Anticonvulsants | 0.71 | 0.3 to 1.7 | - | - |
| Antipsychotics vs Anticonvulsants | 1.01 | 0.41 to 2.5 | - | - |
| Cholinesterase Inhibitor + Memantine vs Anticonvulsants | 0.76 | 0.26 to 2.22 | - | - |
| Cholinesterase Inhibitors vs Memantine | 0.76 | 0.53 to 1.08 | 1.23 | 0.64 to 2.36 |
| Antipsychotics vs Memantine | 1.08 | 0.71 to 1.65 | 0.43 | 0.13 to 1.43 |
| Cholinesterase Inhibitor + Memantine vs Memantine | 0.81 | 0.4 to 1.63 | 0.7 | 0.25 to 1.95 |
| Antipsychotics vs Cholinesterase Inhibitors | 1.43 | 0.99 to 2.05 | 1 | 0.13 to 7.59 |
| Cholinesterase Inhibitor + Memantine vs Cholinesterase Inhibitors | 1.07 | 0.57 to 2.04 | 0.98 | 0.49 to 1.97 |
| Cholinesterase Inhibitor + Memantine vs Antipsychotics | 0.75 | 0.37 to 1.55 | - | - |
| *Common within-network between-study heterogeneity variance* | *0.03* | - | *-* | *-* |
| *Design-by-treatment interaction model for inconsistency χ^2^(d.f., P-value, heterogeneity variance)* | *8.72 (8,0.37,0.03)* | - | *-* | *-* |

Abbreviations: network meta-analysis (NMA), confidence interval (CI), meta-analysis (MA), odds ratio (OR)

# Table 9f. Bayesian Network Meta-Analysis: RCTs + NRSs Reporting Mortality

| **Comparison** | **Combined RCT+NRS estimate (RR)** | **95% CrI** | **RCT estimate (RR)** | **95% CrI** | **NRS estimate (RR)** | **95% CrI** |
| --- | --- | --- | --- | --- | --- | --- |
| Antidepressants vs. Placebo | 0.86 | 0.5 to 1.48 | 0.86 | 0.49 to 1.5 | 0.87 | 0.52 to 1.43 |
| Anticonvulsants vs. Placebo | 1.28 | 0.82 to 1.99 | 1.27 | 0.81 to 2 | 1.29 | 0.85 to 1.9 |
| Memantine vs. Placebo | 1.15 | 0.84 to 1.59 | 1.12 | 0.87 to 1.43 | 1.17 | 0.87 to 1.64 |
| Cholinesterase inhibitors vs. Placebo | 0.84 | 0.65 to 1.1 | 0.84 | 0.71 to 0.99 | 0.84 | 0.68 to 1.04 |
| Antipsychotics vs. Placebo | 1.33 | 1.01 to 1.73 | 1.28 | 1 to 1.59 | 1.38 | 1.13 to 1.69 |
| Cholinesterase Inhibitor + Memantine vs. Placebo | 1.04 | 0.68 to 1.58 | 1.02 | 0.66 to 1.52 | 1.06 | 0.72 to 1.57 |
| Anxiolytic/hypnotics vs. Placebo | 0.84 | 0.41 to 1.72 | - | - | 0.84 | 0.44 to 1.61 |
| Anticonvulsants vs. Antidepressants | 1.48 | 0.76 to 2.88 | 1.48 | 0.74 to 2.96 | 1.48 | 0.81 to 2.67 |
| Memantine vs. Antidepressants | 1.33 | 0.71 to 2.48 | 1.3 | 0.71 to 2.4 | 1.35 | 0.76 to 2.43 |
| Cholinesterase Inhibitors vs. Antidepressants | 0.98 | 0.53 to 1.76 | 0.99 | 0.55 to 1.72 | 0.97 | 0.57 to 1.66 |
| Antipsychotics vs. Antidepressants | 1.54 | 0.86 to 2.7 | 1.49 | 0.82 to 2.58 | 1.59 | 0.98 to 2.58 |
| Cholinesterase Inhibitor + Memantine vs. Antidepressants | 1.21 | 0.62 to 2.27 | 1.19 | 0.6 to 2.24 | 1.23 | 0.69 to 2.17 |
| Anxiolytic/hypnotics vs. Antidepressants | 0.97 | 0.39 to 2.36 | - | - | 0.96 | 0.42 to 2.19 |
| Memantine vs. Anticonvulsants | 0.90 | 0.52 to 1.54 | 0.88 | 0.52 to 1.47 | 0.91 | 0.56 to 1.51 |
| Cholinesterase Inhibitors vs. Anticonvulsants | 0.66 | 0.39 to 1.1 | 0.66 | 0.41 to 1.07 | 0.66 | 0.42 to 1.04 |
| Antipsychotics vs. Anticonvulsants | 1.04 | 0.63 to 1.7 | 1.01 | 0.6 to 1.61 | 1.08 | 0.72 to 1.62 |
| Cholinesterase Inhibitor + Memantine vs. Anticonvulsants | 0.82 | 0.45 to 1.43 | 0.8 | 0.44 to 1.39 | 0.83 | 0.5 to 1.38 |
| Anxiolytic/hypnotics vs. Anticonvulsants | 0.66 | 0.28 to 1.52 | - | - | 0.65 | 0.31 to 1.4 |
| Cholinesterase Inhibitors vs. Memantine | 0.74 | 0.49 to 1.09 | 0.75 | 0.57 to 1.01 | 0.72 | 0.5 to 1.01 |
| Antipsychotics vs. Memantine | 1.16 | 0.76 to 1.73 | 1.14 | 0.82 to 1.57 | 1.18 | 0.82 to 1.67 |
| Cholinesterase Inhibitor + Memantine vs. Memantine | 0.91 | 0.54 to 1.49 | 0.91 | 0.57 to 1.42 | 0.91 | 0.57 to 1.44 |
| Anxiolytic/hypnotics vs. Memantine | 0.73 | 0.34 to 1.57 | - | - | 0.71 | 0.36 to 1.43 |
| Antipsychotics vs. Cholinesterase Inhibitors | 1.58 | 1.07 to 2.28 | 1.52 | 1.13 to 1.99 | 1.63 | 1.24 to 2.2 |
| Cholinesterase Inhibitor + Memantine vs. Cholinesterase Inhibitors | 1.23 | 0.76 to 1.98 | 1.21 | 0.78 to 1.82 | 1.26 | 0.83 to 1.94 |
| Anxiolytic/hypnotics vs. Cholinesterase Inhibitors | 0.99 | 0.47 to 2.1 | - | - | 0.99 | 0.52 to 1.92 |
| Cholinesterase Inhibitor + Memantine vs. Antipsychotics | 0.78 | 0.48 to 1.27 | 0.79 | 0.51 to 1.25 | 0.77 | 0.52 to 1.15 |
| Anxiolytic/hypnotics vs. Antipsychotics | 0.63 | 0.3 to 1.37 | - | - | 0.61 | 0.31 to 1.2 |
| Anxiolytic/hypnotics vs. Cholinesterase Inhibitor + Memantine | 0.80 | 0.35 to 1.86 | - | - | 0.79 | 0.37 to 1.69 |
| *Between-study type heterogeneity variance* | *0.01 (95% CrI <0.01 to 0.14)* | | | | | |
| *Between-RCT heterogeneity variance* | *0.03 (95% CrI <0.01 to 0.12)* | | | | | |
| *Between-NRS heterogeneity variance* | *0.15 (95% CrI 0.08 to 0.29)* | | | | | |

Abbreviations: credible interval (CrI), network meta-analysis (NMA), nonrandomized study (NRS), randomized trial (RCT), relative risk (RR)

# Table 9g. Bayesian Pairwise and Network Meta-Analysis: RCTs Reporting Cerebrovascular Event

| **Comparison** | **NMA OR estimate** | **95% CrI** | **95% PrI** | **MA OR estimate** | **95% CrI** |
| --- | --- | --- | --- | --- | --- |
| Antidepressants vs Placebo | 0.19 | <0.01 to 5.85 | <0.01 to 6.04 | 0.18 | <0.01 to 5.19 |
| Anticonvulsants vs Placebo | 1.04 | 0.1 to 11.39 | 0.1 to 11.93 | 1.01 | 0.1 to 10.36 |
| Memantine vs Placebo | 0.76 | 0.45 to 1.29 | 0.38 to 1.54 | 0.83 | 0.48 to 1.47 |
| Cholinesterase Inhibitors vs Placebo | 1.02 | 0.72 to 1.48 | 0.57 to 1.88 | 1 | 0.7 to 1.46 |
| Antipsychotics vs Placebo | 2.23 | 1.36 to 3.79 | 1.12 to 4.49 | 2.13 | 1.28 to 3.7 |
| Cholinesterase Inhibitor + Memantine vs Placebo | 1.11 | 0.29 to 3.8 | 0.27 to 4.15 | 0.99 | 0.17 to 5.98 |
| Dextromethorphan-Quinidine vs Placebo | 1.35 | 0.03 to 55.23 | 0.03 to 56.57 | 1.41 | 0.04 to 60.64 |
| Anticonvulsants vs Antidepressants | 6.19 | 0.08 to 4296 | 0.08 to 4360 | - | - |
| Memantine vs Antidepressants | 4.09 | 0.13 to 2251 | 0.12 to 2279 | - | - |
| Cholinesterase Inhibitors vs Antidepressants | 5.49 | 0.17 to 3123 | 0.16 to 3176 | - | - |
| Antipsychotics vs Antidepressants | 12.08 | 0.37 to 6881 | 0.36 to 7014 | - | - |
| Cholinesterase Inhibitor + Memantine vs Antidepressants | 6.04 | 0.15 to 3490 | 0.14 to 3519 | - | - |
| Dextromethorphan-Quinidine vs Antidepressants | 8.02 | 0.04 to 6267 | 0.04 to 6293 | - | - |
| Memantine vs Anticonvulsants | 0.73 | 0.06 to 7.85 | 0.06 to 8.26 | - | - |
| Cholinesterase Inhibitors vs Anticonvulsants | 0.99 | 0.09 to 10.26 | 0.09 to 10.76 | - | - |
| Antipsychotics vs Anticonvulsants | 2.14 | 0.19 to 23.98 | 0.18 to 25.11 | - | - |
| Cholinesterase Inhibitor + Memantine vs Anticonvulsants | 1.05 | 0.07 to 14.58 | 0.06 to 15.24 | - | - |
| Dextromethorphan-Quinidine vs Anticonvulsants | 1.27 | 0.02 to 99.02 | 0.02 to 101.8 | - | - |
| Cholinesterase Inhibitors vs Memantine | 1.34 | 0.72 to 2.54 | 0.62 to 2.96 | 7.3 | 0.97 to 177.9 |
| Antipsychotics vs Memantine | 2.94 | 1.44 to 6.07 | 1.24 to 6.9 | 13.89 | 0.92 to 7007.71 |
| Cholinesterase Inhibitor + Memantine vs Memantine | 1.46 | 0.36 to 5.29 | 0.33 to 5.74 | 4.14 | 0.42 to 141.6 |
| Dextromethorphan-Quinidine vs Memantine | 1.78 | 0.04 to 73.61 | 0.04 to 76.51 | - | - |
| Antipsychotics vs Cholinesterase Inhibitors | 2.18 | 1.17 to 4.11 | 0.99 to 4.74 | - | - |
| Cholinesterase Inhibitor + Memantine vs Cholinesterase Inhibitors | 1.09 | 0.28 to 3.72 | 0.26 to 4.06 | 0.78 | 0.19 to 3.16 |
| Dextromethorphan-Quinidine vs Cholinesterase Inhibitors | 1.31 | 0.03 to 55.75 | 0.03 to 57.18 | - | - |
| Cholinesterase Inhibitor + Memantine vs Antipsychotics | 0.5 | 0.12 to 1.88 | 0.11 to 2.03 | - | - |
| Dextromethorphan-Quinidine vs Antipsychotics | 0.61 | 0.01 to 24.57 | 0.01 to 25.44 | - | - |
| Dextromethorphan-Quinidine vs Cholinesterase Inhibitor + Memantine | 1.22 | 0.02 to 60.44 | 0.02 to 61.92 | - | - |
| *Common within-network between-study heterogeneity variance* | *0.03* | *<0.01 to 0.26* | *-* | *0.03* | *<0.01 to 0.27* |
| *Consistency NMA model:*  *DIC statistic 123.5, residual deviance 91.8, # of data points 96, effective number of parameters 31.7* | | | | | |

Abbreviations: network meta-analysis (NMA), credible interval (CrI), predictive interval (PrI), meta-analysis (MA), odds ratio (OR), deviance information criterion (DIC)

# Table 9h. Frequentist Pairwise and Network Meta-Analysis: RCTs Reporting Cerebrovascular Event

| **Comparison** | **NMA OR estimate** | **95% CI** | **MA OR estimate** | **95% CI** |
| --- | --- | --- | --- | --- |
| Antidepressants vs Placebo | 0.32 | 0.01 to 8.23 | 0.32 | 0.01 to 8.23 |
| Anticonvulsants vs Placebo | 1.03 | 0.1 to 10.03 | 1.03 | 0.11 to 10.03 |
| Memantine vs Placebo | 0.74 | 0.43 to 1.28 | 0.79 | 0.46 to 1.38 |
| Cholinesterase Inhibitors vs Placebo | 1.01 | 0.72 to 1.39 | 0.99 | 0.71 to 1.38 |
| Antipsychotics vs Placebo | 1.94 | 1.15 to 3.27 | 1.88 | 1.1 to 3.19 |
| Cholinesterase Inhibitor + Memantine vs Placebo | 0.99 | 0.28 to 3.46 | 1 | 0.2 to 5.13 |
| Dextromethorphan-Quinidine vs Placebo | 1.37 | 0.08 to 22.18 | 1.37 | 0.09 to 22.18 |
| Anticonvulsants vs Antidepressants | 3.16 | 0.06 to 165.22 | - | - |
| Memantine vs Antidepressants | 2.29 | 0.09 to 60.71 | - | - |
| Cholinesterase Inhibitors vs Antidepressants | 3.10 | 0.12 to 79.86 | - | - |
| Antipsychotics vs Antidepressants | 5.98 | 0.23 to 158.09 | - | - |
| Cholinesterase Inhibitor + Memantine vs Antidepressants | 3.05 | 0.1 to 97.82 | - | - |
| Dextromethorphan-Quinidine vs Antidepressants | 4.22 | 0.06 to 300.99 | - | - |
| Memantine vs Anticonvulsants | 0.72 | 0.07 to 7.55 | - | - |
| Cholinesterase Inhibitors vs Anticonvulsants | 0.98 | 0.1 to 9.82 | - | - |
| Antipsychotics vs Anticonvulsants | 1.89 | 0.18 to 19.64 | - | - |
| Cholinesterase Inhibitor + Memantine vs Anticonvulsants | 0.97 | 0.07 to 13.03 | - | - |
| Dextromethorphan-Quinidine vs Anticonvulsants | 1.34 | 0.04 to 48.86 | - | - |
| Cholinesterase Inhibitors vs Memantine | 1.35 | 0.72 to 2.53 | 5.52 | 0.63 to 48.4 |
| Antipsychotics vs Memantine | 2.61 | 1.25 to 5.48 | 7.3 | 0.37 to 142.9 |
| Cholinesterase Inhibitor + Memantine vs Memantine | 1.33 | 0.35 to 5.09 | 3.21 | 0.33 to 31.63 |
| Dextromethorphan-Quinidine vs Memantine | 1.84 | 0.11 to 31.46 | - | - |
| Antipsychotics vs Cholinesterase Inhibitors | 1.93 | 1.04 to 3.58 | - | - |
| Cholinesterase Inhibitor + Memantine vs Cholinesterase Inhibitors | 0.99 | 0.28 to 3.41 | 0.77 | 0.2 to 2.92 |
| Dextromethorphan-Quinidine vs Cholinesterase Inhibitors | 1.36 | 0.08 to 22.49 | - | - |
| Cholinesterase Inhibitor + Memantine vs Antipsychotics | 0.51 | 0.13 to 1.98 | - | - |
| Dextromethorphan-Quinidine vs Antipsychotics | 0.71 | 0.04 to 12.01 | - | - |
| Dextromethorphan-Quinidine vs Cholinesterase Inhibitor + Memantine | 1.38 | 0.07 to 29.27 | - | - |
| *Common within-network between-study heterogeneity variance* | <0.01 | - | *-* | *-* |
| *Design-by-treatment interaction model for inconsistency χ^2^(d.f., P-value, heterogeneity variance)* | 2.96 (4,0.56,<0.01) | - | *-* | *-* |

Abbreviations: network meta-analysis (NMA), confidence interval (CI), meta-analysis (MA), odds ratio (OR)

# Table 9i. Bayesian Network Meta-Analysis: RCTs + NRSs Reporting Cerebrovascular Event

| **Comparison** | **Combined RCT+NRS estimate (RR)** | **95% CrI** | **RCT estimate (RR)** | **95% CrI** | **NRS estimate (RR)** | **95% CrI** |
| --- | --- | --- | --- | --- | --- | --- |
| Antipsychotics vs. Placebo | 1.76 | 0.57 to 4.79 | 2.02 | 1.25 to 3.39 | 1.51 | 0.63 to 3.28 |
| Cholinesterase Inhibitor vs. Placebo | 0.93 | 0.3 to 2.5 | 0.99 | 0.72 to 1.42 | 0.86 | 0.33 to 1.89 |
| Memantine vs. Placebo | 0.76 | 0.18 to 3.11 | 0.76 | 0.45 to 1.28 | - | - |
| Anticonvulsants vs. Placebo | 1.03 | 0.1 to 11.56 | 1.02 | 0.15 to 8.05 | - | - |
| Antidepressants vs. Placebo | 0.27 | <0.01 to 8.34 | 0.28 | <0.01 to 6.8 | - | - |
| Cholinesterase Inhibitor + Memantine vs. Placebo | 1.04 | 0.16 to 5.73 | 1.05 | 0.27 to 3.29 | - | - |
| Dextromethorphan-Quinidine vs. Placebo | 1.11 | 0.03 to 40.95 | 1.11 | 0.04 to 30.25 | - | - |
| Cholinesterase Inhibitor vs. Antipsychotics | 0.53 | 0.12 to 2.35 | 0.5 | 0.26 to 0.89 | 0.56 | 0.18 to 1.81 |
| Memantine vs. Antipsychotics | 0.43 | 0.08 to 2.69 | 0.37 | 0.18 to 0.77 | - | - |
| Anticonvulsants vs. Antipsychotics | 0.59 | 0.05 to 8.33 | 0.51 | 0.07 to 4.17 | - | - |
| Antidepressants vs. Antipsychotics | 0.16 | <0.01 to 5.45 | 0.14 | <0.01 to 3.43 | - | - |
| Cholinesterase Inhibitors + Memantine vs. Antipsychotics | 0.58 | 0.07 to 4.69 | 0.51 | 0.12 to 1.83 | - | - |
| Dextromethorphan-Quinidine vs. Antipsychotics | 0.63 | 0.01 to 27.7 | 0.54 | 0.02 to 15.5 | - | - |
| Memantine vs. Cholinesterase Inhibitor | 0.82 | 0.15 to 5.13 | 0.76 | 0.41 to 1.42 | - | - |
| Anticonvulsants vs. Cholinesterase Inhibitor | 1.12 | 0.09 to 16.26 | 1.02 | 0.15 to 8.05 | - | - |
| Antidepressants vs. Cholinesterase Inhibitor | 0.30 | <0.01 to 10.44 | 0.28 | <0.01 to 6.8 | - | - |
| Cholinesterase Inhibitor + Memantine vs. Cholinesterase Inhibitor | 1.11 | 0.14 to 8.71 | 1.04 | 0.27 to 3.32 | - | - |
| Dextromethorphan-Quinidine vs. Cholinesterase Inhibitor | 1.22 | 0.03 to 53.83 | 1.12 | 0.04 to 32.15 | - | - |
| Anticonvulsants vs. Memantine | 1.36 | 0.09 to 22.05 | 1.35 | 0.19 to 11.55 | - | - |
| Antidepressants vs. Memantine | 0.36 | <0.01 to 13.9 | 0.37 | <0.01 to 9.03 | - | - |
| Cholinesterase Inhibitor + Memantine vs. Memantine | 1.36 | 0.14 to 12.25 | 1.37 | 0.33 to 4.65 | - | - |
| Dextromethorphan-Quinidine vs. Memantine | 1.48 | 0.03 to 71.93 | 1.45 | 0.05 to 44.25 | - | - |
| Antidepressants vs. Anticonvulsants | 0.26 | <0.01 to 17.24 | 0.25 | <0.01 to 12.18 | - | - |
| Cholinesterase Inhibitor + Memantine vs. Anticonvulsants | 0.99 | 0.04 to 18.94 | 1.0 | 0.08 to 10.09 | - | - |
| Dextromethorphan-Quinidine vs. Anticonvulsants | 1.03 | 0.01 to 85.89 | 1.02 | 0.02 to 54.64 | - | - |
| Cholinesterase Inhibitor + Memantine vs. Antidepressants | 3.54 | 0.09 to 328 | 3.41 | 0.15 to 257.1 | - | - |
| Dextromethorphan-Quindine vs. Antidepressants | 3.98 | 0.03 to 782.2 | 3.94 | 0.05 to 600.4 | - | - |
| Dextromethorphan-Quindine vs. Cholinesterase inhibitor + Memantine | 1.13 | 0.02 to 69.19 | 1.13 | 0.03 to 44.5 | - | - |
| *Between-study type heterogeneity variance* | *0.14 (95% CrI <0.01 to 2.35)* | | | | | |
| *Between-RCT heterogeneity variance* | *0.03 (95% CrI <0.01 to 0.25)* | | | | | |
| *Between-NRS heterogeneity variance* | *0.61 (95% CrI 0.15 to 2.72)* | | | | | |

Abbreviations: credible interval (CrI), network meta-analysis (NMA), nonrandomized study (NRS), randomized trial (RCT), relative risk (RR)

# Table 9j. Bayesian Pairwise and Network Meta-Analysis: RCTs Reporting Falls

| **Comparison** | **NMA OR estimate** | **95% CrI** | **95% PrI** | **MA OR estimate** | **95% CrI** |
| --- | --- | --- | --- | --- | --- |
| Antidepressants vs Placebo | 1.06 | 0.54 to 2.09 | 0.52 to 2.16 | 1.27 | 0.59 to 2.77 |
| Anticonvulsants vs Placebo | 1.31 | 0.89 to 1.95 | 0.85 to 2.04 | 1.33 | 0.93 to 1.89 |
| Memantine vs Placebo | 0.95 | 0.77 to 1.17 | 0.71 to 1.28 | 0.93 | 0.75 to 1.15 |
| Cholinesterase Inhibitors vs Placebo | 0.93 | 0.77 to 1.14 | 0.7 to 1.25 | 0.96 | 0.78 to 1.17 |
| Antipsychotics vs Placebo | 0.99 | 0.79 to 1.23 | 0.73 to 1.34 | 0.98 | 0.79 to 1.23 |
| Cholinesterase Inhibitor + Memantine vs Placebo | 0.97 | 0.63 to 1.48 | 0.6 to 1.55 | 0.2 | 0.04 to 0.71 |
| Dextromethorphan-Quinidine vs Placebo | 4.24 | 1.47 to 13.79 | 1.44 to 14.03 | 4.2 | 1.49 to 13.29 |
| Anticonvulsants vs Antidepressants | 1.24 | 0.56 to 2.73 | 0.54 to 2.8 | - | - |
| Memantine vs Antidepressants | 0.89 | 0.44 to 1.82 | 0.43 to 1.88 | - | - |
| Cholinesterase Inhibitors vs Antidepressants | 0.88 | 0.43 to 1.79 | 0.42 to 1.85 | - | - |
| Antipsychotics vs Antidepressants | 0.93 | 0.46 to 1.88 | 0.44 to 1.94 | 0.37 | 0.02 to 2.63 |
| Cholinesterase Inhibitor + Memantine vs Antidepressants | 0.91 | 0.4 to 2.04 | 0.39 to 1.36 | - | - |
| Dextromethorphan-Quinidine vs Antidepressants | 4.02 | 1.1 to 15.56 | 1.09 to 15.83 | - | - |
| Memantine vs Anticonvulsants | 0.72 | 0.46 to 1.12 | 0.44 to 1.17 | - | - |
| Cholinesterase Inhibitors vs Anticonvulsants | 0.71 | 0.46 to 1.1 | 0.44 to 1.15 | - | - |
| Antipsychotics vs Anticonvulsants | 0.75 | 0.48 to 1.17 | 0.46 to 1.23 | - | - |
| Cholinesterase Inhibitor + Memantine vs Anticonvulsants | 0.74 | 0.41 to 1.31 | 0.39 to 1.36 | - | - |
| Dextromethorphan-Quinidine vs Anticonvulsants | 3.24 | 1.03 to 11.03 | 1.02 to 11.22 | - | - |
| Cholinesterase Inhibitors vs Memantine | 0.99 | 0.74 to 1.31 | 0.69 to 1.4 | 1.21 | 0.43 to 3.45 |
| Antipsychotics vs Memantine | 1.04 | 0.77 to 1.41 | 0.72 to 1.5 | - | - |
| Cholinesterase Inhibitor + Memantine vs Memantine | 1.02 | 0.64 to 1.62 | 0.61 to 1.69 | 0.35 | 0.07 to 1.28 |
| Dextromethorphan-Quinidine vs Memantine | 4.48 | 1.51 to 14.89 | 1.48 to 15.13 | - | - |
| Antipsychotics vs Cholinesterase Inhibitors | 1.06 | 0.79 to 1.43 | 0.74 to 1.52 | - | - |
| Cholinesterase Inhibitor + Memantine vs Cholinesterase Inhibitors | 1.04 | 0.69 to 1.54 | 0.66 to 1.61 | 1.13 | 0.74 to 1.75 |
| Dextromethorphan-Quinidine vs Cholinesterase Inhibitors | 4.53 | 1.55 to 15.06 | 1.51 to 15.39 | - | - |
| Cholinesterase Inhibitor + Memantine vs Antipsychotics | 0.98 | 0.6 to 1.58 | 0.58 to 1.65 | - | - |
| Dextromethorphan-Quinidine vs Antipsychotics | 4.3 | 1.46 to 14.14 | 1.43 to 14.41 | - | - |
| Dextromethorphan-Quinidine vs Cholinesterase Inhibitor + Memantine | 4.38 | 1.4 to 15.41 | 1.38 to 15.69 | - | - |
| *Common within-network between-study heterogeneity variance* | *0.01* | *<0.01 to 0.04* | *-* | *0.01* | *<0.01 to 0.04* |
| *Consistency NMA model:*  *DIC statistic 58.5, residual deviance 101.9, # of data points118, effective number of parameters -43.4* | | | | | |

Abbreviations: network meta-analysis (NMA), credible interval (CrI), predictive interval (PrI), meta-analysis (MA), odds ratio (OR), deviance information criterion (DIC)

# Table 9k. Frequentist Pairwise and Network Meta-Analysis: RCTs Reporting Falls

| **Comparison** | **NMA OR estimate** | **95% CI** | **MA OR estimate** | **95% CI** |
| --- | --- | --- | --- | --- |
| Antidepressants vs Placebo | 1.13 | 0.55 to 2.32 | 1.28 | 0.6 to 2.76 |
| Anticonvulsants vs Placebo | 1.33 | 0.94 to 1.87 | 1.33 | 0.94 to 1.87 |
| Memantine vs Placebo | 0.96 | 0.79 to 1.18 | 0.92 | 0.75 to 1.13 |
| Cholinesterase Inhibitors vs Placebo | 0.94 | 0.78 to 1.13 | 0.95 | 0.79 to 1.15 |
| Antipsychotics vs Placebo | 0.98 | 0.79 to 1.2 | 0.97 | 0.79 to 1.19 |
| Cholinesterase Inhibitor + Memantine vs Placebo | 1.06 | 0.68 to 1.66 | 0.22 | 0.06 to 0.81 |
| Dextromethorphan-Quinidine vs Placebo | 3.97 | 1.36 to 11.55 | 3.97 | 1.36 to 11.55 |
| Anticonvulsants vs Antidepressants | 1.17 | 0.53 to 2.6 | - | - |
| Memantine vs Antidepressants | 0.85 | 0.4 to 1.8 | - | - |
| Cholinesterase Inhibitors vs Antidepressants | 0.83 | 0.39 to 1.74 | - | - |
| Antipsychotics vs Antidepressants | 0.86 | 0.41 to 1.81 | 2.22 | 0.28 to 17.86 |
| Cholinesterase Inhibitor + Memantine vs Antidepressants | 0.94 | 0.4 to 2.18 | - | - |
| Dextromethorphan-Quinidine vs Antidepressants | 3.51 | 0.97 to 12.72 | - | - |
| Memantine vs Anticonvulsants | 0.73 | 0.49 to 1.08 | - | - |
| Cholinesterase Inhibitors vs Anticonvulsants | 0.71 | 0.48 to 1.04 | - | - |
| Antipsychotics vs Anticonvulsants | 0.74 | 0.49 to 1.1 | - | - |
| Cholinesterase Inhibitor + Memantine vs Anticonvulsants | 0.80 | 0.45 to 1.4 | - | - |
| Dextromethorphan-Quinidine vs Anticonvulsants | 2.99 | 0.97 to 9.18 | - | - |
| Cholinesterase Inhibitors vs Memantine | 0.97 | 0.74 to 1.28 | 1.2 | 0.44 to 3.29 |
| Antipsychotics vs Memantine | 1.01 | 0.76 to 1.35 | - | - |
| Cholinesterase Inhibitor + Memantine vs Memantine | 1.10 | 0.67 to 1.79 | 0.36 | 0.09 to 1.43 |
| Dextromethorphan-Quinidine vs Memantine | 4.11 | 1.38 to 12.21 | - | - |
| Antipsychotics vs Cholinesterase Inhibitors | 1.04 | 0.79 to 1.38 | - | - |
| Cholinesterase Inhibitor + Memantine vs Cholinesterase Inhibitors | 1.13 | 0.75 to 1.7 | 1.11 | 0.67 to 1.84 |
| Dextromethorphan-Quinidine vs Cholinesterase Inhibitors | 4.23 | 1.43 to 12.52 | - | - |
| Cholinesterase Inhibitor + Memantine vs Antipsychotics | 1.08 | 0.66 to 1.78 | - | - |
| Dextromethorphan-Quinidine vs Antipsychotics | 4.06 | 1.37 to 12.07 | - | - |
| Dextromethorphan-Quinidine vs Cholinesterase Inhibitor + Memantine | 3.75 | 1.18 to 11.95 | - | - |
| *Common within-network between-study heterogeneity variance* | <0.01 | - | *-* | *-* |
| *Design-by-treatment interaction model for inconsistency χ^2^(d.f., P-value, heterogeneity variance)* | 7.34(4,0.12,<0.01) | - | *-* | *-* |

Abbreviations: network meta-analysis (NMA), confidence interval (CI), meta-analysis (MA), odds ratio (OR)

# Table 9l. Bayesian Network Meta-Analysis: RCTs + NRSs Reporting Falls

| **Comparison** | **Combined RCT+NRS estimate (RR)** | **95% CrI** | **RCT estimate (RR)** | **95% CrI** | **NRS estimate (RR)** | **95% CrI** |
| --- | --- | --- | --- | --- | --- | --- |
| Antidepressants vs. Placebo | 1.32 | 0.49 to 3.98 | 1.16 | 0.67 to 1.97 | 1.59 | 0.79 to 2.84 |
| Antipsychotics vs. Placebo | 1.21 | 0.5 to 3.74 | 1.05 | 0.91 to 1.23 | 1.54 | 0.82 to 2.88 |
| Memantine vs. Placebo | 0.95 | 0.24 to 3.82 | 0.95 | 0.79 to 1.15 | - | - |
| Cholinesterase Inhibitors vs. Placebo | 0.94 | 0.24 to 3.73 | 0.94 | 0.79 to 1.14 | - | - |
| Anticonvulsants vs. Placebo | 1.09 | 0.26 to 4.6 | 1.09 | 0.64 to 1.89 | - | - |
| Cholinesterase Inhibitor + Memantine vs. Placebo | 0.97 | 0.24 to 3.94 | 0.96 | 0.66 to 1.43 | - | - |
| Dextromethorphan-Quinidine vs. Placebo | 3.66 | 0.72 to 18.91 | 3.65 | 1.4 to 10.42 | - | - |
| Antipsychotics vs. Antidepressants | 0.93 | 0.24 to 3.95 | 0.91 | 0.53 to 1.58 | 0.97 | 0.62 to 1.56 |
| Memantine vs. Antidepressants | 0.73 | 0.12 to 3.83 | 0.82 | 0.47 to 1.47 | - | - |
| Cholinesterase Inhibitors vs. Antidepressants | 0.72 | 0.12 to 3.73 | 0.82 | 0.47 to 1.45 | - | - |
| Anticonvulsants vs. Antidepressants | 0.83 | 0.13 to 4.58 | 0.94 | 0.44 to 2.07 | - | - |
| Cholinesterase Inhibitor + Memantine vs. Antidepressants | 0.74 | 0.12 to 3.89 | 0.84 | 0.43 to 1.63 | - | - |
| Dextromethorphan-Quinidine vs. Antidepressants | 2.78 | 0.38 to 18.03 | 3.2 | 1.07 to 9.93 | - | - |
| Memantine vs. Antipsychotics | 0.79 | 0.13 to 3.85 | 0.9 | 0.71 to 1.15 | - | - |
| Cholinesterase Inhibitors vs. Antipsychotics | 0.79 | 0.12 to 3.79 | 0.89 | 0.71 to 1.14 | - | - |
| Anticonvulsants vs. Antipsychotics | 0.89 | 0.14 to 4.61 | 1.04 | 0.59 to 1.84 | - | - |
| Cholinesterase Inhibitor + Memantine vs. Antipsychotics | 0.80 | 0.12 to 3.97 | 0.91 | 0.61 to 1.39 | - | - |
| Dextromethorphan-Quinidine vs. Antipsychotics | 2.97 | 0.4 to 18.52 | 3.44 | 1.29 to 9.96 | - | - |
| Cholinesterase Inhibitors vs. Memantine | 0.99 | 0.14 to 6.96 | 0.99 | 0.77 to 1.27 | - | - |
| Anticonvulsants vs. Memantine | 1.14 | 0.16 to 8.44 | 1.14 | 0.65 to 2.04 | - | - |
| Cholinesterase Inhibitor + Memantine vs. Memantine | 1.02 | 0.14 to 7.24 | 1.01 | 0.67 to 1.56 | - | - |
| Dextromethorphan-Quindine vs. Memantine | 3.86 | 0.46 to 32.48 | 3.83 | 1.43 to 11.42 | - | - |
| Anticonvulsants vs. Cholinesterase Inhibitors | 1.15 | 0.16 to 8.57 | 1.16 | 0.65 to 2.08 | - | - |
| Cholinesterase Inhibitor + Memantine vs. Cholinesterase Inhibitors | 1.03 | 0.14 to 7.24 | 1.02 | 0.73 to 1.48 | - | - |
| Dextromethorphan-Quinidine vs. Cholinesterase Inhibitors | 3.88 | 0.47 to 32.73 | 3.84 | 1.44 to 11.3 | - | - |
| Cholinesterase Inhibitor + Memantine vs. Anticonvulsants | 0.89 | 0.12 to 6.59 | 0.89 | 0.45 to 1.72 | - | - |
| Dextromethorphan-Quinidine vs. Anticonvulsants | 3.38 | 0.38 to 29.48 | 3.36 | 1.08 to 10.97 | - | - |
| Dextromethorphan-Quinidine vs. Cholinesterase Inhibitor + Memantine | 3.81 | 0.44 to 33.04 | 3.81 | 1.33 to 12.01 | - | - |
| *Between-study type heterogeneity variance* | *0.16 (95% CrI <0.01 to 2.36)* | | | | | |
| *Between-RCT heterogeneity variance* | *0.01 (95% CrI <0.01 to 0.04)* | | | | | |
| *Between-NRS heterogeneity variance* | *0.06 (95% CrI <0.01 to 1.09)* | | | | | |

Abbreviations: credible interval (CrI), network meta-analysis (NMA), nonrandomized study (NRS), randomized trial (RCT), relative risk (RR)

# Figure 2a. Comparison-Adjusted Funnel Plot: RCTs Reporting Fracture


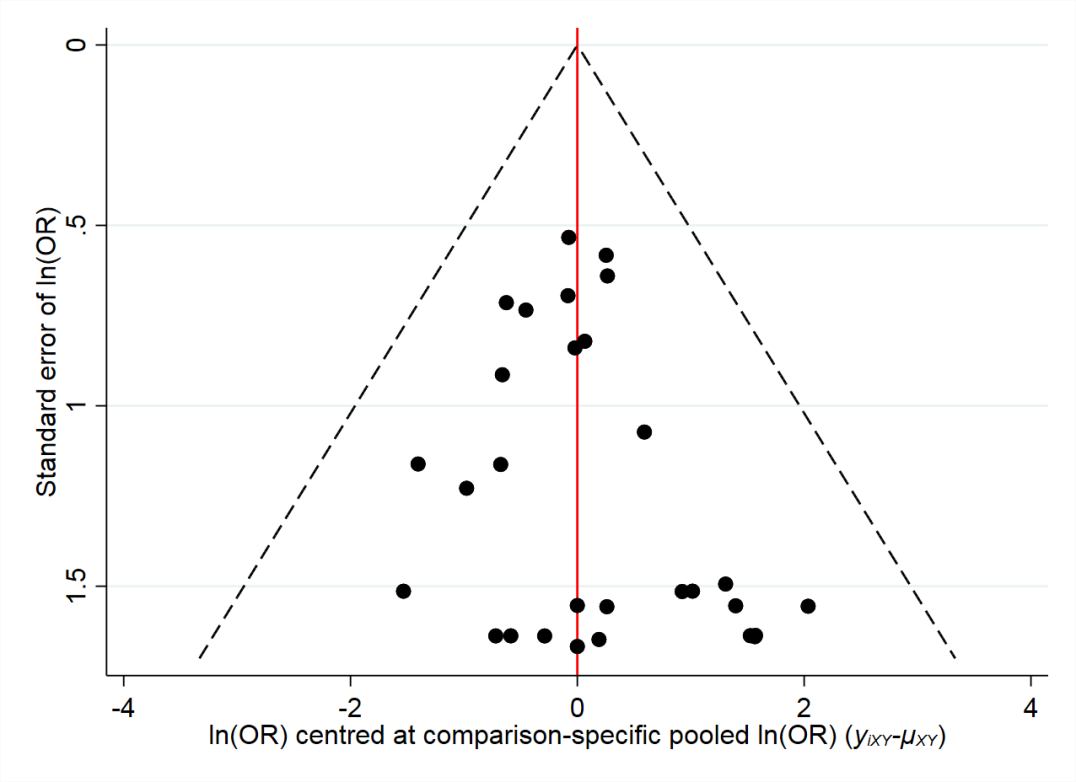


On visual inspection, there is no evidence of publication bias or small-study effects.

# Figure 2b. Comparison-Adjusted Funnel Plot: RCTs + NRSs Reporting Fracture


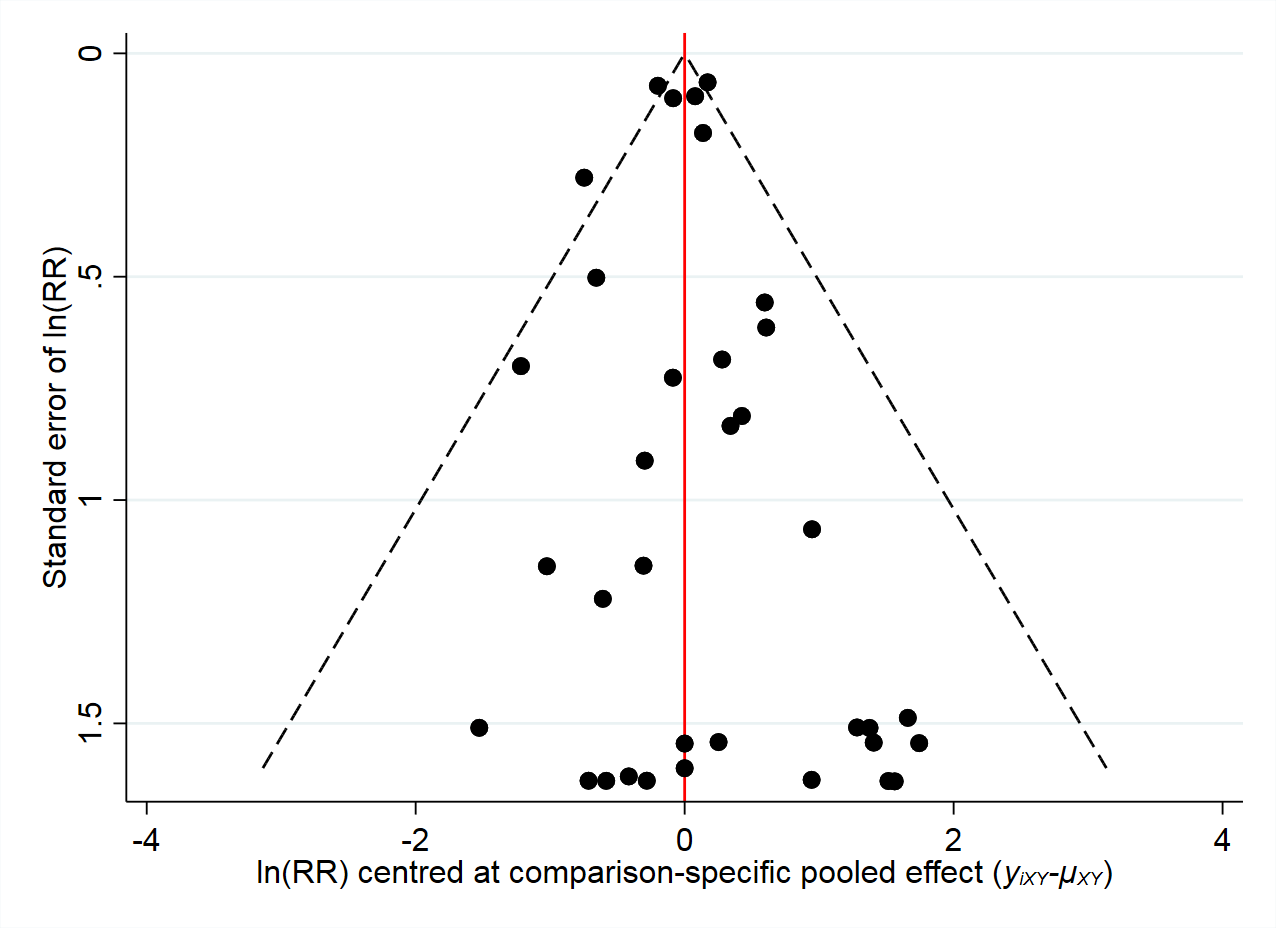


On visual inspection, there is no evidence of publication bias or small-study effects.

# Figure 2c. Comparison-Adjusted Funnel Plot: RCTs Reporting Mortality


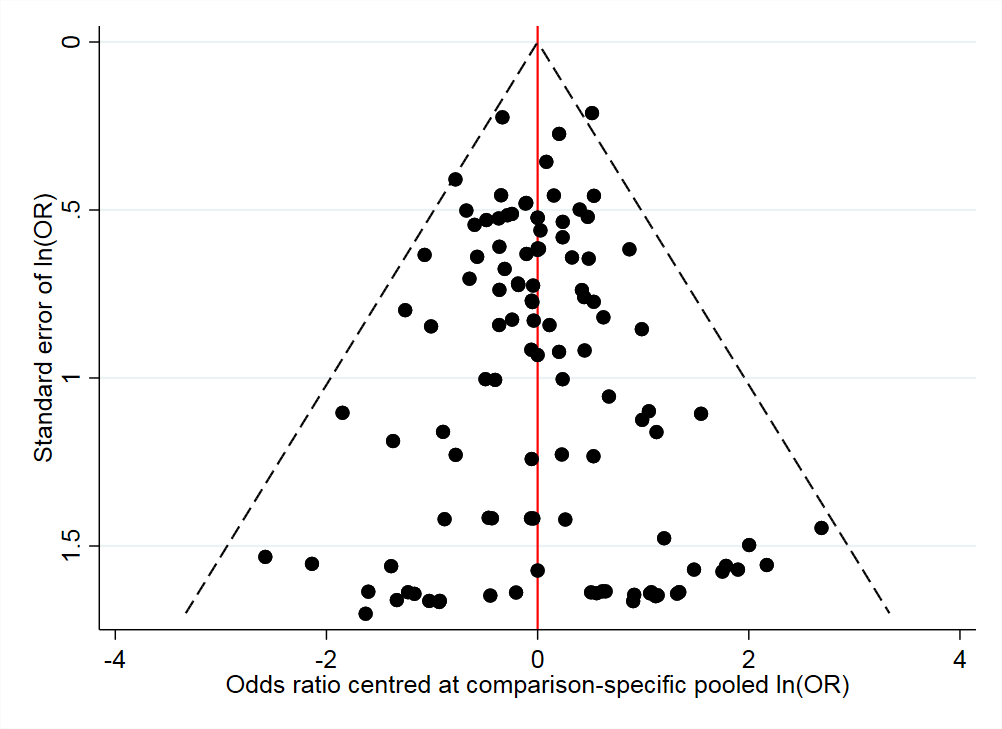


On visual inspection, there is no evidence of publication bias or small-study effects.

# Figure 2d. Comparison-Adjusted Funnel Plot: RCTs + NRSs Reporting Mortality


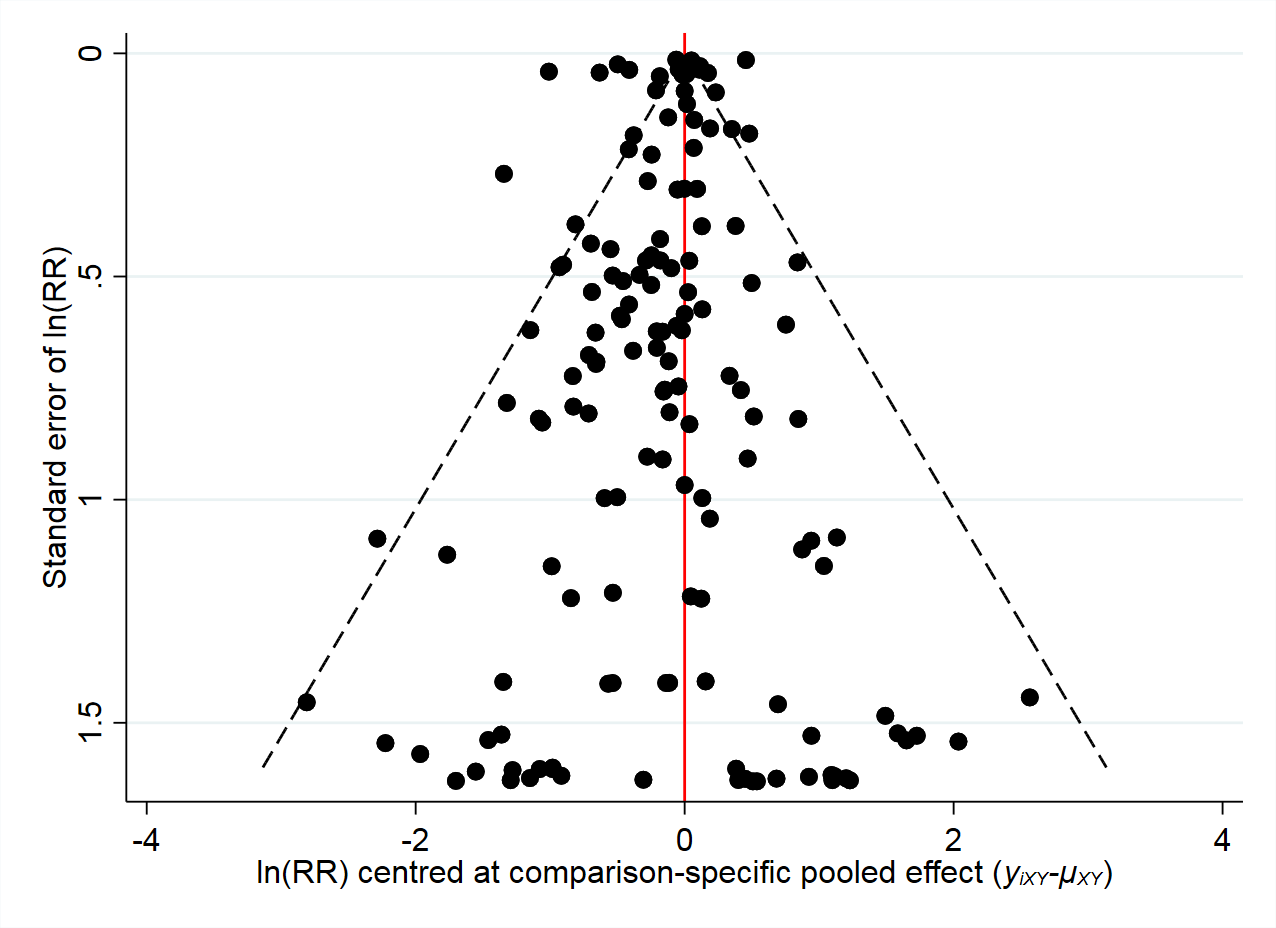


On visual inspection, there is no evidence of publication bias or small-study effects.

# Figure 2e. Comparison-Adjusted Funnel Plot: RCTs Reporting Cerebrovascular Event


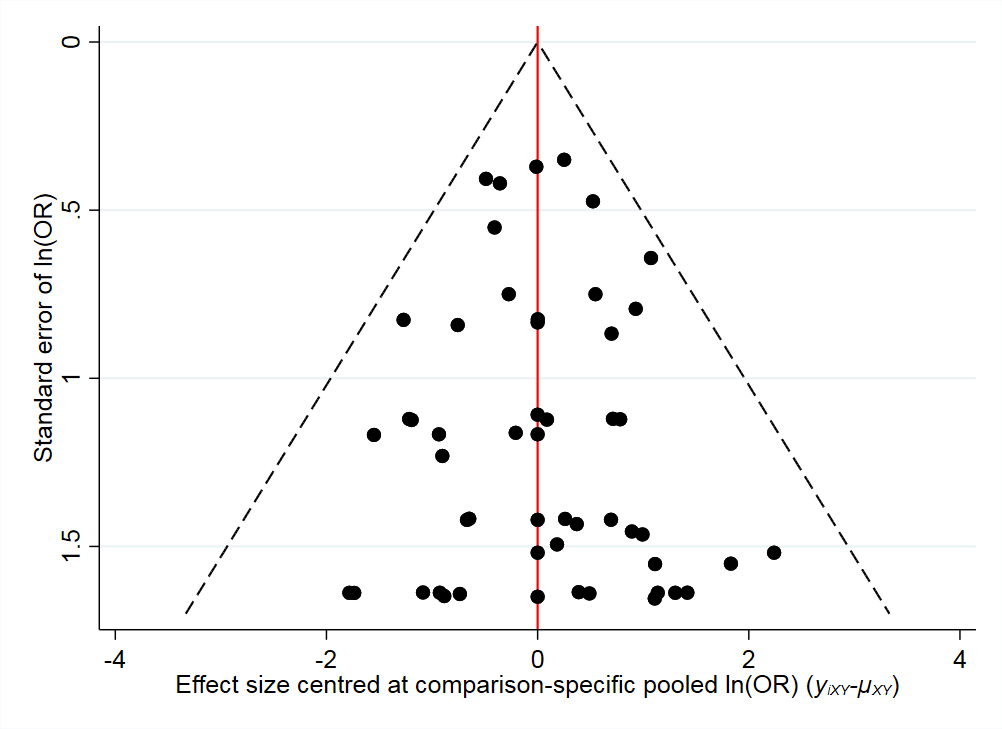


On visual inspection, there is no evidence of publication bias or small-study effects.

# Figure 2f. Comparison-Adjusted Funnel Plot: RCTs + NRSs Reporting Cerebrovascular Event


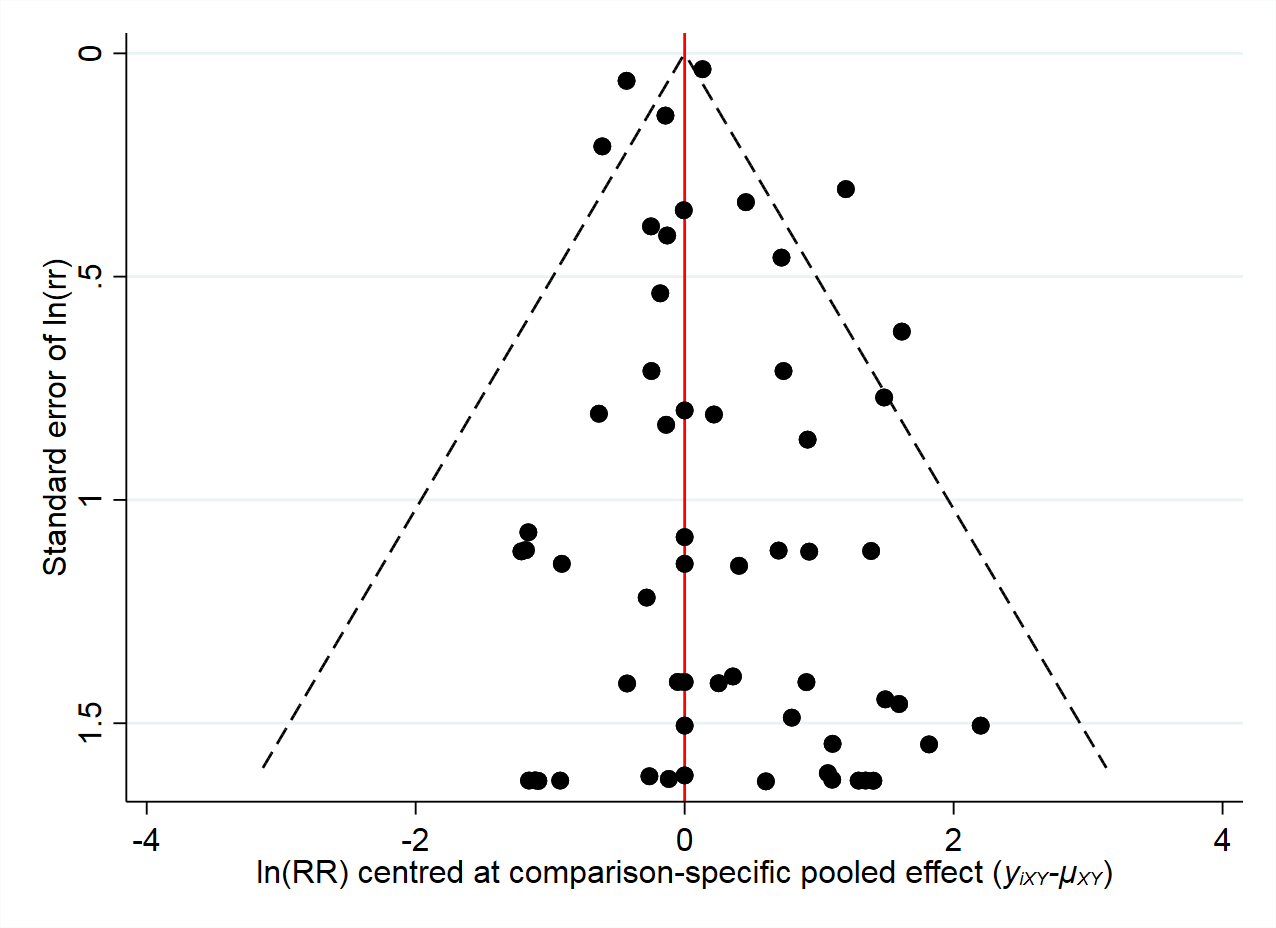


On visual inspection, there is no evidence of publication bias or small-study effects.

# Figure 2g. Comparison-Adjusted Funnel Plot: RCTs Reporting Falls


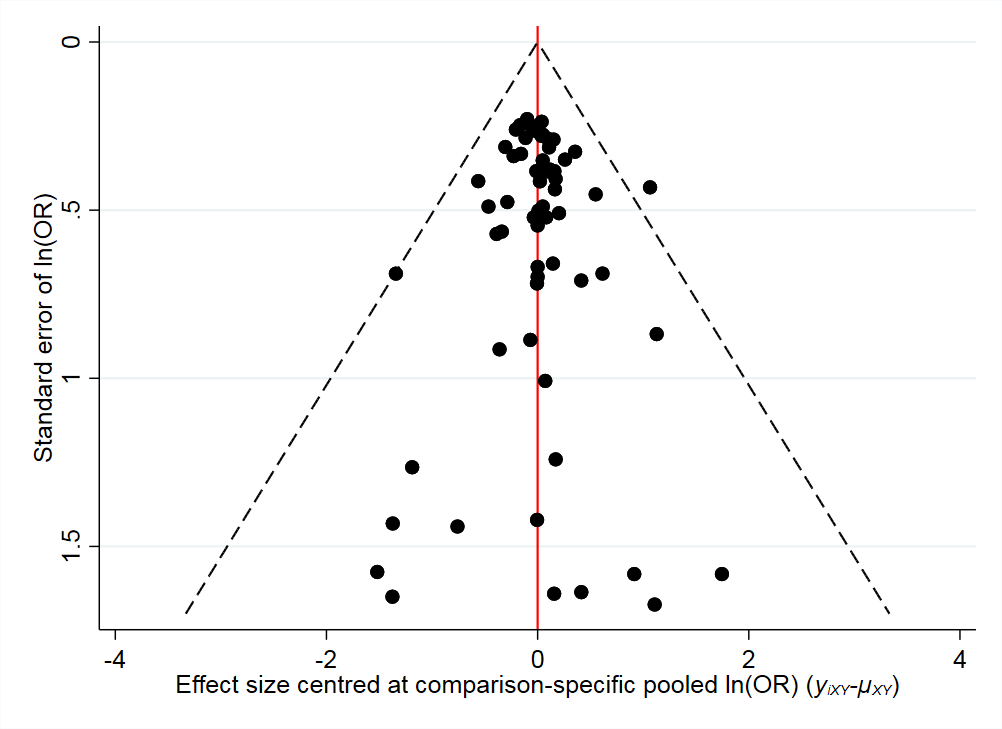


On visual inspection, there is no evidence of publication bias or small-study effects.

# Figure 2h. Comparison-Adjusted Funnel Plot: RCTs + NRSs Reporting Falls


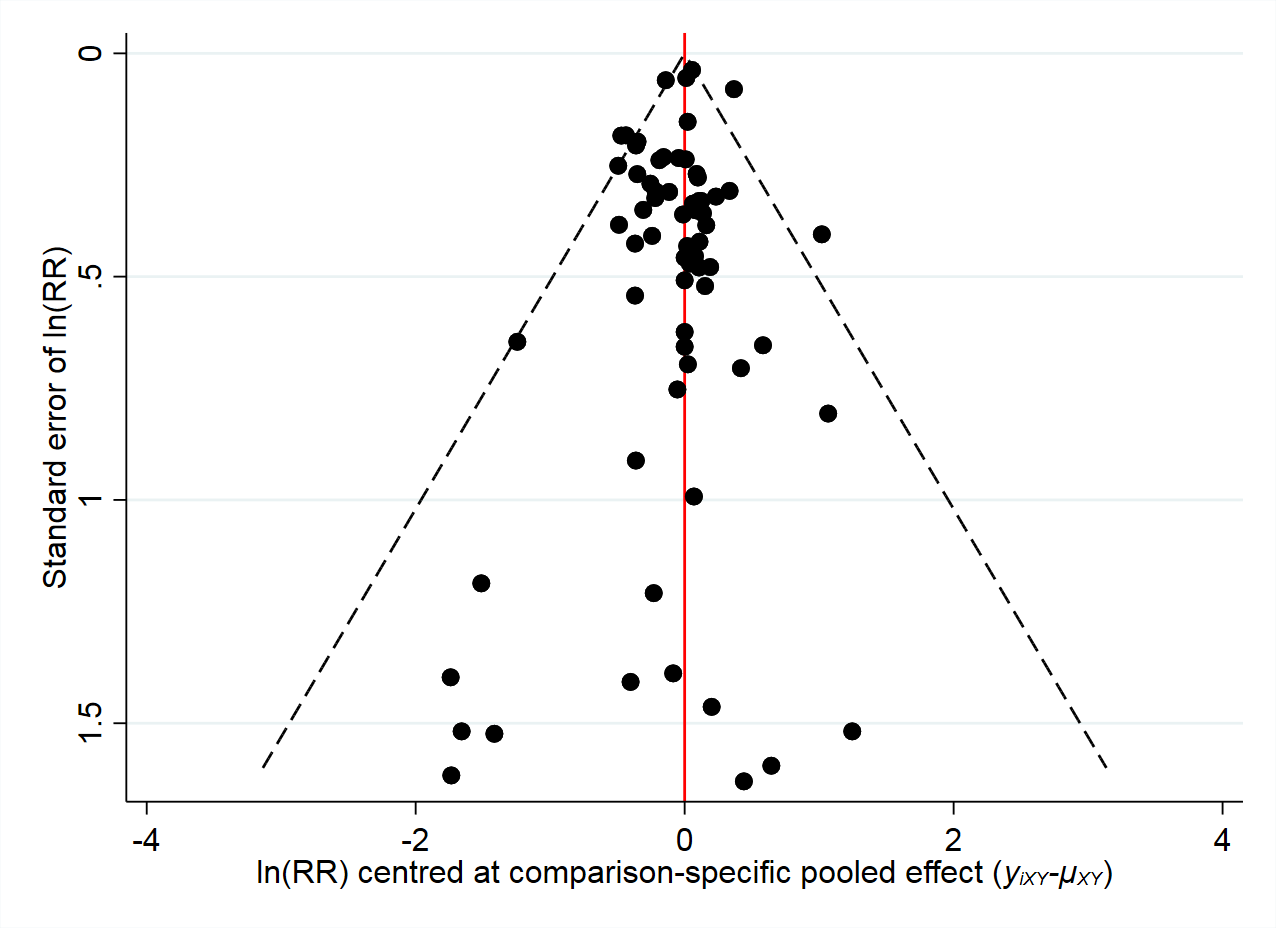


On visual inspection, there is no evidence of publication bias or small-study effects.

# Figure 3a. Inconsistency Plot: RCTs Reporting Fracture

No closed loops; therefore, there is no inconsistency plot.

# Figure 3b. Inconsistency Plot: RCTs + NRSs Reporting Fracture


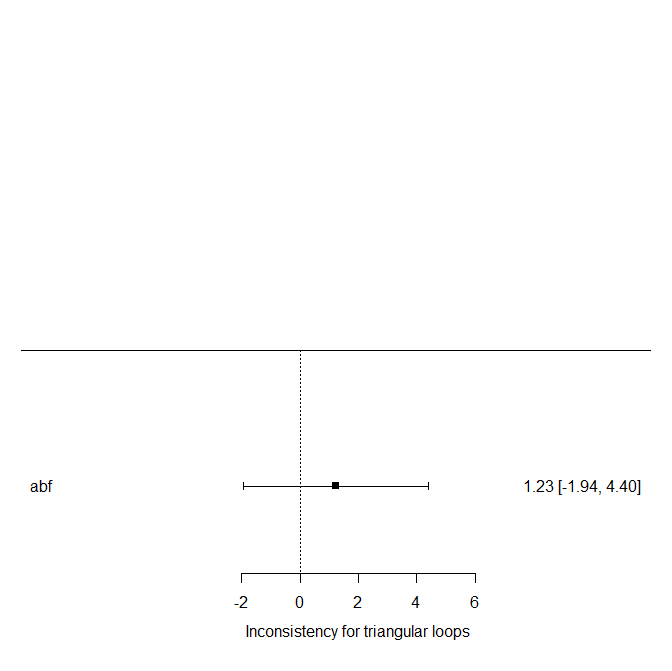


Abbreviations: placebo (a), antidepressants (b), antipsychotics (f)

There is no evidence of local inconsistency in the closed network loop.

# Figure 3c. Inconsistency Plot: RCTs Reporting Mortality


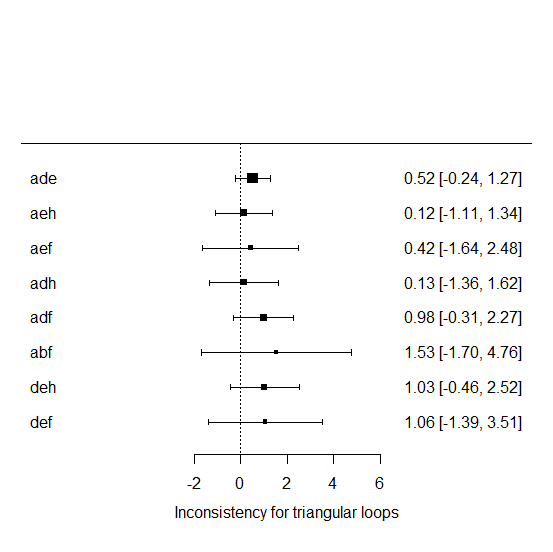


Abbreviations: placebo (a), antidepressants (b), memantine (d), cholinesterase inhibitors (e), antipsychotics (f), cholinesterase inhibitor + memantine (h)

There is no evidence of local inconsistency in any of the closed network loops.

# Figure 3d. Inconsistency Plot: RCTs + NRSs Reporting Mortality


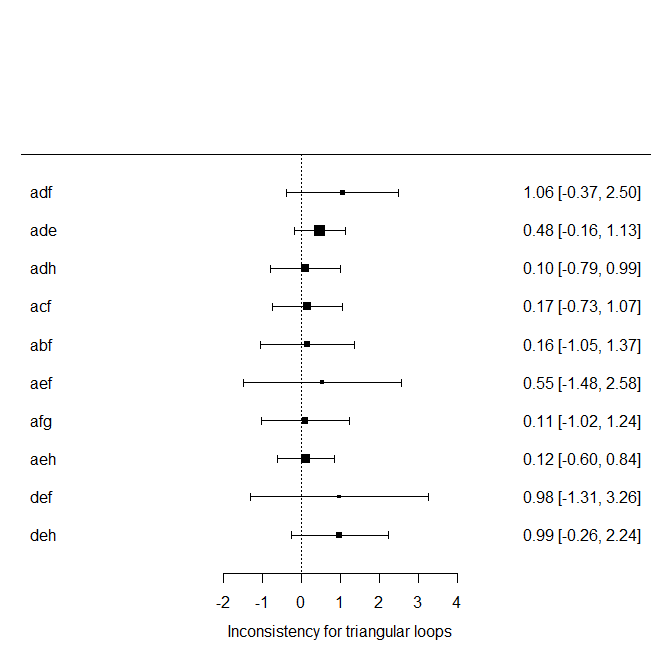


Abbreviations: placebo (a), antidepressants (b), anticonvulsants (c), memantine (d), cholinesterase inhibitors (e), antipsychotics (f), anxiolytic/hypnotic (g), cholinesterase inhibitor + memantine (h)

There is no evidence of local inconsistency in any of the closed network loops.

# Figure 3e. Inconsistency Plot: RCTs Reporting Cerebrovascular Event


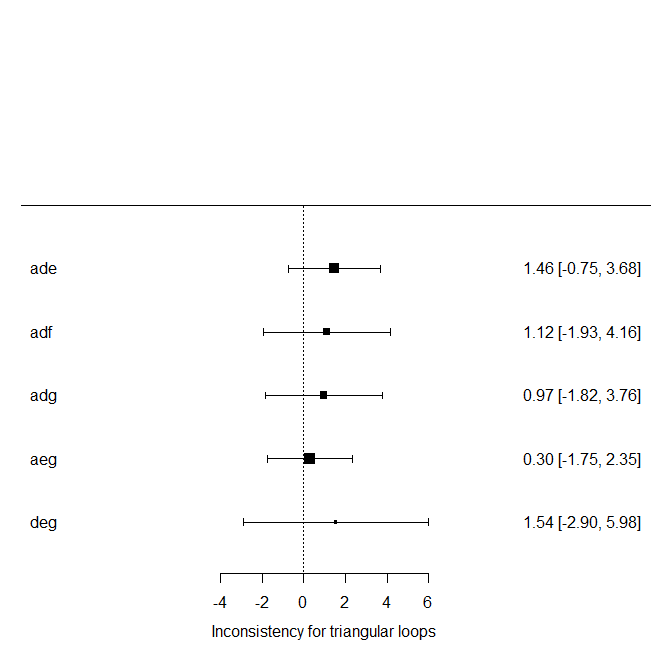


Abbreviations: placebo (a), memantine (d), cholinesterase inhibitors (e), antipsychotics (f), cholinesterase inhibitors + memantine (g)

There is no evidence of local inconsistency in any of the closed network loops.

# Figure 3f. Inconsistency Plot: RCTs + NRSs Reporting Cerebrovascular Event


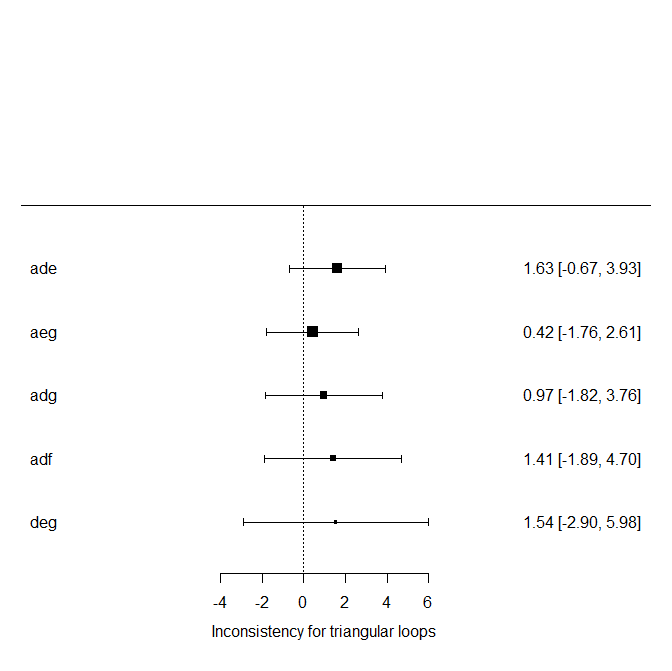


Abbreviations: placebo (a), memantine (d), anticonvulsants (e), antidepressants (f), cholinesterase inhibitors + memantine (g)

There is no evidence of local inconsistency in any of the closed network loops.

# Figure 3g. Inconsistency Plot: RCTs Reporting Falls


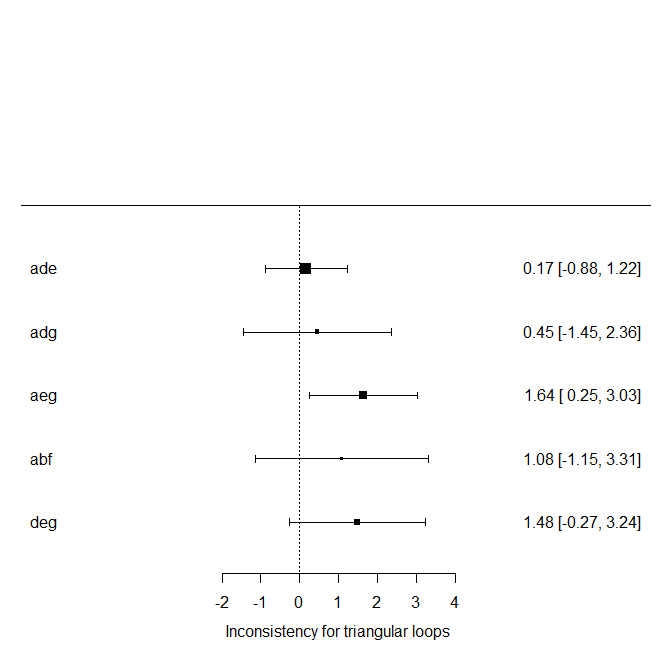


Abbreviations: placebo (a), antidepressants (b), memantine (d), cholinesterase inhibitors (e), antipsychotics (f), cholinesterase inhibitors + memantine (g)

There is one inconsistent network loop: placebo - antipsychotics - cholinesterase inhibitors+memantine.

# Figure 3h. Inconsistency Plot: RCTs + NRSs Reporting Falls


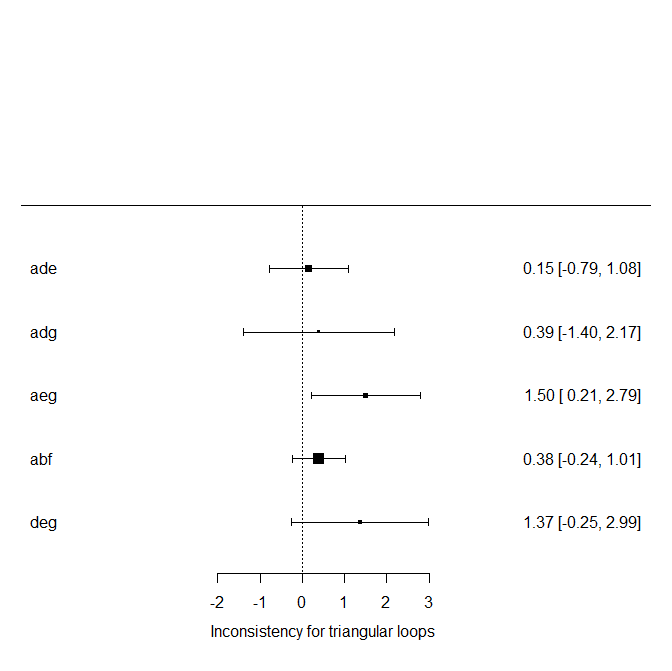


Abbreviations: placebo (a), antidepressants (b), memantine (d), cholinesterase inhibitors (e), anticonvulsants (f), cholinesterase inhibitors + memantine (g)

There is one inconsistent network loop: placebo - antipsychotics - cholinesterase inhibitors+memantine.

# References

[1] Watt J, Goodarzi Z, Tricco AC, Veroniki A-A, Straus SE. Comparative safety and efficacy of pharmacological and non-pharmacological interventions for the behavioral and psychological symptoms of dementia: protocol for a systematic review and network meta-analysis. Systematic Reviews. 2017;6:1-9.

[2] Turner RM, Davey J, Clarke MJ, Thompson SG, Higgins JP. Predicting the extent of heterogeneity in meta-analysis, using empirical data from the Cochrane Database of Systematic Reviews. Int J Epidemiol. 2012;41:818-27.

[3] Zarin W, Veroniki AA, Nincic V, Vafaei A, Reynen E, Motiwala SS, et al. Characteristics and knowledge synthesis approach for 456 network meta-analyses: a scoping review. BMC Med. 2017;15:3.

[4] Schneider LS, Dagerman KS, Insel P. Risk of Death with Atypical Antipsychotic Drug Treatment for Dementia: Meta-Analysis of Rnadomized Placebo-Controlled Trials. JAMA. 2005;294:1934-43.

[5] Aarsland D, Ballard C, Walker Z, Bostrom F, Alves G, Kossakowski K, et al. Memantine in patients with Parkinson's disease dementia or dementia with Lewy Bodies: a double-blind, placebo-controlled, multicentre trial. Lancet Neurol. 2009;8:613-18.

[6] Group AC. Long-term donepezil treatment in 565 patients with Alzheimer's disease (AD2000): randomised, double-blind trial. Lancet. 2004;363:2105-15.

[7] Aguglia E, Onor ML, Saina M, Maso E. An open-label, comparative study of rivastigmine, donepezil and galantamine in a real-world setting. Curr Med Res Opin. 2004;20:1747-52.

[8] Allain H, Dautzenberg PHJ, Maurer K, Schuck K, Bonhomme D, Gerard D. Double blind study of tiapride versus haloperidol and placebo in agitation and aggressiveness in elderly patients with cognitive impairment. Psychopharmacology. 2000;148:361-6.

[9] Ancoli-Israel S, Amatniek J, Ascher S, Sadik K, Ramaswamy K. Effects of Galantamine Versus Donepezil on Sleep in Patients with Mild to Moderate Alzheimer Disease and Their Caregivers. Alzheimer Dis Assoc Disord. 2005;19.

[10] Auchus AP, Brashear HR, Salloway S, Korczyn AD, De Deyn PP, Gassman-Mayer C. Galantamine treatment of vascular dementia. Neurology. 2007;69:448-58.

[11] Birks JS, Chong LY, Grimley Evans J. Rivastigmine for Alzheimer's disease (Review). Cochrane Database of Systematic Reviews. 2015.

[12] Bakchine S, Loft H. Memantine Treatment in Patients with Mild to Moderate Alzheimer's Disease: Results of a Randomised, Double-Blind, Placebo-Controlled 6-Month Study. Journal of Alzheimer's Disease. 2007;11:471-9.

[13] Ballard C, Thomas A, Fossey J, Jacoby R, Lana MM, Bannister C, et al. A 3-Month, Randomized, Placebo-Controlled, Neuroleptic Discontinuation Study in 100 People with Dementia: The Neuropsychiatric Inventory Median Cutoff is a Predictor of Clinical Outcome. J Clin Psychiatry. 2004;65:114-9.

[14] Ballard C, Margallo-Lana M, Juszczak E, Douglas S, Swann A, Thomas A, et al. Quetiapine and rivastigmine and cognitive decline in Alzheimer's disease: randomised double blind placebo controlled trial. BMJ. 2005;330:874.

[15] Ballard C, Sauter M, Scheltens P, He Y, Barkhof F, van Straaten EC, et al. Efficacy, safety and tolerability of rivastigmine capsules in patients with probable vascular dementia: the VantagE study. Curr Med Res Opin. 2008;24:2561-74.

[16] Ballard C, Thomas A, Gerry S, Yu LM, Aarsland D, Merritt C, et al. A double-blind randomized placebo-controlled withdrawal trial comparing memantine and antipsychotics for the long-term treatment of function and neuropsychiatric symptoms in people with Alzheimer's disease (MAIN-AD). J Am Med Dir Assoc. 2015;16:316-22.

[17] Ballard C, Banister C, Khan Z, Cummings J, Demos G, Coate B, et al. Evaluation of the safety, tolerability, and efficacy of pimavanserin versus placebo in patients with Alzheimer's disease psychosis: a phase 2, randomised, placebo-controlled, double-blind study. The Lancet Neurology. 2018;17:213-22.

[18] Banerjee S, Hellier J, Dewey M, Romeo R, Ballard C, Baldwin R, et al. Sertraline or mirtazapine for depression in dementia (HTA-SADD): a randomised, multicentre, double-blind, placebo-controlled trial. Lancet. 2011;378:403-11.

[19] Barak Y, Plopski I, Tadger S, Paleacu D. Escitalopram versus risperidone for the treatment of behavioral and psychotic symptoms associated with Alzheimer's disease: a randomized double-blind pilot study. Int Psychogeriatr. 2011;23:1515-9.

[20] Barnett MJ, Wehring H, Perry PJ. Comparison of risk of cerebrovascular events in an elderly VA population with dementia between antipsychotic and nonantipsychotic users. J Clin Psychopharmacol. 2007;27:595-601.

[21] Baxer AL, knopman DS, Kaufer DI, Grossman M, Onyike C, Graf-Radford N, et al. Memantine in patients with frontotemporal lobar degeneration: a multicentre, randomised, double-blind, placebo-controlled trial. Lancet Neurol. 2013;12:149-56.

[22] Black S, Roman GC, Geldmacher DS, Salloway S, Hecker J, Burns A, et al. Efficacy and tolerability of donepezil in vascular dementia: positive results of a 24-week, multicenter, international, randomized, placebo-controlled clinical trial. Stroke. 2003;34:2323-30.

[23] Black SE, Doody R, Li H, McRae T, Jambor KM, Xu Y, et al. Donepezil preserves cognition and global function in patients with severe Alzheimer's disease. Neurology. 2007;69:459-69.

[24] Brodaty H, Ames D, Snowdon J, Woodward M, Kirwan J, Clarnette R, et al. A Randomized Placebo-Controlled Trial of Risperidone for the Treatment of Aggression, Agitation, and Psychosis of Dementia. J Clin Psychiatry. 2003;64:134-43.

[25] Brodaty H, Corey-Bloom J, Potocnik FC, Truyen L, Gold M, Damaraju CR. Galantamine prolonged-release formulation in the treatment of mild to moderate Alzheimer's disease. Dement Geriatr Cogn Disord. 2005;20:120-32.

[26] Bronskill SE, Campitelli MA, Iaboni A, Herrmann N, Guan J, Maclagan LC, et al. Low-Dose Trazodone, Benzodiazepines, and Fall-Related Injuries in Nursing Homes: A Matched-Cohort Study. J Am Geriatr Soc. 2018;66:1963-71.

[27] Bullock R, Touchon J, Bergman H, Gambina G, He Y, Rapatz G, et al. Rivastigmine and donepezil treatment in moderate to moderately-severe Alzheimer's disease over a 2-year period. Curr Med Res Opin. 2005;21:1317-27.

[28] Burns A, Rossor M, Hecker J, Gauthier S, Petit H, Moller H-J, et al. The Effects of Donepezil in Alzheimer's Disease - Results from a Multinational Trial. Dement Geriatr Cogn Disord. 1999;10:234-44.

[29] Burns A, Bernabei R, Bullock R, Jentoft AJC, Frölich L, Hock C, et al. Safety and efficacy of galantamine (Reminyl) in severe Alzheimer's disease (the SERAD study): a randomised, placebo-controlled, double-blind trial. The Lancet Neurology. 2009;8:39-47.

[30] Camargos EF, Louzada LL, Quintas JL, Naves JO, Louzada FM, Nobrega OT. Trazodone improves sleep parameters in Alzheimer disease patients: a randomized, double-blind, and placebo-controlled study. Am J Geriatr Psychiatry. 2014;22:1565-74.

[31] Campbell NL, Perkins AJ, Gao S, Skaar TC, Li L, Hendrie HC, et al. Adherence and Tolerability of Alzheimer's Disease Medications: A Pragmatic Randomized Trial. J Am Geriatr Soc. 2017;65:1497-504.

[32] Carlyle W, Ancill RJ, Sheldon L. Aggression in the demented patient: a double-blind study of loxapine versus haloperidol. International Clinical Psychopharmacology. 1993;8:103-8.

[33] Chan W-c, Lam LC-w, Choy CN-p, Leung VP-y, Li S-w, Chiu HF-k. A double-blind randomised comparison of risperidone and haloperidol in the treatment of behavioural and psychological symptoms in Chinese dementia patients. International Journal of Geriatric Psychiatry. 2001;16.

[34] Chan MC, Chong CS, Wu AY, Wong KC, Dunn EL, Tang OW, et al. Antipsychotics and risk of cerebrovascular events in treatment of behavioural and psychological symptoms of dementia in Hong Kong: a hospital-based, retrospective, cohort study. Int J Geriatr Psychiatry. 2010;25:362-70.

[35] Choe YM, Kim KW, Jhoo JH, Ryu SH, Seo EH, Sohn BK, et al. Multicenter, randomized, placebo-controlled, double-blind clinical trial of escitalopram on the progression-delaying effects in Alzheimer's disease. Int J Geriatr Psychiatry. 2016;31:731-9.

[36] Choi SH, Park KW, Na DL, Han HJ, Kim EJ, Shim YS, et al. Tolerability and efficacy of memantine add-on therapy to rivastigmine transdermal patches in mild to moderate Alzheimer's disease: a multicenter, randomized, open-label, parallel-group study. Curr Med Res Opin. 2011;27:1375-83.

[37] Corey-Bloom J, Anand R, Veach J. A randomized trial evaluating the efficacy and safety of ENA 713 (rivastigmine tartrate), a new acetylcholinesterase inhibitor, in patients with mild to moderately severe Alzheimer's disease. International Journal of Geriatric Psychopharmacology. 1998;1:55-65.

[38] Culo S, Mulsant BH, Rosen J, Mazumdar S, Blakesley RE, Houck PR, et al. Treating Neuropsychiatric Symptoms in Dementia with Lewy Bodies: A Randomized Controlled-trial. Alzheimer Dis Assoc Disord. 2010;24:360-4.

[39] Cumbo E, Ligori LD. Differential effects of current specific treatments on behavioral and psychological symptoms in patients with Alzheimer's disease: a 12-month, randomized, open-label trial. J Alzheimers Dis. 2014;39:477-85.

[40] Cummings J, Froelich L, Black SE, Bakchine S, Bellelli G, Molinuevo JL, et al. Randomized, double-blind, parallel-group, 48-week study for efficacy and safety of a higher-dose rivastigmine patch (15 vs. 10 cm(2)) in Alzheimer's disease. Dement Geriatr Cogn Disord. 2012;33:341-53.

[41] Cummings JL, Lyketsos CG, Peskind ER, Porsteinsson AP, Mintzer JE, Scharre DW, et al. Effect of Dextromethorphan-Quinidine on Agitation in Patients With Alzheimer Disease Dementia: A Randomized Clinical Trial. JAMA. 2015;314:1242-54.

[42] De Deyn PP, Rabheru K, Rasmussen A, Bocksberger JP, Dautzenberg PHJ, Eriksson S, et al. A randomized trial of risperidone, placebo, and haloperidol for behavioral symptoms of dementia. Neurology. 1999;53:946-55.

[43] De Deyn PP, Carrasco MM, Deberdt W, Jeandel C, Hay DP, Feldman PD, et al. Olanzapine versus placebo in the treatment of psychosis with or without associated behavioral disturbances in patients with Alzheimer's disease. Int J Geriatr Psychiatry. 2004;19:115-26.

[44] De Deyn P, Jeste DV, Swanink R, Kostic D, Breder C. Aripiprazole for the Treatment of Psychosis in Patients With Alzheimer's Disease. Journal of Clinical Psychopharmacology. 2005;25:463-7.

[45] de Vasconcelos Cunha UG, Lopes Rocha F, Avila de Melo R, Alves Valle E, de Souza Neto JJ, Mendes Brega R, et al. A placebo-controlled double-blind randomized study of venlafaxine in the treatment of depression in dementia. Dement Geriatr Cogn Disord. 2007;24:36-41.

[46] Deberdt W, Dysken MW, Rappaport SA, Feldman PD, Young CA, Hay DP, et al. Comparison of Olanzapine and Risperidone in the Treatment of Psychosis and Associated Behavioral Disturbances in Patients with Dementia. American Journal of Geriatric Psychiatry. 2005;13:722-30.

[47] Devanand DP, Mintzer J, Schultz SK, Andrews HF, Sultzer DL, de la Pena D, et al. Relapse risk after discontinuation of risperidone in Alzheimer's disease. N Engl J Med. 2012;367:1497-507.

[48] Kim DH, Brown RT, Ding EL, Kiel DP, Berry SD. Dementia medications and risk of falls, syncope, and related adverse events: meta-analysis of randomized controlled trials. J Am Geriatr Soc. 2011;59:1019-31.

[49] Doody RS, Geldmacher DS, Farlow MR, Sun Y, Moline M, Mackell J. Efficacy and safety of donepezil 23 mg versus donepezil 10 mg for moderate-to-severe Alzheimer's disease: a subgroup analysis in patients already taking or not taking concomitant memantine. Dement Geriatr Cogn Disord. 2012;33:164-73.

[50] Dubois B, Tolosa E, Katzenschlager R, Emre M, Lees AJ, Schumann G, et al. Donepezil in Parkinson's disease dementia: a randomized, double-blind efficacy and safety study. Mov Disord. 2012;27:1230-8.

[51] Dysken MW, Sano M, Asthana S, Vertrees JE, Pallaki M, Llorente M, et al. Effect of vitamin E and memantine on functional decline in Alzheimer disease: the TEAM-AD VA cooperative randomized trial. JAMA. 2014;311:33-44.

[52] Eady N, Sheehan R, Rantell K, Sinai A, Bernal J, Bohnen I, et al. Impact of cholinesterase inhibitors or memantine on survival in adults with Down syndrome and dementia: clinical cohort study. Br J Psychiatry. 2018;212:155-60.

[53] Emre M, Aarsland D, Albanese A, Byrne EJ, Deuschl G, De Deyn PP, et al. Rivastigmine for Dementia Associated with Parkinson's Disease. NEJM. 2004;351:2509-18.

[54] Emre M, Tsolaki M, Bonuccelli U, Destee A, Tolosa E, Kutzelnigg A, et al. Memantine for patients with Parkinson's disease dementia or dementia with Lewy bodies: a randomised, double-blind, placebo-controlled trial. Lancet Neurology. 2010;9:969-77.

[55] Emre M, Poewe W, De Deyn PP, Barone P, Kulisevsky J, Pourcher E, et al. Long-term safety of rivastigmine in parkinson disease dementia: an open-label, randomized study. Clin Neuropharmacol. 2014;37:9-16.

[56] Erkinjuntti T, Kurz A, Gauthier S, Bullock R, Lillienfeld S, Rao C, et al. Efficacy of galantamine in probable vascular dementia and Alzheimer's disease combined with cerebrovascular disease: a randomised trial. Lancet. 2002;359:1283-90.

[57] Farlow MR, Alva G, Meng X, Olin JT. A 25-week, open-label trial investigating rivastigmine transdermal patches with concomitant memantine in mild-to-moderate Alzheimer's disease: a post hoc analysis. Curr Med Res Opin. 2010;26:263-9.

[58] Farlow MR, Grossberg GT, Sadowsky CH, Meng X, Somogyi M. A 24-week, randomized, controlled trial of rivastigmine patch 13.3 mg/24 h versus 4.6 mg/24 h in severe Alzheimer's dementia. CNS Neurosci Ther. 2013;19:745-52.

[59] Feldman H, Gauthier S, Hecker J, Vellas B, Subbiah P, Whalen E. A 24-week, randomized, double-blind study of donepezil in moderate to severe Alzheimer's disease. Neurology. 2001;57:613-20.

[60] Feldman HH, Lane R, Study G. Rivastigmine: a placebo controlled trial of twice daily and three times daily regimens in patients with Alzheimer's disease. J Neurol Neurosurg Psychiatry. 2007;78:1056-63.

[61] Finkel SI, Lyons JS, Anderson RL, Sherrell K. A Randomized, Placebo-Controlled Trial of Thioxene in Agitated, Demented Nursing Home Patients. International Journal of Geriatric Psychiatry. 1995;10:129-36.

[62] Finkel S, Kozma C, Long S, Greenspan A, Mahmoud R, Baser O, et al. Risperidone treatment in elderly patients with dementia: relative risk of cerebrovascular events versus other antipsychotics. Int Psychogeriatr. 2005;17:617-29.

[63] Fontaine CS, Hynan LS, Koch K, Martin-Cook K, Svetlik D, Weiner MF. A Double-Blind Comparison of Olanzapine Versus Risperidone in the Acute Treatment of Dementia-Related Behavioral Disturbances in Extended Care Facilities. J Clin Psychiatry. 2003;64:726-30.

[64] Fox C, Crugel M, Maidment I, Auestad BH, Coulton S, Treloar A, et al. Efficacy of memantine for agitation in Alzheimer's dementia: a randomised double-blind placebo controlled trial. PLoS One. 2012;7:e35185.

[65] Feldman HH, Pirttila T, Dartigues JF, Everitt B, Van Baelen B, Brashear HR, et al. Analyses of mortality risk in patients with dementia treated with galantamine. Acta Neurol Scand. 2009;119:22-31.

[66] Loy C, Schneider L. Galantamine for Alzheimer's disease and mild cognitive impairment. Cochrane Database Syst Rev. 2006:CD001747.

[67] Gasper MC, Ott BR, Lapane KL. Is Donepezil Therapy Associated with Reduced Mortality in Nursing Home Residents with Dementia? The American Journal of Geriatric Pharmacotherapy. 2005;3:1-7.

[68] Gault LM, Lenz RA, Ritchie CW, Meier A, Othman AA, Tang Q, et al. ABT-126 monotherapy in mild-to-moderate Alzheimer's dementia: randomized double-blind, placebo and active controlled adaptive trial and open-label extension. Alzheimer's Research and Therapy. 2016;8:1-13.

[69] Gerhard T, Huybrechts K, Olfson M, Schneeweiss S, Bobo WV, Doraiswamy PM, et al. Comparative mortality risks of antipsychotic medications in community-dwelling older adults. Br J Psychiatry. 2014;205:44-51.

[70] Gill SS, Rochon PA, Herrmann N, Lee PE, Sykora K, Gunraj N, et al. Atypical antipsychotic drugs and risk of ischaemic stroke: population based retrospective cohort study. BMJ. 2005;330:445.

[71] Gill SS, Bronskill SE, Normand S-LT, Anderson GM, Sykora K, Lam K, et al. Antipsychotic Drug Use and Mortality in Older Adults with Dementia. Annals of Internal Medicine. 2007;146:775-86.

[72] Gill SS, Anderson GM, Fischer HD, Bell CM, Li P, Normand S-LT, et al. Syncope and Its Consequences in Patients with Dementia Receiving Cholinesterase Inhibitors: A Population-Based Cohort Study. Arch Intern Med. 2009;169:867-73.

[73] Gold M, Alderton C, Zvartau-Hind M, Egginton S, Saunders AM, Irizarry M, et al. Rosiglitazone monotherapy in mild-to-moderate Alzheimer's disease: results from a randomized, double-blind, placebo-controlled phase III study. Dement Geriatr Cogn Disord. 2010;30:131-46.

[74] Grossberg G, Manes F, Allegri RF, Gutierrez-Robledo LM, Gloger S, Xie L, et al. The Safety, Tolerability, and Efficacy of Once-Daily Memantine (28mg): A Multinational, Randomized, Double-Blind, Placebo-Controlled Trial in Patients with Moderate-to-Severe Alzheimer's Disease Taking Cholinesterase Inhibitors. CNS Drugs. 2013;27:469-78.

[75] Hager K, Baseman AS, Nye JS, Brashear HR, Han J, Sano M, et al. Effects of galantamine in a 2-year, randomized, placebo-controlled study in Alzheimer's disease. Neuropsychiatr Dis Treat. 2014;10:391-401.

[76] Hampel H, Ewers M, Burger K, Annas P, Mortberg A, Bogstedt A, et al. Lithium Trial in Alzheimer's Disease: A Randomized, Single-Blind, Placebo-Controlled, Multicneter 10-Week Study. J Clin Psychiatry. 2009;70:922-31.

[77] Herrmann N, Lanctot KL, Rothenburg LS, Eryavec G. A placebo-controlled trial of valproate for agitation and aggression in Alzheimer's disease. Dement Geriatr Cogn Disord. 2007;23:116-9.

[78] Herrmann N, Gauthier S, Boneva N, Lemming OM, Investigators. A randomized, double-blind, placebo-controlled trial of memantine in a behaviorally enriched sample of patients with moderate-to-severe Alzheimer's disease. Int Psychogeriatr. 2013;25:919-27.

[79] Herrmann N, O'Regan J, Ruthirakuhan M, Kiss A, Eryavec G, Williams E, et al. A Randomized Placebo-Controlled Discontinuation Study of Cholinesterase Inhibitors in Institutionalized Patients With Moderate to Severe Alzheimer Disease. J Am Med Dir Assoc. 2016;17:142-7.

[80] Homma A, Imai Y, S. H, al. e. Late phase Ii clinical study of acetylcholinesterase inhibitor E 2020 in patients with Alzheimer-type dementia-12-weeks double-blind, placebo-controlled study 3mg/day, 5mg/day. Clin Eval. 1998;26:251-84.

[81] Homma A, Takeda M, Imai Y, Udaka F, Hasegawa K, Kameyama M, et al. Clinical Efficacy and Safety of Donepezil on Cognitive and Global Function in Patients with Alzheimer's Disease: A 24-Week, Multicenter, Double-Blind, Placebo-Controlled Study in Japan. Dement Geriatr Cogn Dis 2000;11:299-313.

[82] Homma A, Imai Y, Tago H, Asada T, Shigeta M, Iwamoto T, et al. Donepezil treatment of patients with severe Alzheimer's disease in a Japanese population: results from a 24-week, double-blind, placebo-controlled, randomized trial. Dement Geriatr Cogn Disord. 2008;25:399-407.

[83] Howard R, Juszczak E, Ballard CG, Bentham P, Brown RG, Bullock R, et al. Donepezil for the Treatment of Agitation in Alzheimer's Disease. NEJM. 2007;357:1382-92.

[84] Howard R, McShane R, Lindesay J, Ritchie C, Baldwin A, Barber R, et al. Donepezil and Memantine for Moderate-to-Severe Alzheimer's Disease. NEJM. 2012;366:893-903.

[85] Hu H-t, Zhang Z-x, Yao J-l, Yu H-z, Wang Y-h, Tang H-c, et al. Clinical efficacy and safety of akatinol memantine in treatment of mild to moderate Alzheimer disease: a donepezil-controlled, randomized trial. Chin J Intern Med. 2006;45:277-80.

[86] Huybrechts KF, Gerhard T, Crystal S, Olfson M, Avorn J, Levin R, et al. Differential risk of death in older residents in nursing homes prescribed specific antipsychotic drugs: population based cohort study. BMJ. 2012;344:e977.

[87] Jalbert JJ, Eaton CB, Miller SC, Lapane KL. Antipsychotic use and the risk of hip fracture among older adults afflicted with dementia. J Am Med Dir Assoc. 2010;11:120-7.

[88] Jia J, Wei C, Jia L, Tang Y, Liang J, Zhou A, et al. Efficacy and Safety of Donepezil in Chinese Patients with Severe Alzheimer's Disease: A Randomized Controlled Trial. J Alzheimers Dis. 2017;56:1495-504.

[89] Johannsen P, Salmon E, Hampel H, Xu Y, Richardson S, Qvitzau S, et al. Assessing Therapeutic Efficacy in a Progressive Disease: A Study of Donepezil in Alzheimer's disease. CNS Drugs. 2006;20:311-25.

[90] Kales HC, Valenstein M, Kim HM, McCarthy JF, Ganoczy D, Cunningham F, et al. Mortality Risk in Patients with Dementia Treated with Antipsychotics Versus Other Psychiatric Medications. American Journal of Psychiatry. 2007;164:1568-76.

[91] Kales HC, Kim HM, Zivin K, Valenstein M, Seyfried LS, Chiang C, et al. Risk of Mortality Among Individual Antipsychotics in Patients with Dementia. American Journal of Psychiatry. 2012;169:71-9.

[92] Katona CLE, Hunter BN, Bray J. A Double-Blind Comparison of the Efficacy and Safety of Paroxetine and Imipramine in the Treatment of Depression with Dementia. International Journal of Geriatric Psychiatry. 1998;13:100-8.

[93] Katz IR, Jeste DV, Mintzer J, Clyde C, Napolitano J, Brecher M. Comparison of Risperidone and Placebo for Psychosis and Behavioral Disturbances Associated with Dementia: A Randomized, Double-Blind Trial. J Clin Psychiatry. 1999;60:107-15.

[94] Katz IR, Rupnow M, Kozma C, Schneider LS. Risperidone and Falls in Ambulatory Nursing Home Residents With Dementia and Psychosis or Agitation: Secondary Analysis of a Double-Blind, Placebo-Controlled Trial. Am J Geriatr Psychiatry. 2004;12:499-508.

[95] Kennedy J, Deberdt W, Siegal A, Micca J, Degenhardt E, Ahl J, et al. Olanzapine does not enhance cognition in non-agitated and non-psychotic patients with mild to moderate Alzheimer's dementia. Int J Geriatr Psychiatry. 2005;20:1020-7.

[96] Kertesz A, Morlog D, Light M, Blair M, Davidson W, Jesso S, et al. Galantamine in frontotemporal dementia and primary progressive aphasia. Dement Geriatr Cogn Disord. 2008;25:178-85.

[97] Kheirbek RE, Fokar A, Little JT, Balish M, Shara NM, Boustani MA, et al. Association Between Antipsychotics and All-Cause Mortality Among Community-Dwelling Older Adults. J Gerontol A Biol Sci Med Sci. 2019.

[98] Lanctot KL, Herrmann N, van Reekum R, Eryavec G, Naranjo CA. Gender, aggression and serotonergic function are associated with response to sertraline for behavioral disturbances in Alzheimer's disease. Int J Geriatr Psychiatry. 2002;17:531-41.

[99] Langballe EM, Engdahl B, Nordeng H, Ballard C, Aarsland D, Selbaek G. Short- and long-term mortality risk associated with the use of antipsychotics among 26,940 dementia outpatients: a population-based study. Am J Geriatr Psychiatry. 2014;22:321-31.

[100] Layton D, Harris S, Wilton LV, Shakir SAW. Comparison of incidence rates of cerebrovascular accidents and transient ischemic attacks in observational cohort studies of patients prescribed risperidone, quetiapine, or olanzapine in general practice in England including patients with dementia. Journal of Psychopharmacology. 2005;19:473-82.

[101] Lee KJ, Cho S-J, Kim BC, Park M, Lee J-H. Caregiver Preference and Treatment Compliance in Patients with Mild-to-Moderate Alzheimer's Disease in South Korea: RECAP Study Results. Adv Ther. 2017;34:481-94.

[102] Lin YT, Wu PH, Chen CS, Yang YH, Yang YH. Association between acetylcholinesterase inhibitors and risk of stroke in patients with dementia. Sci Rep. 2016;6:29266.

[103] Liperoti R, Gambassi G, Lapane KL, Chiang C, Pedone C, Mor V, et al. Cerebrovascular Events Among Elderly Nursing Home Patients Treated with Conventional or Atypical Antipsychotics. J Clin Psychiatry. 2005;66:1090-6.

[104] Liperoti R, Onder G, Landi F, Lapane KL, Mor V, Bernabei R, et al. All-Cause Mortality Associated with Atypical and Conventional Antipsychotics Among Nursing Home Residents with Dementia: A Retrospective Cohort Study. J Clin Psychiatry. 2009;70:1340-7.

[105] Litvinenko IV, Odinak MM, Mogil'naya VI, Emelin AY. Efficacy and Safety of Galantamine (reminyl) for Dementia in Patients with Parkinson's Disease (an open controlled trial). Neuroscience and Behavioral Physiology. 2008;38:937-45.

[106] Lopez OL, Wisniewski SR, Becker JT, Boller F, DeKosky ST. Psychiatric Medication and Abnormal Behaviour as Predictors of Progression in Probable Alzheimer's Disease. Arch Neurol. 1999;1999:1266-72.

[107] Lopez OL, Becker JT, Wahed AS, Saxton J, Sweet RA, Wolk DA, et al. Long-term effects of the concomitant use of memantine with cholinesterase inhibition in Alzheimer disease. J Neurol Neurosurg Psychiatry. 2009;80:600-7.

[108] Lopez-Pousa S, Olmo JG, Franch JV, Estrada AT, Cors OS, Nierga IP, et al. Comparative analysis of mortality in patients with Alzheimer's disease treated with donepezil or galantamine. Age Ageing. 2006;35:365-71.

[109] Lyketsos CG, DelCampo L, Steinberg M, Miles Q, Steele CD, Munro C, et al. Treating Depression in Alzheimer Disease: Efficacy and Safety of Sertraline Therapy, and the Benefits of Depression Reduction: The DIADS. Arch Gen Psychiatry. 2003;60:737-46.

[110] Maher-Edwards G, Dixon R, Hunter J, Gold M, Hopton G, Jacobs G, et al. SB-742457 and donepezil in Alzheimer disease: a randomized, placebo-controlled study. Int J Geriatr Psychiatry. 2011;26:536-44.

[111] Martin H, Slyk MP, Deymann S, Cornacchione MJ. Safety profile assessment of risperidone and olanzapine in long-term care patients with dementia. J Am Med Dir Assoc. 2003;4:183-8.

[112] Martinez Martinez L, Olivera Fernandez MR, Pineiro Corrales G. Mortality in patients with dementia treated with atypical antipsychotics (olanzapine, quetiapine, ziprasidone). Farmacia Hospitalaria. 2009;33:224-8.

[113] Maust DT, Kim HM, Seyfried LS, Chiang C, Kavanagh J, Schneider LS, et al. Antipsychotics, other psychotropics, and the risk of death in patients with dementia: number needed to harm. JAMA Psychiatry. 2015;72:438-45.

[114] McKeith I, Del Ser T, Spano P, Emre M, Wesnes K, Anand R, et al. Efficacy of rivastigmine in dementia with Lewy bodies: a randomised, double-blind, placebo-controlled international study. Lancet. 2000;356:2031-6.

[115] Mintzer J, Greenspan A, Caers I, Van Hove I, Kushner S, Weiner M, et al. Risperidone in the Treatment of Psychosis of Alzheimer Disease: Results from a Prospective Clinical Trial. American Journal of Geriatric Psychiatry. 2006;14:280-91.

[116] Mintzer J, Tune LE, Breder CD, Swanink R, Marcus R, McQuade R, et al. Aripiprazole for the Treatment of Psychoses in Institutionalized Patients with Alzheimer Dementia: A Multicenter, Randomized, Double-Blind, Placebo-Controlled Assessment of Three Fixed Doses. American Journal of Geriatric Psychiatry. 2007;15:918-31.

[117] Mohs RC, Doody R, Morris JC, Ieni JR, Rogers SL, Perdomo C, et al. A 1-year, placebo-controlled preservation of function survival study of donepezil in AD patients. Neurology. 2001;57:481-8.

[118] Mok V, Wong A, Ho S, Leung T, Lam WW, Wong KS. Rivastigmine in Chinese patients with subcortical vascular dementia. Neuropsychiatr Dis Treat. 2007;3:943-8.

[119] Moretti R, Torre P, Antonello RM, Cattaruzza T, Cazzato G. Olanzapine as a possible treatment of behavioral symptoms in vascular dementia: risks of cerebrovascular events. A controlled, open-label study. J Neurol. 2005;252:1186-93.

[120] Mueller C, Perera G, Hayes RD, Shetty H, Stewart R. Associations of acetylcholinesterase inhibitor treatment with reduced mortality in Alzheimer's disease: a retrospective survival analysis. Age Ageing. 2018;47:88-94.

[121] Musicco M, Palmer K, Russo A, Caltagirone C, Adorni F, Pettenati C, et al. Association between prescription of conventional or atypical antipsychotic drugs and mortality in older persons with Alzheimer's disease. Dement Geriatr Cogn Disord. 2011;31:218-24.

[122] Nakamura Y, Imai Y, Shigeta M, Graf A, Shirahase T, Kim H, et al. A 24-week, randomized, double-blind, placebo-controlled study to evaluate the efficacy, safety and tolerability of the rivastigmine patch in Japanese patients with Alzheimer's disease. Dement Geriatr Cogn Dis Extra. 2011;1:163-79.

[123] Nakamura Y, Kitamura S, Homma A, Shiosakai K, Matsui D. Efficacy and safety of memantine in patients with moderate-to-severe Alzheimer's disease: results of a pooled analysis of two randomized, double-blind, placebo-controlled trials in Japan. Expert Opin Pharmacother. 2014;15:913-25.

[124] Nordstrom P, Religa D, Wimo A, Winblad B, Eriksdotter M. The use of cholinesterase inhibitors and the risk of myocardial infarction and death: a nationwide cohort study in subjects with Alzheimer's disease. Eur Heart J. 2013;34:2585-91.

[125] Nyth AL, Gottfries CG. The Clinical Efficacy of Citalopram in Treatment of Emotional Disturbances in Dementia Disorders: A Nordic Multicentre Study. British Journal of Psychiatry. 1990;157:894-901.

[126] Orgogozo J-M, Rigaud A-S, Stoffler A, Mobius H-J, Forette F. Efficacy and Safety of Memantine in Patients with Mild to Moderate Vascular Dementia: A Randomized, Placebo-Controlled Trial (MMM 300). Stroke. 2002;33:1834-9.

[127] Pakdaman H, Harandi AA, Hatamian H, Tabatabae M, Delavar Kasmaei H, Ghassemi A, et al. Effectiveness and Safety of MLC601 in the Treatment of Mild to Moderate Alzheimer's Disease: A Multicenter, Randomized Controlled Trial. Dement Geriatr Cogn Dis Extra. 2015;5:96-106.

[128] Paleacu D, Barak Y, Mirecky I, Mazeh D. Quetiapine treatment for behavioural and psychological symptoms of dementia in Alzheimer's disease patients: a 6-week, double-blind, placebo-controlled study. Int J Geriatr Psychiatry. 2008;23:393-400.

[129] Peskind ER, Potkin SG, Pomara N, Ott BR, Graham SM, Olin JT, et al. Memantine Treatment in Mild to Moderate Alzheimer Disease: A 24-Week Randomized, Controlled Trial. American Journal of Geriatric Psychiatry. 2006;14:704-15.

[130] Peters O, Fuentes M, Joachim LK, Jessen F, Luckhaus C, Kornhuber J, et al. Combined treatment with memantine and galantamine-CR compared with galantamine-CR only in antidementia drug naive patients with mild-to-moderate Alzheimer's disease. Alzheimers Dement (N Y). 2015;1:198-204.

[131] Piersanti M, Capannolo M, Turchetti M, Serroni N, De Berardis D, Evangelista P, et al. Increase in mortality rate in patients with dementia treated with atypical antipsychotics: a cohort study in outpatients in Central Italy. Riv Psichiatr. 2014;49:34-40.

[132] Porsteinsson A, Tariot PN, Erb R, Cox C, Smith E, Jakimovich LJ, et al. Placebo-Controlled Study of Divalproex Sodium for Agitation in Dementia. Am J Geriatr Psychiatry. 2001;9:58-66.

[133] Porsteinsson AP, Grossberg G, Mintzer J, Olin JT. Memantine Treatment in Patients with Mild to Moderate Alzheimer's Disease Already Receiving a Cholinesterase Inhibitor: A Randomized, Double-Blind, Placebo-Controlled Trial. Current Alzheimer Research. 2008;5:83-9.

[134] Porsteinsson AP, Drye LT, Pollock BG, Devanand DP, Frangakis C, Ismail Z, et al. Effect of citalopram on agitation in Alzheimer disease: the CitAD randomized clinical trial. JAMA. 2014;311:682-91.

[135] Profenno LA, Jakimovich LJ, Holt CJ, Porsteinsson AP, Tariot PN. A Randomized, Double-Blind, Placebo-Controlled Pilot Trial of Safety and Tolerability of Two Doses of Divalproex Sodium in Outpatients with Probable Alzheimer's Disease. Current Alzheimer Research. 2005;2:553-8.

[136] Rafaniello C, Lombardo F, Ferrajolo C, Sportiello L, Parretta E, Formica R, et al. Predictors of mortality in atypical antipsychotic-treated community-dwelling elderly patients with behavioral and psychological symptoms of dementia: a prospective population-based cohort study from Italy. Eur J Clin Pharmacol. 2014;70:187-95.

[137] Rainer M, Haushofer M, Pfolz H, Struhal C, Wick W. Quetiapine versus risperidone in elderly patients with behavioural and psychological symptoms of dementia: efficacy, safety and cognitive function. Eur Psychiatry. 2007;22:395-403.

[138] Raivio MM, Laurila JV, Strandberg TE, Tilvis RS, Pitkala KH. Neither atypical nor conventional antipsychotics increase mortality or hospital admissions among elderly patients with dementia: a two-year prospective study. Am J Geriatr Psychiatry. 2007;15:416-24.

[139] Rappaport SA, Marcus RN, Manos G, McQuade RD, Oren DA. A randomized, double-blind, placebo-controlled tolerability study of intramuscular aripiprazole in acutely agitated patients with Alzheimer's, vascular, or mixed dementia. J Am Med Dir Assoc. 2009;10:21-7.

[140] Raskind MA, Peskind ER, Wessel T, Yuan W. Galantamine in AD: A 6-month randomized, placebo-controlled trial with a 6-month extension. Neurology. 2000;54:2261-8.

[141] Reisberg B, Doody R, Stoffler A, Schmitt F, Ferris S, Mobius HJ. Memantine in Moderate-to-Severe Alzheimer's Disease. NEJM. 2003;348:1333-41.

[142] Haupt M, Cruz-Jentoft A, Jeste D. Mortality in elderly dementia patients treated with risperidone. J Clin Psychopharmacol. 2006;26:566-70.

[143] Wooltorton E. Risperidone (Risperdal): increased rate of cerebrovascular events in dementia trials. Canadian Medical Association Journal. 2002;167:1269-70.

[144] Herrmann N, Lanctot KL. Do Atypical Antipsychotics Cause Stroke? CNS Drugs. 2005;19:91-103.

[145] Rochon P, Normand S-L, Gomes T, Gill SS, Anderson GM, Melo M, et al. Antipsychotic Therapy and Short-term Serious Events in Older Adults with Dementia. Arch Intern Med. 2008;168:1090-6.

[146] Rockwood K, Mintzer J, Truyen L, Wessel T, Wilkinson D. Effects of a flexible galantamine dose in Alzheimer's disease: a randomised, controlled trial. J Neurol Neurosurg Psychiatry. 2001;71:589-95.

[147] Rogers SL, Doody R, Mohs RC, Friedhoof LT. Donepezil Improves Cognition and Global Function in Alzheimer Disease: A 15-Week, Double-blind, Placebo-Controlled Study. Arch Intern Med. 1998;158:1021-31.

[148] Rogers SL, Farlow M, Doody R, Mohs RC, Friedhoof LT. A 24-week, double-blind, placebo-controlled trial of donepezil in patients with Alzheimer's disease. Neurology. 1998;50:136-45.

[149] Roman GC, Salloway S, Black SE, Royall DR, Decarli C, Weiner MW, et al. Randomized, placebo-controlled, clinical trial of donepezil in vascular dementia: differential effects by hippocampal size. Stroke. 2010;41:1213-21.

[150] Rosenberg PB, Drye LT, Martin BK, Frangakis C, Mintzer J, Weintraub D, et al. Sertraline in the Treatment of Depression in Alzheimer Disease. American Journal of Geriatric Psychiatry. 2010;18:136-45.

[151] Rosenberg PB, Lanctot KL, Drye LT, Herrmann N, Scherer RW, Bachman DL, et al. Safety and Efficacy of Methylphenidate for Apathy in Alzheimer's Disease: A Randomized, Placebo-Controlled Trial. J Clin Psychiatry. 2013;74:810-6.

[152] Rosler M, Anand R, Cicin-Sain A, Gauthier S, Agid Y, Dal-Bianco P, et al. Efficacy and safety of rivastigmine in patients with Alzheimer's disease: international randomised controlled trial. BMJ. 1999;318:633-40.

[153] Rossom RC, Rector TS, Lederle FA, Dysken MW. Are all commonly prescribed antipsychotics associated with greater mortality in elderly male veterans with dementia? J Am Geriatr Soc. 2010;58:1027-34.

[154] Ruths S, Straand J, Nygaard HA, Aarsland D. Stopping antipsychotic drug therapy in demented nursing home patients: a randomized, placebo-controlled study--the Bergen District Nursing Home Study (BEDNURS). Int J Geriatr Psychiatry. 2008;23:889-95.

[155] Sahlberg M, Holm E, Gislason GH, Kober L, Torp-Pedersen C, Andersson C. Association of Selected Antipsychotic Agents With Major Adverse Cardiovascular Events and Noncardiovascular Mortality in Elderly Persons. J Am Heart Assoc. 2015;4:e001666.

[156] Santos-Garcia D, Macias M, Casas-Martinez A, Llaneza M, Abella J, Aneiros A, et al. Descriptive analysis of the use of atypical antipsychotics under compassionate-use in a health area in Ferrol (La Cruna, Spain). Neurologia. 2010;25:300-8.

[157] Saxton J, Hofbauer RK, Woodward M, Gilchrist NL, Potocnik F, Hsu HA, et al. Memantine and functional communication in Alzheimer's disease: results of a 12-week, international, randomized clinical trial. J Alzheimers Dis. 2012;28:109-18.

[158] Scarpini E, Bruno G, Zappala G, Adami M, Richarz U, Gaudig M, et al. Cessation versus continuation of galantamine treatment after 12 months of therapy in patients with Alzheimer's disease: a randomized, double blind, placebo controlled withdrawal trial. J Alzheimers Dis. 2011;26:211-20.

[159] Schneeweiss S, Setoguchi S, Brookhart A, Dormuth C, Wang PS. Risk of death associated with the use of conventional versus atypical antipsychotic drugs among elderly patients. CMAJ. 2007;176:627-32.

[160] Schneider LS, Tariot PN, Dagerman KS, Davis SM, Hsaio JK, Ismael S, et al. Effectiveness of Atypical Antipsychotic Drugs in Patients with Alzheimer's Disease. NEJM. 2006;355:1525-38.

[161] Shin JY, Choi NK, Lee J, Seong JM, Park MJ, Lee SH, et al. Risk of ischemic stroke associated with the use of antipsychotic drugs in elderly patients: a retrospective cohort study in Korea. PLoS One. 2015;10:e0119931.

[162] Sinforiani E, Passoti C, Chiapella L, Malinverni P, Zucchella C. Memantine in Alzheimer's disease: experience in an Alzheimer's disease assessment unit Aging Clinical and Experimental Research. 2012;24:193-6.

[163] Sival RC, Haffmans PM, Jansen PA, Duursma SA, Eikelenboom P. Sodium valproate in the treatment of aggressive behavior in patients with dementia--a randomized placebo controlled clinical trial. Int J Geriatr Psychiatry. 2002;17:579-85.

[164] Sommer OH, Aga O, Cvancarova M, Olsen IC, Selbaek G, Engedal K. Effect of oxcarbazepine in the treatment of agitation and aggression in severe dementia. Dement Geriatr Cogn Disord. 2009;27:155-63.

[165] Sterke CS, van Beeck EF, van der Velde N, Ziere G, Petrovic M, Looman CW, et al. New insights: dose-response relationship between psychotropic drugs and falls: a study in nursing home residents with dementia. J Clin Pharmacol. 2012;52:947-55.

[166] Street J, Scott Clark W, Gannon KS, Cummings J, Bymaster FP, Tamura RN, et al. Olanzapine Treatment of Psychotic and Behavioral Symptoms in Patients with Alzheimer Disease in Nursing Care Facilities. Arch Gen Psychiatry. 2000;57:968-76.

[167] Streim JE, Porsteinsson AP, Breder CD, Swanink R, Marcus R, McQuade R, et al. A Randomized, Double-Blind, Placebo-Controlled Study of Aripiprazole for the Treatment of Psychosis in Nursing Home Patients with Alzheimer Disease. American Journal of Geriatric Psychiatry. 2008;16:537-50.

[168] Sturm AS, Trinkley KE, Porter K, Nahata MC. Efficacy and safety of atypical antipsychotics for behavioral symptoms of dementia among patients residing in long-term care. Int J Clin Pharm. 2018;40:135-42.

[169] Su JA, Chang CC, Wang HM, Chen KJ, Yang YH, Lin CY. Antidepressant treatment and mortality risk in patients with dementia and depression: a nationwide population cohort study in Taiwan. Ther Adv Chronic Dis. 2019;10:2040622319853719.

[170] Suh G-H, Shah A. Effect of antipsychotics on mortality in elderly patients with dementia: a 1-year prospective study in a nursing home. International Psychogeriatrics. 2005;17:429-41.

[171] Sultana J, Chang CK, Hayes RD, Broadbent M, Stewart R, Corbett A, et al. Associations between risk of mortality and atypical antipsychotic use in vascular dementia: a clinical cohort study. Int J Geriatr Psychiatry. 2014;29:1249-54.

[172] Tamimi I, Ojea T, Sanchez-Siles JM, Rojas F, Martin I, Gormaz I, et al. Acetylcholinesterase inhibitors and the risk of hip fracture in Alzheimer's disease patients: a case-control study. J Bone Miner Res. 2012;27:1518-27.

[173] Tamimi I, Nicolau B, Eimar H, Arekunnath Madathil S, Kezouh A, Karp I, et al. Acetylcholinesterase inhibitors and the risk of osteoporotic fractures: nested case-control study. Osteoporosis International. 2018;29:849-57.

[174] Tan ECK, Johnell K, Garcia-Ptacek S, Haaksma ML, Fastbom J, Bell JS, et al. Acetylcholinesterase inhibitors and risk of stroke and death in people with dementia. Alzheimers Dement. 2018;14:944-51.

[175] Tariot PN, Frederiksen K, Erb R, Leibovici A, Podgorski CA, Asnis J, et al. Lack of Carbamazepine Toxicity in Frail Nursing Home Patients: A Controlled Study. JAGS. 1995;43:1026-9.

[176] Tariot PN, Erb R, Podgorski CA, Cox C, Patel S, Jakimovich LJ, et al. Efficacy and Tolerability of Carbamazepine for Agitation and Aggression in Dementia. Am J Psychiatry. 1998;155:54-61.

[177] Tariot PN, Solomon PR, Morris JC, Kershaw P, Lillienfeld S, Ding C. A 5-month, randomized, placebo-controlled trial of galantamine in AD. Neurology. 2000;54:2269-76.

[178] Tariot PN, Cummings J, Katz IR, Mintzer J, Perdomo C, Schwam EM, et al. A Randomized, Double-Blind, Placebo-Controlled Study of the Efficacy and Safety of Donepezil in Patients with Alzheimer's Disease in the Nursing Home Setting. JAGS. 2001;49:1590-9.

[179] Tariot PN, Schneider LS, Mintzer J, Cutler AJ, Cunningham MR, Thomas JW, et al. Safety and Tolerability of Divalproex Sodium in the Treatment of Signs and Symptoms of Mania in Elderly Patients with Dementia: Results of a Double-Blind, Placebo-Controlled Trial. Current Therapeutic Research. 2001;62:51-67.

[180] Tariot PN, Farlow M, Grossberg G, Graham SM, McDonald S, Gergel I. Memantine Treatment in Patients with Moderate to Severe Alzheimer Disease Already Receiving Donepezil: A Randomized Controlled Trial JAMA. 2004;291:317-24.

[181] Tariot PN, Raman R, Jakimovich LJ, Schneider LS, Porsteinsson AP, Thomas A, et al. Divalproex Sodium in Nursing Home Residents with Possible or Probable Alzheimer Disease Complicated by Agitation: A Randomized, Controlled Trial. American Journal of Geriatric Psychiatry. 2005;13:942-9.

[182] Tariot PN, Schneider LS, Katz IR, Mintzer J, Street J, Copenhaver M, et al. Quetiapine Treatment of Psychosis Associated with Dementia: A Double-Blind, Randomized, Placebo-Controlled Clinical Trial. American Journal of Geriatric Psychiatry. 2006;14:767-76.

[183] Tariot PN, Schneider LS, Cummings J, Thomas RG, Raman R, Jakimovich LJ, et al. Chronic Divalproex Sodium to Attenuate Agitation and Clinical Progression of Alzheimer Disease. Arch Gen Psychiatry. 2011;68:853-61.

[184] Teranishi M, Kurita M, Nishino S, Takeyoshi K, Numata Y, Sato T, et al. Efficacy and tolerability of risperidone, yokukansan, and fluvoxamine for the treatment of behavioral and psychological symptoms of dementia: a blinded, randomized trial. J Clin Psychopharmacol. 2013;33:600-7.

[185] Trifiro G, Verhamme KM, Ziere G, Caputi AP, Ch Stricker BH, Sturkenboom MC. All-cause mortality associated with atypical and typical antipsychotics in demented outpatients. Pharmacoepidemiol Drug Saf. 2007;16:538-44.

[186] Torstensson M, Leth-Moller K, Andersson C, Torp-Pedersen C, Gislason GH, Holm EA. Danish register-based study on the association between specific antipsychotic drugs and fractures in elderly individuals. Age Ageing. 2017;46:258-64.

[187] van Dyck CH, Tariot PN, Meyers B, Resnick EM. A 24-week Randomized, Controlled Trial of Memantine in Patients with Moderate-to-severe Alzheimer Disease. Alzheimer Dis Assoc Disord. 2007;21:136-43.

[188] Vercelletto M, Boutoleau-Bretonniere C, Volteau C, Puel M, Auriacombe S, Sarazin M, et al. Memantine in behavioral variant frontotemporal dementia: negative results. J Alzheimers Dis. 2011;23:749-59.

[189] Wang PS, Schneeweiss S, Avorn J, Fischer MA, Mogun H, Solomon DH, et al. Risk of Death in Elderly Users of Conventional vs. Atypical Antipsychotic Medications. N Engl J Med. 2005;353:2335-41.

[190] Watt JA, Gomes T, Bronskill SE, Huang A, Austin PC, Ho JM, et al. Comparative risk of harm associated with trazodone or atypical antipsychotic use in older adults with dementia: a retrospective cohort study. CMAJ. 2018;190:E1376-E83.

[191] Wei YJ, Simoni-Wastila L, Lucas JA, Brandt N. Fall and Fracture Risk in Nursing Home Residents With Moderate-to-Severe Behavioral Symptoms of Alzheimer's Disease and Related Dementias Initiating Antidepressants or Antipsychotics. J Gerontol A Biol Sci Med Sci. 2017;72:695-702.

[192] Wilcock GK, Lillienfeld S, Gaens E. Efficacy and safety of galantamine in patients with mild to moderate Alzheimer's disease: multicentre randomised controlled trial. BMJ. 2000;321:1-7.

[193] Wilcock GK, Mobius HJ, Stoffler A. A double-blind, placebo-controlled multicentre study of memantine in mild to moderate vascular dementia (MMM500). International Clinical Psychopharmacology. 2002;17:297-305.

[194] Wilcock GK, Howe I, Coles H, Lilienfeld S, Truyen L, Zhu Y, et al. A Long-Term Comparison of Galantamine and Donepezil in the Treatment of Alzheimer's Disease. Drugs Aging. 2003;20:777-89.

[195] Wilcock GK, Ballard C, Cooper JA, Loft H. Memantine for Agitation/Aggression and Psychosis in Moderately Severe to Severe Alzheimer's Disease: A Pooled Analysis of 3 Studies. J Clin Psychiatry. 2008;69:341-8.

[196] Wilkinson D, Murray J. Galantamine: a randomized, double-blind, dose comparison in patients with Alzheimer's disease. International Journal of Geriatric Psychiatry. 2001;16.

[197] Wilkinson D, Passmore AP, Bullock R, Hopker SW, Smith R, Potocnik F, et al. A Multinational, Randomised, 12-week, Comparative Study of Donepezil and Rivastigmine in Patients with Mild to Moderate Alzheimer's Disease. Int J Clin Pract. 2002;56:1-6.

[198] Wilkinson D, Doody R, Helme R, Taubman K, Mintzer J, Kertesz A, et al. Donepezil in vascular dementia: A randomized placebo-controlled study. Neurology. 2003;61:479-86.

[199] Wilkinson D, Fox NC, Barkhof F, Phul R, Lemming O, Scheltens P. Memantine and brain atrophy in Alzheimer's disease: a 1-year randomized controlled trial. J Alzheimers Dis. 2012;29:459-69.

[200] Winblad B, Poritis N. Memantine in Severe Dementia: Results of the 9M-Best Study (Benefit and Efficacy in Severely Demented Patients During Treatment with Memantine). International Journal of Geriatric Psychiatry. 1999;14:135-46.

[201] Winblad B, Engedal K, Soininen K, Verhey F, Waldemar G, Wimo A, et al. A 1-year, randomized, placebo-controlled study of donepezil in patients with mild to moderate AD. Neurology. 2001;57:489-95.

[202] Winblad B, Kilander L, Eriksson S, Minthon L, Batsman S, Wetterholm A-L, et al. Donepezil in patients with severe Alzheimer's disease: double-blind, parallel-group, placebo-controlled study. Lancet. 2006;367:1057-65.

[203] Winblad B, Grossberg G, Frolich L, Farlow M, Zechner S, Nagel J, et al. IDEAL: A 6-month, double-blind, placebo-controlled study of the first skin patch for Alzheimer's disease. Neurology. 2007;69:S14-S22.

[204] Wu CY, Hu HY, Chow LH, Chou YJ, Huang N, Wang PN, et al. The Effects of Anti-Dementia and Nootropic Treatments on the Mortality of Patients with Dementia: A Population-Based Cohort Study in Taiwan. PLoS One. 2015;10:e0130993.

[205] Wysowski DK, Baum C, Ferguson WJ, Lundin F, Ng M-J, Hammerstrom T. Sedative-Hypnotic Drugs and the Risk of Hip Fracture. Journal of Clinical Epidemiology. 1996;49:111-3.

[206] Yin Y, Liu Y, Zhuang J, Pan X, Li P, Yang Y, et al. Low-Dose Atypical Antipsychotic Risperidone Improves the 5-Year Outcome in Alzheimer's Disease Patients with Sleep Disturbances. Pharmacology. 2015;96:155-62.

[207] Zhang Z, Yu L, Gaudig M, Schauble B, Richarz U. Galantamine versus donepezil in Chinese patients with Alzheimer's disease: results from a randomized, double-blind study. Neuropsychiatr Dis Treat. 2012;8:571-7.

[208] Zhong KX, Tariot PN, Mintzer J, Minkwitz MC, Devine NA. Quetiapine to Treat Agitation in Dementia: A Randomized, Double-Blind, Placebo-Controlled Study. Current Alzheimer Research. 2007;4:81-93.

[209] Zhu CW, Livote EE, Scarmeas N, Albert M, Brandt J, Blacker D, et al. Long-term associations between cholinesterase inhibitors and memantine use and health outcomes among patients with Alzheimer's disease. Alzheimers Dement. 2013;9:733-40.

[210] Ballard C, Lana MM, Theodoulou M, Douglas S, McShane R, Jacoby R, et al. A Randomised, Blinded, Placebo-Controlled Trial in Dementia Patients Continuing or Stopping Neuroleptics PLOS Medicine. 2008;5:e76.

[211] Malouf R, Birks JS. Donepezil for vascular cognitive impairment (Review). Cochrane Database of Systematic Reviews. 2004.

[212] Kryzhanovskaya LA, Jeste DV, Young CA, Polzer JP, Roddy TE, Jansen JF, et al. A Review of Treatment-Emergent Adverse Events During Olanzapine Clinical Trials in Elderly Patients with Dementia. J Clin Psychiatry. 2006;67:933-45.

[213] Howard RJ, Juszczak E, Ballard CG, Bentham P, Brown RG, Bullock R, et al. Donepezil for the Treatment of Agitation in Alzheimer's Disease. New England Journal of Medicine. 2007;357:1382-92.

[214] McShane R, Areosa SA, Minakaran N. Memantine for dementia (Review). Cochrane Database of Systematic Reviews. 2006.

[215] Farlow M, Graham SM, Alva G. Memantine for the Treatment of Alzheimer's Disease: Tolerability and Safety Data from Clinical Trials. Drug Safety. 2008;31.

[216] Winblad B, Cummings J, Andreasen N, Grossberg G, Onofrj M, Sadowsky C, et al. A six-month double-blind, randomized, placebo-controlled study of a transdermal patch in Alzheimer's disease--rivastigmine patch versus capsule. Int J Geriatr Psychiatry. 2007;22:456-67.

[217] Lyth AL, Gottfries CG. The Clinical Efficacy of Citalopram in Treatment of Emotional Disturbances in Dementia Disorders: A Nordic Multicentre Study. British Journal of Psychiatry. 1990;157:894-901.

[218] Boxer AL, Knopman DS, Kaufer DI, Grossman M, Onyike C, Graf-Radford N, et al. Memantine in patients with frontotemporal lobar degeneration: a multicentre, randomised, double-blind, placebo-controlled trial. The Lancet Neurology. 2013;12:149-56.

[219] Cochrane Handbook for Systematic Reviews of Interventions. In: Higgins JPT, Green S, editors.: The Cochrane Collaboration; 2011.

[220] Wells GA, Gu J, Singla N, Chung F, Pearman MH, Bergese SD. The Newcastle-Ottawa Scale (NOS) for assessing the quality of nonrandomized studies in meta-analyses. Ottawa2008.

[221] EPOC Risk of bias tool In: group CEpaoc, editor.2011.
